# Bio-guided isolation of alpha-glucosidase inhibitory compounds from Vietnamese liverwort *Marchantia polymorpha*: *in vitro* and *in silico* studies†

**DOI:** 10.1039/d3ra07503f

**Published:** 2023-12-05

**Authors:** Ngoc Khanh Van Nguyen, Ho-Duc-Trung Tran, Thuc-Huy Duong, Nguyen Kim Tuyen Pham, Thi Quynh Trang Nguyen, Thi Ngoc Thao Nguyen, Warinthorn Chavasiri, Ngoc-Hong Nguyen, Huu Tri Nguyen

**Affiliations:** a Faculty of Natural Sciences Pedagogy, Sai Gon University 273 An Duong Vuong, Ward 3, District 5 Ho Chi Minh City 70000 Vietnam trihuunguyen@sgu.edu.vn; b Department of Chemistry, Ho Chi Minh City University of Education 280 An Duong Vuong Street, District 5 Ho Chi Minh City 748342 Vietnam; c Faculty of Environment, Sai Gon University 273 An Duong Vuong, Ward 3, District 5 Ho Chi Minh City 70000 Vietnam; d Center of Excellence in Natural Products Chemistry, Department of Chemistry, Faculty of Science, Chulalongkorn University Pathumwan Bangkok 10330 Thailand; e Nanotec-CU Center of Excellence on Food and Agriculture, Department of Chemistry, Faculty of Science, Chulalongkorn University Bangkok 10330 Thailand; f CirTech Institute, HUTECH University 475 A Dien Bien Phu Street, Binh Thanh District Ho Chi Minh City Vietnam nn.hong@hutech.edu.vn

## Abstract

Bio-guided isolation was applied to Vietnamese *Marchantia polymorpha* L. to find alpha-glucosidase inhibition. Fifteen compounds were isolated and structurally determined, including two new compounds, marchatoside (7) and marchanol (8), along with thirteen known compounds: marchantin A (1), isoriccardin C (2), riccardin C (3), marchantin K (4), lunularin (5), 3*R*-(3,4-dimethoxybenzyl)-5,7-dimethoxyphthalide (6), vitexilactone (9), 12-oleanene-3-one (10), 3,11-dioxoursolic acid (11), ursolic acid (12), artemetin (13), kaempferol (14), and quercetin (15). The structures of these compounds were determined through extensive spectroscopic analyses (1D and 2D NMR, HRESIMS, and ECD) and by comparisons to the existing literature. There are five types of carbon skeleton, including bibenzyl (1–5), 3-benzylphthalide (6 and 7), diterpenoid (8 and 9), triterpenoid (10–12), and flavonoid (13–15). Compounds 6–12 were reported for the first time within the genus *Marchantia*. Compounds 1–12 were evaluated for their alpha-glucosidase inhibition. Among them, 1–5 and 10–12 displayed potent inhibition, with IC_50_ values ranging from 28.9 to 130.6 μM, compared to the positive control acarbose 330.9 μM. A kinetic study and molecular docking were also performed to understand the mechanism.

## Abstract 2

Bio-guided isolation was applied to Vietnamese *Marchantia polymorpha* L. to find alpha-glucosidase inhibition.

## Graphical abstract

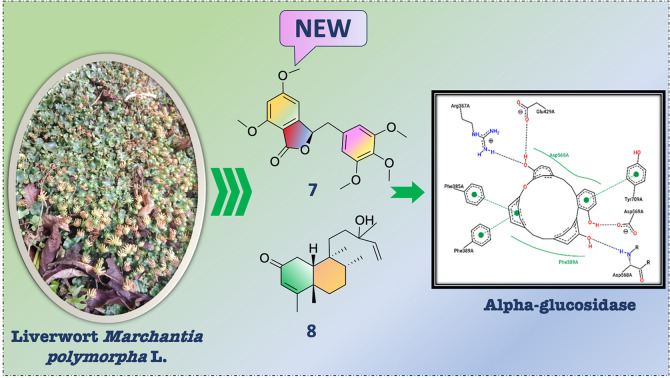

## 1. Introduction

**Fig. 1:**
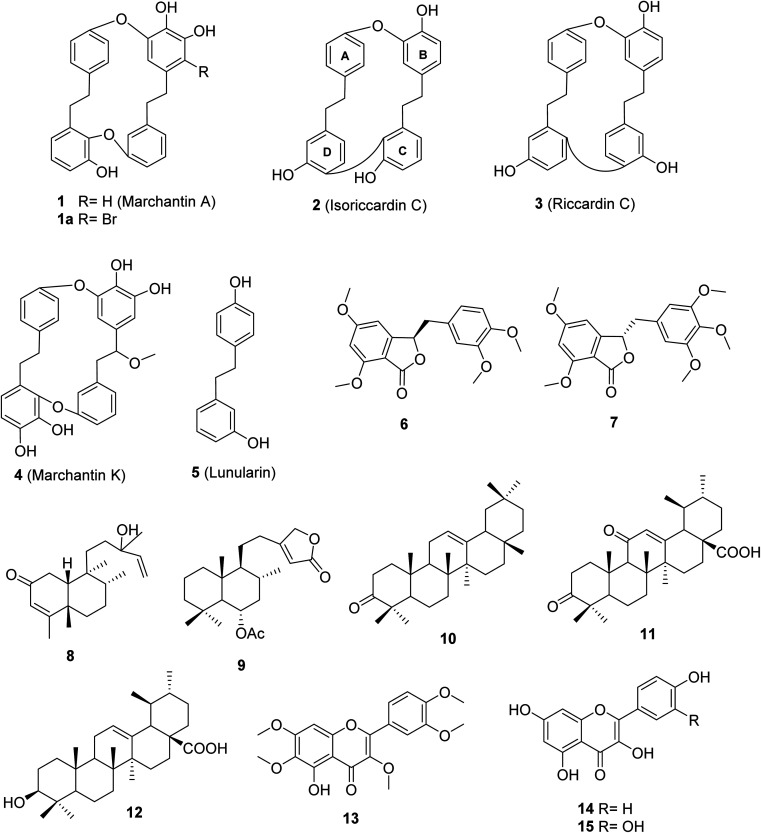
Chemical structures of 1–15.

*Marchantia polymorpha* L. is a liverwort belonging to the family Marchantiaceae, widely distributed in mountainous regions.^1^ It is believed to be a valuable source of traditional medicines in India, China, Europe, and Vietnam.^1,2^ In folk medicine, it has been used to treat inflammation, liver disease, wounds, boils, insect bites, and snake bites.^1^ Chemical data of this liverwort have been comprehensively investigated, indicating the presence of over 50 compounds (Fig. S1†).^3–10^ Among these compounds, 11 known components were found in the native liverwort in Vietnam (Fig. S2†).^11,12^ The major carbon skeletons of these compounds were found to be bis-bibenzyls, sesquiterpenoids, flavonoids, phenolics, coumarins, and glycosides. The extracts of this liverwort showed antioxidant and antibacterial properties, inhibition of tyrosinase and alpha-glucosidase, as well as cytotoxicity against several cancer cell lines.^13–18^ However, little is known about the alpha-glucosidase inhibitors from this medicinal source, with only two reports.^17,18^ These authors conducted the biological evaluation of extracts derived from natural and cultured *M. polymorpha*, indicating the potent inhibition of all prepared extracts. Bio-guided isolation was applied to Vietnamese *M. polymorpha* to find alpha-glucosidase inhibition. Fifteen compounds were isolated (Fig. 1). The structures of these compounds were determined through extensive spectroscopic analyses (1D and 2D NMR, HRESIMS, and ECD). Two new compounds, marchatoside (7) and marchanol (8), together with thirteen known compounds, marchantin A (1), isoriccardin C (2), riccardin C (3), marchantin K (4), lunularin (5), 3*R*-(3,4-dimethoxybenzyl)-5,7-dimethoxyphthalide (6), vitexilactone (9), 12-oleanene-3-one (10), 3,11-dioxoursolic acid (11), ursolic acid (12), artemetin (13), kaempferol (14), and quercetin (15) were structurally determined. A brominated derivative 1a of marchantin A was prepared. All compounds were evaluated for their alpha-glucosidase inhibition. A kinetic study and molecular docking were also performed to understand the mechanism.

## 2. Results and discussion

The crude extract of *M. polymorpha* was prepared using EtOAc. This crude extract was evaluated for alpha glucosidase inhibition, indicating moderate activity with an IC_50_ value of 27.7 ± 1.2 μg mL^−1^. The solvent ethyl acetate was selected for maceration based on the results reported by Tran *et al.* (2018).^16^ These authors prepared four *n*-hexane, chloroform, ethyl acetate, and ethanol extracts from natural *M. polymorpha* and evaluated their alpha-glucosidase inhibition. The ethyl acetate extract showed the strongest activity, with an IC_50_ value of 10.3 μg mL^−1^.^16^ In 2020, this team also evaluated the inhibition of the cultured *M. polymorpha* biomass,^17^ indicating that *n*-hexane extract showed potent inhibition with an IC_50_ value of 11.9 μg mL^−1^.

Two new compounds, marchatoside (7) and marchanol (8), along with thirteen known compounds, marchantin A (1),^18^ isoriccardin C (2),^4^ riccardin C (3),^4,12^ marchantin K (4),^19^ lunularin (5),^12^ 3*R*-(3,4-dimethoxybenzyl)-5,7-dimethoxyphthalide (6),^20^ vitexilactone (9),^21^ 12-oleanene-3-one (10),^22^ 3,11-dioxoursolic acid (11),^23^ ursolic acid (12),^24^ artemetin (13),^25^ kaempferol (14),^13^ and quercetin (15)^12^ were structurally determined. To the best of our knowledge, compounds 7–11 were reported for the first time in liverworts. Isolated compounds were classified as bis-bibenzyl (1–4), bibenzyl (5), 3-benzylphthalide (6 and 7), diterpene (8 and 9), triterpene (10–12), and flavanols (13–15). Many natural 3-benzylphthalide-type compounds have been reported, but little is known about their absolute configuration.^26–29^ 3-Benzylphthalide-type compounds have not been reported in the genus *Marchantia*, but they were found previously in *Frullania* sp. and *Porella perrottetiana* liverworts.^29^ A few clerodane-type and *ent*-labdane-type diterpenoids were previously isolated in liverworts: *Gottschelia schizopleura*^30^ and *Jamesoniella autumnalis*.^31^ However, these types of compound were rarely found in liverworts. Compound 6 was previously isolated from the liverwort *Frullania muscicola*,^20^ but the absolute configuration of C-3 was unknown. ECD data of 6 was recorded, showing a strong negative Cotton effect around 240–250 nm. This CE was consistent with that of julacelide reported in our previous paper,^32^ proposing the absolute C-3 configuration as 3*R*. Comparison of NMR data of known compounds 1–6, 8–11 with those reported in the literature gave consistency (see Tables S1–S12†). Compound 1 was selected for bromination in order to find a better alpha-glucosidase inhibitor. Electrophilic aromatic bromination was applied to 1, yielding product 1a (see Scheme S1†). The chemical structure of 1a was defined using the spectroscopic method (see Table S13†). Unfortunately, compound 1a showed weaker activity than the parent compound 1, thus no further halogenated modifications were conducted on 1.

**Fig. 2:**
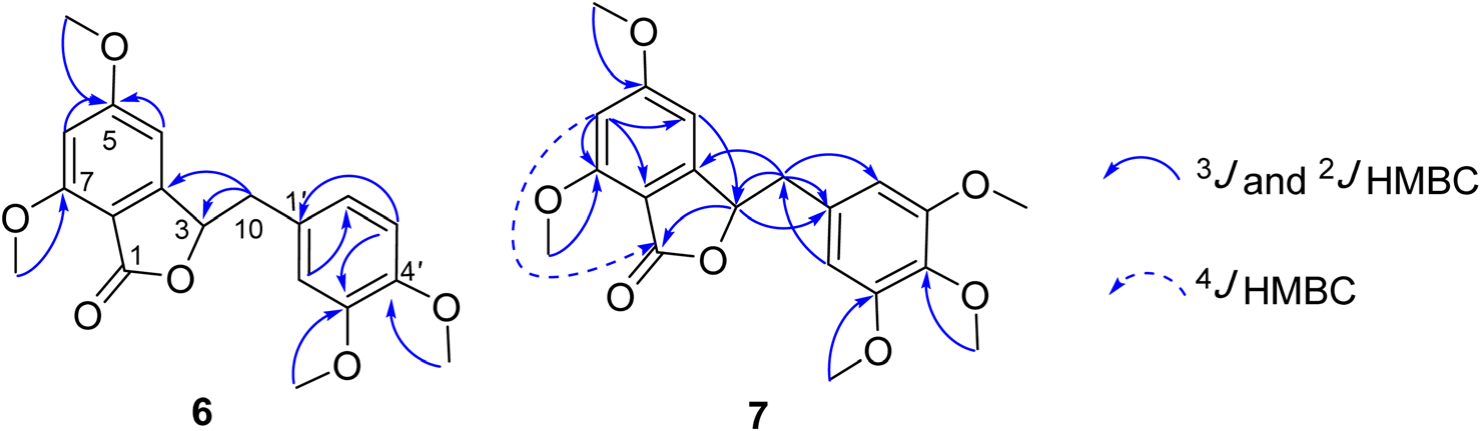
Selected HMBC correlations of 6 and 7.

**Fig. 3:**
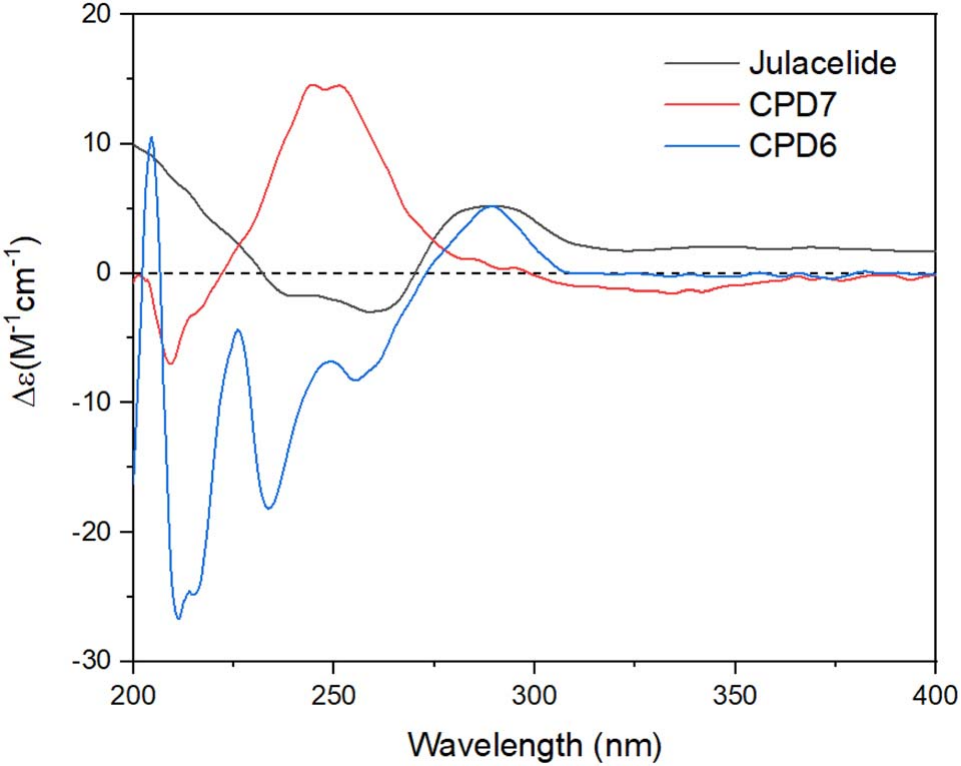
ECD spectra of compounds 6–7 and julacelide.

Compound 7 was obtained as a colorless oil. The molecular formula of 7 was deduced as C_20_H_22_O_7_ from the protonated molecular ion peak at *m*/*z* 375.1397 (calcd for [C_20_H_22_O_7_ + H]^+^, 375.14438) in the HRESI mass spectrum (Fig. S9A†). The ^1^H NMR spectrum showed two aromatic protons of a symmetric 1,3,4,5-tetrasubstituted benzene ring (*δ*_H_ 6.58, s, H-2′ and H-6′), two meta-coupled aromatic protons [*δ*_H_ 6.59 (1H, d, *J* = 1.5, H-4) and 6.54 (1H, d, *J* = 1.5, H-6)], five methoxy groups [*δ*_H_ 3.88 (3H, s, 5-OCH_3_), 3.87 (3H, s, 7-OCH_3_), 3.75 (6H, s, 3′-OCH_3_ and 5′-OCH_3_), and 3.67 (3H, s, 4′-OCH_3_)], and one oxygenated methine [*δ*_H_ 5.62 (1H, dd, *J* = 6.5, 5.5, H-3)] coupled with two protons of one methylene group [*δ*_H_ 3.25 (1H, dd, *J* = 14.5, 5.5, H-10a) and 3.10 (1H, dd, *J* = 14.0, 6.5, H-10b)]. The JMOD and HSQC spectra exhibited 20 carbon resonances attributable to a carboxyl ester group (*δ*_C_ 167.5), four aromatic methine carbons (*δ*_C_ 108.2 × 2, 99.7, and 99.4), one oxygenated methine carbon (*δ*_C_ 80.2), one methylene carbon (*δ*_C_ 41.4), five methoxy carbons (*δ*_C_ 60.5, 56.4, 56.4, 56.4, and 56.2), and eight quaternary carbons [*δ*_C_ 167.4, 160.4, 155.3, 154.1 (× 2), 137.2, 132.6, and 107.8]. The above spectroscopic data indicated that 1 was a 3-benzylphthalide derivative.^32^ In the so-called A-ring, the protons at *δ*_H_ 6.59 (H-4) and *δ*_H_ 6.54 (H-6) gave HMBC correlations to the same carbons at *δ*_C_ 167.4 (C-5), 155.3 (C-9), and 107.8 (C-8) indicated the connection through C-4 to C-9 (Fig. 2). The methoxy groups at *δ*_H_ 3.88 and 3.87 were determined to be located at C-5 and C-7 in this A-ring due to their HMBC correlations to these carbons. The methine group at *δ*_H_ 5.62 was coupled with the methylene CH_2_-10 (*δ*_H_ 3.25 and 3.10) in an ABX system characteristic of 3-benzylphthalide derivatives.^25–28^ Next, the methylene group H_2_-10 gave HMBC correlations to carbons at *δ*_C_ 132.6 (C-1′) and 108.2 (C-2′ and C-6′), and 122.9 (C-6′) indicated the connectivity between A and B-rings through C-3 and C-10 (Fig. 2). The lactonization between C-1 and C-3 was defined by the HMBC correlation of H-3 to C-1 and the downfield chemical shift of H-3.^32^ NMR data of 7 were similar to those of 6 (Table 1). The only difference is the occurrence of the methoxy group at C-3′. The absolute configuration of 7 was defined using ECD data. Particularly, a positive Cotton effect at 260 nm indicated the 3*S* configuration of 7 (Fig. 3). This CE was opposite that of julacelide isolated from the moss *Erythrodontium julaceum*. Combined, the chemical structure of 7 was elucidated as shown in Fig. 1, namely marchatoside.

**Table 1:** ^1^H NMR (500 MHz, *δ*_H_, multi, (*J* in Hz)) and ^13^C NMR (125 MHz) spectral data of 6–7 and julacelide in acetone-*d*_6_

No.	Julacelide		6		7	
	*δ*_H_ (multi, *J* in Hz)	*δ*_C_	*δ*_H_ (multi, *J* in Hz)	*δ*_C_	*δ*_H_ (multi, *J* in Hz)	*δ*_C_
1		166.5		167.1		167.5
2	5.69 (dd, 6.0, 5.5)	80.5	5.85 (t, 6.0)	80.3	5.62 (dd, 6.5, 5.0)	80.2
3	7.05 (dd, 8.0, 2.0)	115.1	6.61 (s)	99.5	6.59 (d, 1.5)	99.7
4	7.65 (dd, 8.5, 8.0)	136.7		166.8		167.4
5	7.05 (dd, 8.0, 2.0)	111.8	6.54 (d, 2.0)	99.6	6.54 (d, 1.5)	99.4
6		159.3		160.3		160.4
7		123.5		105.1		107.8
8		153.2		154.8		155.3
9	3.25 (dd, 14.5, 5.0), 3.12 (dd, 14.0, 6.0)	40.6	3.25 (dd, 14.0, 5.0), 3.10 (dd, 14.0, 6.0)	40.6	3.25 (dd, 14.5, 5.0), 3.10 (dd, 14.5, 6.5)	41.4
1′		129.1		129.3		132.6
2′	6.82 (d, 2.0)	114.6	6.87 (d, 1.5)	114.6	6.58 (s)	108.2
3′		150.0		150.0		154.1
4′		149.3		148.3		137.2
5′	6.80 (d, 8.0)	112.5	6.82 (d, 8.0)	112.6		154.1
6′	6.73 (dd, 8.0, 2.0)	122.9	6.77 (dd, 8.5, 2.0)	122.9	6.57 (s)	108.2
5-OCH_3_			3.87 (s)	56.4	3.88 (s)	56.4
7-OCH_3_	3.89 (s)	56.0	3.89 (s)	56.0	3.87 (s)	56.2
3′-OCH_3_	3.74 (s)	56.0	3.75 (s)	56.3	3.75 (s)	56.4
4′-OCH_3_	3.71 (s)	56.1	3.73 (s)	56.4	3.67 (s)	60.5
5′-OCH_3_					3.75 (s)	56.4

**Table 2:** ^1^H and ^13^C NMR spectral data of 8, (−)-roseostachenone and 13-*epi*-(−)-roseostachenone

No.	8			(−)-Roseostachenone		13-*epi*-(−)-Roseostachenone	
	*δ*_H_ (multi, *J* in Hz) (acetone-*d*_6_, 500 MHz)	*δ*_C_ (acetone-*d*_6_, 125 MHz)	*δ*_H_ (multi, *J* in Hz) (CDCl_3_, 500 MHz)	*δ*_H_ (multi, *J* in Hz) (CDCl_3_, 200 MHz)	*δ*_C_ (CDCl_3_, 50 MHz)	*δ*_H_ (multi, *J* in Hz) (CDCl_3_, 200 MHz)	*δ*_C_ (CDCl_3_, 50 MHz)
1	2.66 (dd, 18.5, 7.0), 2.43 (d, 18.0)	35.7	2.69 (dd, 18.5, 6.5), 2.50 (d, 18.5)	2.28 (m)	34.8	2.28 (m)	34.3
2		198.8			200.4		200.5
3	5.56 (d, 1.0)	129.1	5.84 (brs)	5.67 (m)	125.4	5.66 (d, 1.2)	127.4
4		169.4			172.5		172.6
5		40.1			39.7		39.7
6		37.5			34.8		35.5
7		29.1			26.8		26.8
8		37.4			35.8		35.8
9		40.3			38.3		38.3
10	1.90 (m)	47.9	1.83 (d, 6.5)	1.87 (m)	45.5		45.5
11		31.3			31.1		31.1
12		35.8			35.5		34.7
13		72.8			73.0		73.0
14	5.93 (dd, 17.0, 10.5)	147.1	5.87 (dd, 10.5, 17.5)	5.83 (dd, 10.70, 17.33)	144.9	5.82 (dd, 17.4, 10.7)	144.8
15	5.21 (dd, 17.5, 2.0), 4.98 (dd, 11.0, 2.0)	111.5	5.09 (d, 11.0), 5.20 (d, 16.5)	5.03 (dd, 10.7, 1.0), 5.16 (dd, 17.3, 1.0)	112.0	5.14 (dd, 17.4, 1.3), 5.03 (dd, 10.7, 1.3)	111.9
16	1.24 (s)	28.5	1.22 (s)	1.23 (s)	27.6	1.22 (s)	27.7
17	0.78 (d, 7.0)	16.3	0.77 (d, 7.0)	0.77 (d, 6.5)	15.8	0.79 (d, 6.8)	15.6
18	1.96 (d, 1.0)	20.5	1.94 (d, 1.5)	1.84 (d, 1.0)	18.9	1.83 (d, 1.1)	18.9
19	1.25 (s)	32.3	1.25 (s)	1.06 (s)	18.3	1.06 (s)	18.2
20	0.56 (s)	19.6	0.58 (s)	0.76 (s)	18.0	0.76 (s)	17.9

**Fig. 4:**
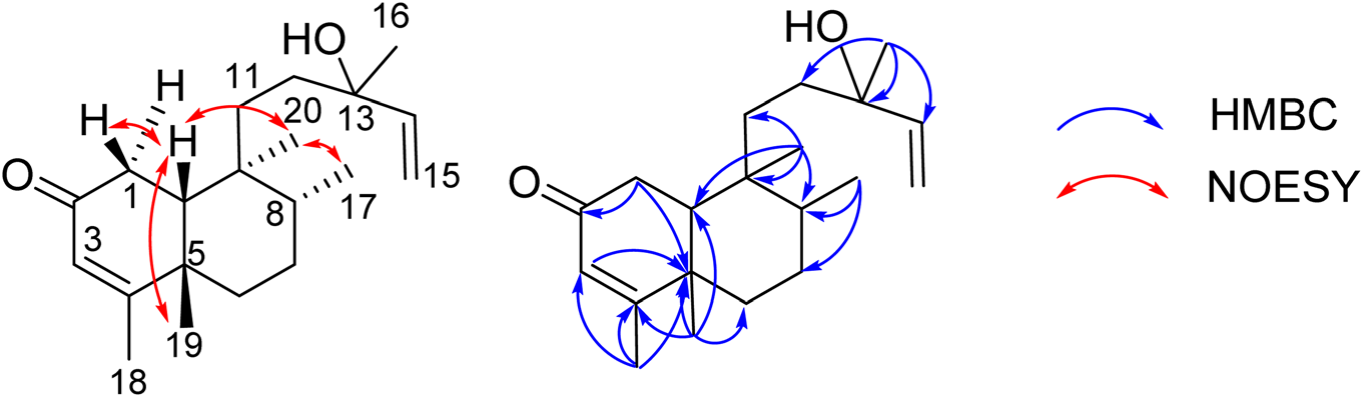
Selected HMBC and NOESY correlations of 8.

Compound 8 was obtained as a colorless oil. The molecular formula of 8 was deduced as C_20_H_32_O_2_ from the sodiated molecular ion peak at *m*/*z* 327.2295 (calcd for [C_20_H_32_O_2_ + Na]^+^, 327.2300) in the HRESI mass spectrum (Fig. S10A†). The ^1^H NMR spectrum showed an olefinic proton (*δ*_H_ 5.56, d, *J* = 1.0, H-3), a vinyl group [*δ*_H_ 5.93 (1H, dd, *J* = 17.0, 1.5, H-14), 5.21 (1H, dd, *J* = 17.5, 2.0, H-15a), and 4.98 (1H, dd, *J* = 11.0, 2.0, H-15b)], five methyl groups [*δ*_H_ 1.96 (d, *J* = 1.0), 1.25, 1.24, 0.78 (d, *J* = 7.0), 0.56], and an ABX system of a methylene [*δ*_H_ 2.66 (1H, dd, *J* = 18.5, 7.0, H-1a) and 2.43 (1H, d, *J* = 18.0, H-1b)] coupled with a methine [*δ*_H_ 1.90 (1H, m, H-10)]. The JMOD and HSQC spectra exhibited a ketone carbonyl group (*δ*_C_ 198.8), two olefinic methine carbons (*δ*_C_ 129.1 and 147.1), one olefinic methylene carbon (*δ*_C_ 111.5), one oxygenated quaternary carbon (*δ*_C_ 72.8), five methyl carbons (*δ*_C_ 32.3, 28.5, 20.5, 19.6, and 16.3), five methylene carbons (*δ*_C_ 37.5, 37.4, 35.8, 35.7, and 31.3), two sp^3^ methine (*δ*_C_ 47.9 and 37.4), and three quaternary carbons (*δ*_C_ 169.4, 40.3, and 40.1). The above spectroscopic data indicated that 1 was a clerodane diterpene.^33–36^ HMBC correlations supported the chemical structure of 8 (Fig. 4). At first, the HMBC correlations of H_3_-18 (*δ*_H_ 1.96) and H_3_-19 (*δ*_H_ 1.25) to C-4 (*δ*_C_ 169.4) and C-5 (*δ*_C_ 40.1), of H-3 (*δ*_H_ 5.56) to C-4 and C-1 (*δ*_C_ 35.7), and of H_2_-1 (*δ*_H_ 2.66 and 2.43) and H-10 (*δ*_H_ 1.99) to C-2 (*δ*_C_ 198.8) defined the structure of the A-ring (Fig. 4). Both methyl H_3_-19 and H_3_-20 (*δ*_H_ 0.56) gave a HMBC correlation to C-10 (*δ*_C_ 47.9), while H-10 gave HMBC correlations to C-19 and C-20, supporting the position of H-20. Moreover, H_3_-20 and H_3_-17 (*δ*_H_ 0.78) gave HMBC correlations to the same carbons, C-8 (*δ*_C_ 37.4) and C-9 (*δ*_C_ 40.3), indicating their locations. A vinyl group was defined to attach at C-13 due to HMBC correlations of H_3_-16 (*δ*_H_ 1.24), H_2_-15 (*δ*_H_ 5.21 and 4.98), and H-14 (*δ*_H_ 5.93) to C-13 (*δ*_C_ 72.8) (Fig. 4). The NMR data of 8 were highly similar to those of (−)-roseostachenone^35^ and 13-*epi*-(−)-roseostachenone,^36^ indicating that they shared the same planar structure. The difference between them was easily observed in the change of the chemical shifts of H_3_-19 (*δ*_H_ 1.25 in 8*vs.* 1.06 in (−)-roseostachenone and 13-*epi*-(−)-roseostachenone) and CH_3_-20 (*δ*_H_ 0.58 in 8*vs.* 0.76 in (−)-roseostachenone and 13-*epi*-(−)-roseostachenone) recorded in the same deuterated solvent (CDCl_3_). This finding proposes that 8 should have a *cis*-clerodane skeleton rather than a *trans*-clerodane of (−)-roseostachenone and 13-*epi*-(−)-roseostachenone. NOESY correlations (in CDCl_3_) of H_3_-17 (*δ*_H_ 0.77)/H_3_-20 (*δ*_H_ 0.58)/H-1a (*δ*_H_ 2.50) indicated their same orientation. In contrast, NOESY correlations of H_3_-19 (*δ*_H_ 1.25)/H-10 (*δ*_H_ 1.83)/H-1b (*δ*_H_ 2.69) indicated their same β-face. The downfield ^13^C chemical shift of C-19 at *δ*_C_ 32.3 also supported the *cis* conjunction of H-10 and H_3_-19.^33,34^ Combined, the chemical structure of 8 was elucidated as shown in Fig. 1, namely marchanol.

### 2.1 Alpha-glucosidase inhibition and kinetic study

Compounds 1–5 and 10–12 displayed strong inhibition, with IC_50_ values ranging from 28.9 to 130.6 μM, while compounds 6–9 showed weak activity (Table 3). Alpha-glucosidase inhibition of compounds 13–15 was previously evaluated in many published reports.^37^ Among the tested compounds, bis-bibenzyl-type compounds 1–4 showed better inhibition. The main difference between 1–4 is in the linkage between C- and D-rings (Fig. 1). The ether connection in 1 and 4 might significantly decrease the activity, compared to the direct connection in 2–3. The activity of 2 was stronger than 3, indicating the important role of connectivity between C- and D-rings. The bis-bibenzyl series showed strong cytotoxicity against many cancer cell lines.^38^ Nevertheless, fewer studies about the alpha-glucosidase inhibition of this series were conducted. Only marchantin C and riccardin C have been investigated so far.^38^ In 2008, Dodo and co-workers reported the alpha-glucosidase inhibition of riccardin C and their oxygenated analogues.^39^ All tested compounds showed potent activity, with IC_50_ values ranging from 4.9 to 49 μM.^39^ While ursolic acid (12) were thought to be a potent alpha-glucosidase inhibitor, the activities of 12-oleanene-3-one (10) and 3,11-dioxoursolic acid (11) have not been reported.

**Fig. 5:**
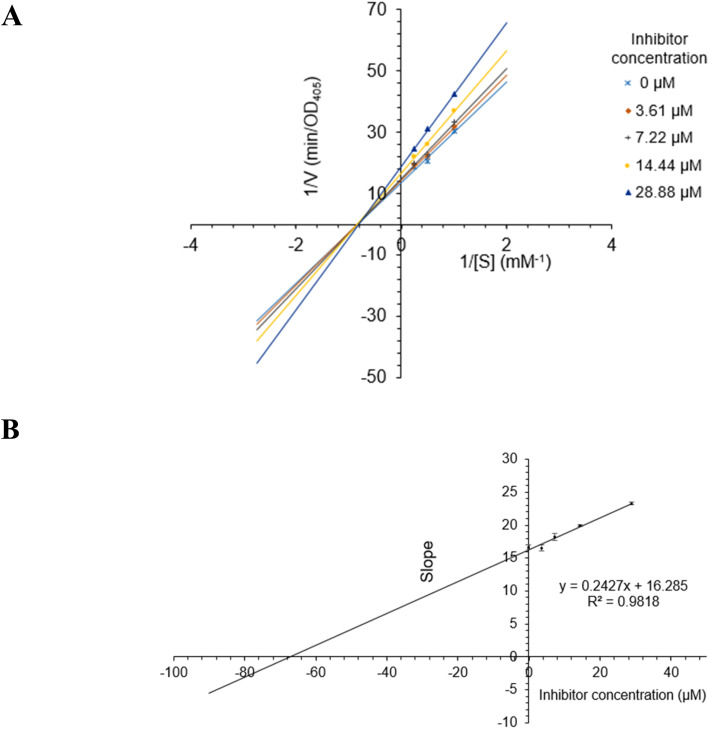
(A) Lineweaver–Burk plot for alpha-glucosidase inhibition by 2. (B) Plots of slope *versus* concentration of 2 for the determination of the inhibition constant *K*_i_.

The most active compound 2 was selected for the enzyme inhibitory kinetic analysis. To examine the inhibition mechanism, activity at concentrations of 0, 3.61, 7.22, 14.44, and 28.88 μM of 2 was recorded. Lineweaver–Burk plots of 2 gave a group of lines with the same Michaelis constant (*K*_m_), intersecting the *y*-axis in the second quadrant (Fig. 5A and B). Increasing inhibitor concentrations cause a decrease in the *V*_max_ of α-glucosidase. The kinetics of enzyme inhibition showed that 2 acted as a non-competitive inhibitor. The *K*_i_ value of 2 was determined as 67.1 ± 1.28 μM, respectively.

**Table 3:** Alpha-glucosidase inhibition (IC_50_) of the test compounds

Bio-source		IC_50_ (μM)
Compound	1	57.3 ± 1.9
	1a	150.6 ± 2.9
	2	28.9 ± 0.2
	3	42.2 ± 1.4
	4	130.6 ± 3.0
	5	129.5 ± 2.0
	6	>200
	7	>200
	8	>200
	9	>200
	10	75.1 ± 1.8
	11	101.0 ± 3.0
	12	86.0 ± 6.9
Positive control	Acarbose	330.9 ± 4.2

### 2.2 *In silico* docking study

**Fig. 6:**
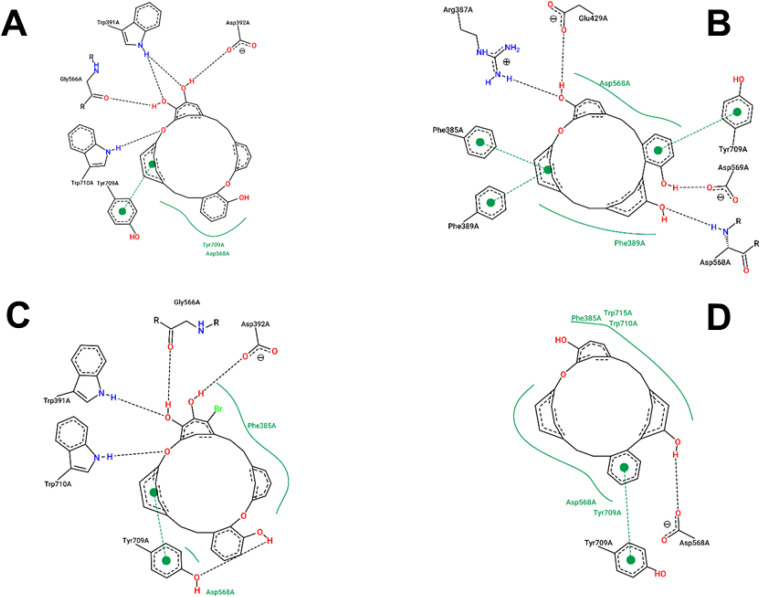
1–4J5T (A), 2–4J5T (B), 1a–4J5T (C) and 3–4J5T (D) interactions.

The estimated binding energies of synthesized compounds to 4J5T protein obtained from 250 runs each per complex have shown highly identical values due to the structural rigidity of those ligands. Meanwhile, the 1 conformational clusters have been computed in the range of −8.17 to −8.26 kcal mol^−1^ for 172 runs out of 250, the 1a, 2, and 3 clusters have been resulted 250 values in the range of 9.37 to −7.74, −9.78 to −9.84, and −9.26 to −9.31 kcal mol^−1^, respectively. The interactions of those complexes were shown in Fig. 6, while the best binding energy from the docking simulation could be found in Table 4. Compound 1 was strongly positioned by five H-bond interactions with 4J5T at Asp 392, Trp 391, Gly 566, Trp 710, Tyr 709. These interactions were also observed in the 1a–4J5T complex, where the addition of the bromo substituent on 1a, although not further generating an H-bond, increased the hydrophobic interaction with the region of Phe 385. That may be the reason why the binding energy of 1a with 4J5T was slightly increased compared to 1.

**Table 4:** The best binding energy between ligands and the 4J5T protein

Ligand	Best binding energy from docking (kcal mol^−1^)
1	−8.26
1a	−9.74
2	−9.84
3	−9.31

The ether structure of 3 showed a slight difference in the order of the aromatic nuclei as well as the deletion of a hydroxy group, which reduced the number of hydrogen bonds but ensured hydrophobic interactions. Only one H-bond with Asp 568 and one pi–pi stacking with Tyr 709 were the major interactions of 3. Regarding 2, the aromatic scaffolds of this structure were arranged in accordance with the pi–pi stacking regions of Phe 385, Phe 389, or Tyr 709. Therefore, unlike 1 or 1a, compound 2 performed four H-bonds with Arg 387, Glu 429, Asp 568, and Asp 569. This framework seemed most suitable for 4J5T inhibition in this series of cyclic ethers.

## 3. Material and methods

### 3.1 General experimental procedures

The NMR spectra were recorded on a Bruker Avance III spectrometer (500 MHz for ^1^H NMR and 125 MHz for ^13^C NMR). The HRESIMS was recorded using an HRESIMS MicrOTOF-Q mass spectrometer on an LC-Agilent 1100 LC-MSD Trap spectrometer. Thin layer chromatography (TLC) was carried out on precoated silica gel 60 F_254_ or silica gel 60 RP-18 F_254S_ (Merck). Spots were visualized by spraying with a 10% H_2_SO_4_ solution, followed by heating. Gravity-column chromatography was performed on silica gel 60 (0.040–0.063 mm, Himedia).

### 3.2 Plant material

*Marchantia polymorpha* L. was collected in Lam Dong Province, Vietnam, from January to March 2023. The specimen was deposited at the herbarium in the laboratory of the Faculty of Chemistry, Ho Chi Minh City University of Education, Vietnam (UE-021). The scientific name was defined by Dr Tram Nguyen Khanh Trinh, Faculty of Biology, Ho Chi Minh City University of Science.

### 3.3 Extraction and isolation of compounds

**Scheme 1:**
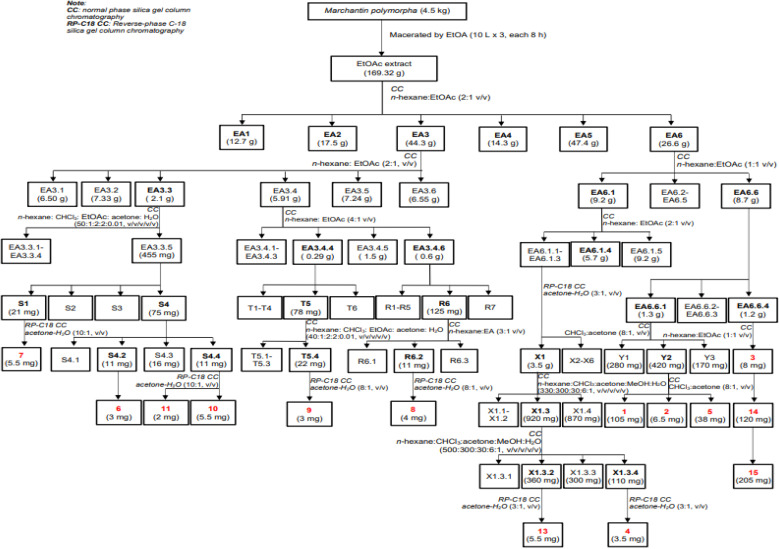
Isolation procedure of compounds 1–15.

Dried, ground powder of *M. polymorpha* (4.5 kg) was extracted with EtOAc (3 × 10 L, each 8 hours) at room temperature. The obtained solution was filtered and evaporated to obtain a crude EtOAc extract (169.3 g). This extract was successively applied to a silica gel column chromatography (CC), isocratically eluted with *n*-hexane : EtOAc (1 : 1, v/v), to obtain six fractions, EA1–EA6. These fractions were evaluated for their alpha-glucosidase inhibition to choose the fractions for further analysis. The isolation of 1–15 were described in Scheme 1 using silica gel CC with various solvent systems.

#### 3.3.1. Marchantin A (1)

Yellow powder. C_28_H_24_O_5_. ^1^H NMR (500 MHz, CDCl_3_, *δ*, ppm, *J*/Hz): 7.13 (1H, dd, *J* = 8.0, 7.5 Hz, H-11), 7.00 (1H, dd, *J* = 8.0, 1.5 Hz, H-10), 6.97 (1H, t, *J* = 7.8, H-13′), 6.91 (1H, d, *J* = 8.5 Hz, H-3), 6.91 (1H, d, *J* = 8.5 Hz, H-5), 6.85 (1H, dd, *J* = 8.0, 1.5 Hz, H-12), 6.57 (1H, d, *J* = 8.0 Hz, H-2), 6.57 (1H, d, *J* = 8.0 Hz, H-6), 6.57 (1H, dd, *J* = 2.5, 2.0 Hz, H-10′), 6.53 (1H, dd, *J* = 8.5, 2.0 Hz, H-12′), 6.46 (1H, d, *J* = 1.5 Hz, H-5′), 6.39 (1H, brd, *J* = 7.5 Hz, H-14′), 5.13 (1H, d, *J* = 1.5 Hz, H-3′), 2.97–3.01 (2H, m, H-7, H-8), 2.78–2.80 (2H, m, H-7′), 2.72–2.74 (2H, m, H-8′). ^13^C NMR (125 MHz, CDCl_3_, *δ*, ppm): 156.8 (C-11′), 153.2 (C-1), 148.7 (C-13), 146.5 (C-2′), 144.3 (C-6′), 143.1 (C-9′), 139.7 (C-14), 139.1 (C-4), 136.2 (C-9), 132.5 (C-4′), 130.8 (C-1′), 129.6 (C-3), 129.6 (C-5), 128.9 (C-13′), 126.0 (C-11), 123.2 (C-14′), 121.9 (C-10), 121.2 (C-2), 121.2 (C-6), 115.5 (C-10′), 114.4 (C-12), 112.0 (C-12′), 109.3 (C-5′), 107.9 (C-3′), 35.5 (C-8′), 35.3 (C-7), 34.1 (C-7′), 30.3 (C-8).

#### 3.3.2. Isoriccardin C (2)

Yellow powder. C_28_H_26_O_4_. ^1^H NMR (500 MHz, acetone-*d*_6_, *δ*, ppm, *J*/Hz): 7.13 (1H, dd, *J* = 7.5, 2.5 Hz, H-5), 7.10 (1H, dd, *J* = 7.5, 2.5 Hz, H-3), 7.305 (1H, t, *J* = 8.0 Hz, H-13′), 6.86 (1H, d, *J* = 7.5 Hz, H-12′), 6.82 (1H, d, *J* = 8.5 Hz, H-2), 6.81 (1H, d, *J* = 8.5 Hz, H-6), 6.81 (1H, m, H-13), 6.74 (1H, d, *J* = 8.0 Hz, H-6′), 6.70 (1H, dd, *J* = 8.0, 2.0 Hz, H-14′), 6.69 (1H, dd, *J* = 8.0, 2.0 Hz, H-5′), 6.65 (1H, brs, H-10), 6.54 (1H, d, *J* = 7.5 Hz, H-14), 5.73 (1H, d, *J* = 1.0 Hz, H-3′), 3.02–3.16 (4H, m, H-7, H-8), 2.50–2.64 (2H, m, H-8′), 2.30–2.33 (2H, m, H-7′). ^13^C NMR (125 MHz, acetone-*d*_6_, *δ*, ppm): 206.1 (C-11′), 177.1 (C-12), 155.5 (C-1), 154.9 (C-11), 145.5 (C-2′), 143.4 (C-1′), 142.1 (C-9), 138.1 (C-4), 138.1 (C-9′), 134.6 (C-4′), 131.9 (C-3), 131.6 (C-13), 131.4 (C-5), 129.1 (C-13′), 122.4 (C-14), 122.1 (C-2), 122.1 (C-6), 121.6 (C-5′), 121.6 (C-14′), 120.5 (C-10′), 117.5 (C-10), 116.4 (C-3′), 116.3 (C-6′), 113.7 (C-12′), 38.5 (C-7′), 37.6 (C-8′), 36.8 (C-8), 35.4 (C-7).

#### 3.3.3. Riccardin C (3)

Yellow powder. C_28_H_26_O_4_. ^1^H NMR (500 MHz, acetone-*d*_6_, *δ*, ppm, *J*/Hz): 7.03 (1H, t, *J* = 8.0 Hz, H-13), 6.95 (1H, t, *J* = 8.0 Hz, H-3), 6.95 (1H, t, *J* = 8.0 Hz, H-5), 6.93 (1H, m, H-10), 6.77 (1H, d, *J* = 8.0 Hz, H-11′), 6.73 (1H, m, H-2), 6.73 (1H, m, H-6), 6.73 (1H, m, H-12), 6.72 (1H, d, *J* = 8.0 Hz, H-6′), 6.50 (1H, m, H-5′), 5.36 (1H, d, H-3′), 2.63–3.03 (m, H-7, H-8, H-7′, H-8′). ^13^C NMR (100 MHz, acetone-*d*_6_, *δ*, ppm): 157.8 (C-11), 154.4 (C-1), 154.1 (C-13′), 148.0 (C-2′), 145.4 (C-1′), 144.3 (C-9), 141.8 (C-9′), 140.8 (C-4), 133.5 (C-4′), 133.4 (C-13), 133.1 (C-11′), 130.3 (C-3), 130.3 (C-5), 128.9 (C-14), 126.8 (C-12′), 122.9 (C-2), 122.9 (C-6), 122.7 (C-10′), 121.5 (C-5′), 117.7 (C-10), 117.4 (C-14′), 116.6 (C-6′), 114.0 (C-12), 38.7 (C-7), 38.5 (C-8′), 37.9 (C-7′), 36.0 (C-8).

#### 3.3.4. Marchantin K (4)

White amorphous powder. C_29_H_28_O_7_. ^1^H NMR (500 MHz, acetone-*d*_6_, *δ*, ppm, *J*/Hz): 6.95 (1H, brd, *J* = 8.0 Hz, H-3), 6.95 (1H, brd, *J* = 8.0 Hz, H-5), 6.88 (1H, t, *J* = 7.5 Hz, H-13′), 6.82 (1H, dd, *J* = 8.0, 1.5 Hz, H-10), 6.93 (1H, m, H-10), 6.76 (1H, d, *J* = 8.5 Hz, H-11), 6.68 (1H, d, *J* = 2.0 Hz, H-10′), 6.59 (1H, d, *J* = 2.0 Hz, H-5′), 6.51 (1H, brd, *J* = 8.0 Hz, H-2), 6.51 (1H, brd, *J* = 8.0 Hz, H-6), 6.45 (1H, dd, *J* = 8.5, 3.0 Hz, H-12′), 6.05 (1H, brd, *J* = 7.0 Hz, H-14′), 4.97 (1H, d, *J* = 2.0 Hz, H-3′), 4.08 (1H, dd, *J* = 9.5, 4.0 Hz, H-7′), 3.20 (s, –OMe), 3.09–3.14 (1H, m, H-7a), 2.95–3.06 (1H, m, H-7b), 2.95–3.06 (2H, m, H-8) 3.00 (1H, m, H-8′a), 2.59 (1H, dd, *J* = 13.0, 10.0 Hz, H-8′b). ^13^C NMR (125 MHz, acetone-*d*_6_, *δ*, ppm): 157.6 (C-11′), 153.9 (C-1), 149.9 (C-2′), 147.3 (C-6′), 147.1 (C-12), 146.3 (C-14), 140.0 (C-4), 139.1 (C-9′), 138.8 (C-13), 136.8 (C-1′) 133.3 (C-4′), 131.4 (C-9), 129.4 (C-3), 129.4 (C-5), 127.8 (C-13′), 125.5 (C-14′), 123.4 (C-2), 121.4 (C-6), 120.9 (C-10), 116.9 (C-10′), 114.4 (C-12′), 112.3 (C-11), 107.6 (C-3′), 106.0 (C-5′), 84.1 (C-7′), 43.8 (C-8′), 35.5 (C-7), 30.1 (C-8).

#### 3.3.5. Lunularin (5)

White amorphous solid. C_14_H_14_O_2_. ^1^H NMR (500 MHz, acetone-*d*_6_, *δ*, ppm, *J*/Hz): 7.07 (1H, t, *J* = 7.8 Hz, H-5′), 7.03 (1H, d, *J* = 8.0 Hz, H-3), 7.03 (1H, d, *J* = 8.0 Hz, H-5), 6.73 (1H, d, *J* = 8.5 Hz, H-2), 6.73 (1H, d, *J* = 8.5 Hz, H-6), 6.69 (1H, d, *J* = 2.0 Hz, H-4′), 6.67 (1H, s, H-2′), 6.63 (1H, d, *J* = 8.0 Hz, H-6′), 2.78 (2H, s, H-7), 2.78 (2H, s, H-8). ^13^C NMR (125 MHz, acetone-*d*_6_, *δ*, ppm): 157.4 (C-3′), 155.5 (C-1), 143.6 (C-1′), 132.6 (C-4), 129.3 (C-5′), 129.1 (C-3), 129.1 (C-5), 119.5 (C-6′), 115.4 (C-3′), 115.0 (C-2), 115.0 (C-6), 112.7 (C-4′), 38.1 (C-8), 36.8 (C-7).

#### 3.3.6. 3*R*-(3,4-Dimethoxybenzyl)-5,7-dimethoxyphthalide (6)

Colorless oil. C_19_H_20_O_6_. ^1^H NMR (500 MHz, acetone-*d*_6_, *δ*, ppm, *J*/Hz): 6.87 (1H, d, *J* = 1.5 Hz, H-2′), 6.82 (1H, d, *J* = 8.0 Hz, H-5′), 6.77 (1H, dd, *J* = 8.5, 2.0 Hz, H-6′), 6.61 (1H, s, H-4), 6.54 (1H, d, *J* = 2.0 Hz, H-6), 5.85 (1H, t, *J* = 6.0 Hz, H-3), 3.89 (3H, s, 7-OCH_3_), 3.87 (3H, s, 5-OCH_3_), 3.75 (3H, s, 3′-OCH_3_), 3.73 (3H, s, 4′-OCH_3_), 3.25 (1H, dd, *J* = 14.0, 5.0 Hz, H-10a), 3.10 (1H, dd, *J* = 14.0, 5.0 Hz, H-10b). ^13^C NMR (125 MHz, acetone-*d*_6_, *δ*, ppm): 167.1 (C-1), 167.1 (C-5), 160.3 (C-7), 154.8 (C-9), 150.0 (C-3′), 148.3 (C-4′), 129.3 (C-1′), 122.9 (C-6′), 114.6 (C-2′), 112.6 (C-5′), 105.1 (C-8), 99.6 (C-6), 99.5 (C-4), 99.5 (C-3), 56.4 (5-OCH_3_), 56.4 (4′-OCH_3_), 56.3 (3′-OCH_3_), 56.0 (7-OCH_3_).

#### 3.3.7. Marchatoside (7)

Colorless oil. C_20_H_22_O_7_. HRESIMS *m*/*z*: 375.1397 [M + H]^+^ (calcd for C_20_H_23_O_7_^+^, 375.1443). ^1^H NMR (500 MHz, acetone-*d*_6_, *δ*, ppm, *J*/Hz) and ^13^C NMR (125 MHz, acetone-*d*_6_, *δ*, ppm) data: see Table 1.

#### 3.3.8. Marchanol (8)

Colorless oil. C_20_H_32_O_2_. HRESIMS *m*/*z*: 327.2295 [M + Na]^+^ (calcd for C_20_H_32_O_2_Na^+^, 327.2300). ^1^H NMR (500 MHz, acetone-*d*_6_, *δ*, ppm, *J*/Hz) and ^13^C NMR (125 MHz, acetone-*d*_6_, *δ*, ppm) data: see Table 2.

#### 3.3.9. Vitexilactone (9)

Colorless oil. C_22_H_34_O_4_. ^1^H NMR (500 MHz, acetone-*d*_6_, *δ*, ppm, *J*/Hz): 5.86 (1H, p, *J* = 1.5, 1.5, 2.0, 2.0 Hz, H-14), 5.34 (1H, q, *J* = 3.0, 3.0, 2.5 Hz, H-6), 4.85 (1H, d, *J* = 2.0 Hz, H-16), 2.61 (2H, m, H-12), 2.17 (1H, m, H-8), 2.02 (1H, m, H-9), 2.00 (1H, s, H-2′), 1.85 (1H, q, *J* = 6.5, 4.5 3.0 Hz, H-11a), 1.82 (1H, q, *J* = 4.0, 4.0, 6.5 Hz, H-11b), 1.76 (1H, d, 2.5 Hz, H-5), 1.71–1.50 (2H, m, H-2), 1.47–1.44 (2H, m, H-7), 1.43–1.32 (2H, m, H-1), 1.37–1.24 (2H, m, H-3), 1.30 (3H, s, 20-CH_3_), 1.02 (3H, s, 19-CH_3_), 0.93 (3H, s, 18-CH_3_), 0.92 (3H, s, 17-CH_3_). ^13^C NMR (125 MHz, acetone-*d*_6_, *δ*, ppm): 174.0 (C-15), 173.4 (C-13), 170.5 (C-1′), 114.8 (C-14), 73.8 (C-16), 70.4 (C-6), 44.7 (C-10), 45.6 (C-5), 43.0 (C-3), 37.4 (C-7), 36.9 (C-9), 34.7 (C-4), 34.0 (C-18), 32.8 (C-8), 32.6 (C-11), 32.5 (C-1), 26.0 (C-12), 24.1 (C-19), 21.8 (C-2′), 19.6 (C-2), 19.5 (C-20), 16.4 (C-17).

#### 3.3.10. 12-Oleanene-3-one (10)

Yellow amorphous powder. C_20_H_20_O_8_. ^1^H NMR (500 MHz, acetone-*d*_6_, *δ*, ppm, *J*/Hz): 5.24 (1H, t, *J* = 4.0 Hz, H-12), 2.31 (1H, m, H-9), 1.19 (3H, s, H-27), 1.10 (3H, s, H-25), 1.06 (3H, s, H-26), 1.05 (3H, s, H-24), 1.03 (3H, s, H-23), 0.89 (3H, s, H-29), 0.86 (3H, s, H-30), 0.84 (3H, s, H-28). ^1^H NMR (500 MHz, CDCl_3_, *δ*, ppm, *J*/Hz): 5.21 (1H, t, *J* = 3.5, 4.0 Hz, H-12), 2.37 (1H, m, H-9), 1.25 (3H, s, H-27), 1.07 (3H, s, H-24), 1.06 (3H, s, H-23), 1.02 (3H, s, H-25), 1.00 (3H, s, H-26), 0.92 (3H, s, H-29), 0.87 (3H, s, H-30), 0.84 (3H, s, H-28). ^13^C NMR (125 MHz, acetone-*d*_6_, *δ*, ppm): 216.2 (C-3), 146.0 (C-13), 122.8 (C-12), 56.0 (C-5), 47.8 (C-9), 47.7 (C-4), 47.3 (C-19), 47.0 (C-18), 41.9 (C-14), 40.0 (C-7), 39.8 (C-1), 37.1 (C-22), 35.5 (C-10), 34.7 (C-2), 34.7 (C-21), 33.1 (C-7), 32.3 (C-17), 32.3 (C-30), 31.3 (C-20), 28.9 (C-28), 26.9 (C-16), 26.4 (C-15), 26.4 (C-23), 24.6 (C-27), 23.7 (C-29), 23.7 (C-11), 21.9 (C-24), 20.4 (C-6), 17.3 (C-26), 15.6 (C-25).

#### 3.3.11. 3,11-Dioxoursolic acid (11)

White amorphous powder. C_30_H_44_O_4_. ^1^H NMR (400 MHz, CDCl_3_, *δ*, ppm, *J*/Hz): 5.63 (1H, s, H-12), 2.39 (1H, s, H-9), 1.31 (3H, s, H-27), 1.24 (3H, s, H-26), 1.02 (3H, s, H-25), 0.97 (3H, d, *J* = 8.0 Hz, H-30), 0.95 (3H, s, H-24), 0.90 (3H, s, H-23), 0.87 (3H, d, *J* = 8.0 Hz, H-29). ^13^C NMR (100 MHz, acetone-*d*_6_, *δ*, ppm): 217.4 (C-3), 199.5 (C-11), 182.8 (C-28), 163.3 (C-13), 130.8 (C-12), 60.8 (C-9), 55.5 (C-5), 52.6 (C-8), 47.9 (C-4), 47.6 (C-17), 44.0 (C-14), 41.5 (C-19), 41.5 (C-20), 39.9 (C-8), 38.7 (C-1), 36.9 (C-10), 36.1 (C-22), 34.3 (C-2), 32.5 (C-7), 28.6 (C-15), 26.6 (C-21), 26.6 (C-23), 23.8 (C-16), 23.7 (C-29), 21.2 (C-24), 21.1 (C-27), 19.0 (C-6), 18.8 (C-26), 17.2 (C-30), 15.7 (C-25).

#### 3.3.12. Ursolic acid (12)

White amorphous powder. C_30_H_48_O_3_. ^1^H NMR (500 MHz, CDCl_3_, *δ*, ppm, *J*/Hz): 5.26 (1H, t, *J* = 3.5 Hz, H-12), 3.22 (1H, dd, *J* = 7.0, 5.0, 4.0 Hz, H-3), 2.19 (1H, d, *J* = 11.5, H-18), 1.02 (3H, s, H-27), 0.92 (3H, s, H-23), 0.86 (3H, d, *J* = 6.5 Hz, H-29), 0.85 (3H, s, H-26), 0.80 (3H, s, H-25), 0.77 (3H, d, *J* = 4.0 Hz, H-30), 0.77 (3H, s, H-24). ^13^C NMR (125 MHz, CDCl_3_, *δ*, ppm): 182.8 (C-28), 138.1 (C-13), 126.0 (C-12), 79.2 (C-3), 55.4 (C-5), 53.0 (C-18), 48.0 (C-17), 47.7 (C-9), 42.2 (C-14), 41.7 (C-19), 39.6 (C-8), 39.6 (C-20), 39.0 (C-4), 38.9 (C-1), 36.8 (C-10), 36.8 (C-22), 33.2 (C-7), 30.8 (C-21), 30.8 (C-15), 28.3 (C-23), 27.4 (C-2), 24.4 (C-16), 23.7 (C-11), 23.7 (C-27), 21.3 (C-30), 18.5 (C-6), 17.2 (C-26), 17.1 (C-29), 15.8 (C-25), 15.6 (C-24).

#### 3.3.13. Artemetin (13)

Yellow amorphous powder. C_20_H_20_O_8_. ^1^H NMR (500 MHz, acetone-*d*_6_, *δ*, ppm, *J*/Hz): 12.69 (1H, s, 5-OH), 7.78 (1H, d, *J* = 2.0 Hz, H-2′), 7.76 (1H, dd, *J* = 3.0, 2.0 Hz, H-6′), 7.14 (1H, d, *J* = 8.5 Hz, H-5′), 6.81 (1H, s, H-8), 3.96 (3H, s, 6-OCH_3_), 3.92 (3H, s, 3-OCH_3_), 3.90 (6H, s, 7-OCH_3_, 3′-OCH_3_), 3.80 (3H, s, 4′-OCH_3_). ^13^C NMR (125 MHz, acetone-*d*_6_, *δ*, ppm): 178.9 (C-4), 159.3 (C-7), 155.8 (C-2), 152.7 (C-5), 152.3 (C-8a), 152.0 (C-4′), 149.2 (C-3′), 138.6 (C-3), 132.3 (C-6), 122.8 (C-1′), 122.1 (C-2′), 111.8 (C-6′), 111.3 (C-5′), 106.2 (C-4a), 90.8 (C-8), 59.7 (7-OCH_3_), 59.4 (3-OCH_3_), 55.9 (6-OCH_3_), 55.4 (3′-OCH_3_), 55.3 (4′-OCH_3_).

#### 3.3.14. 5′-Bromomarchantin A (1a)

Colorless oil. C_28_H_23_BrO_5_. HRESIMS *m*/*z*: 601.0543 [M − H]^−^ (calcd for C_28_H_22_BrO_5_^−^, 517, 06506). ^1^H NMR (500 MHz, acetone-*d*_6_, *δ*, ppm, *J*/Hz): 7.10 (1H, t, *J* = 7.8 Hz, H-13′), 7.04 (1H, dd, *J* = 8.0, 1.5 Hz, H-12′), 7.00 (1H, t, *J* = 8.0 Hz, H-11), 6.96 (1H, d, *J* = 8.0 Hz, H-5), 6.96 (1H, d, *J* = 8.0 Hz, H-3), 6.82 (1H, dd, *J* = 8.0, 1.5 Hz, H-10), 6.61 (1H, dd, *J* = 8.5, 2.5 Hz, H-12), 6.50 (1H, d, *J* = 7.5 Hz, H-2), 6.50 (1H, d, *J* = 7.5 Hz, H-6), 6.28 (1H, d, *J* = 7.5 Hz, H-10′), 6.28 (1H, d, *J* = 7.5 Hz, H-14′), 5.34 (1H, s, H-3′), 3.02–3.06 (4H, m, H-7), 3.02–3.06 (4H, m, H-8), 2.91–2.93 (2H, m, H-7′), 2.91–2.93 (2H, m, H-8′). ^13^C NMR (125 MHz, CDCl_3_, *δ*, ppm): 158.7 (C-11′), 154.1 (C-1), 147.0 (C-2′), 144.5 (C-6′), 142.4 (C-14), 142.4 (C-9′), 139.5 (C-1′), 137.1 (C-4), 133.8 (C-4′), 130.7 (C-9), 130.5 (C-13′), 129.1 (C-3), 129.1 (C-5), 126.4 (C-14′), 122.1 (C-11), 121.8 (C-2), 121.8 (C-6), 121.5 (C-10), 118.5 (C-13), 116.5 (C-12′), 115.2 (C-10′), 113.8 (C-12), 109.6 (C-3′), 105.0 (C-5′), 35.7 (C-7), 35.7 (C-8′), 34.8 (C-7′), 34.1 (C-8).

### 3.4 Alpha-glucosidase inhibition assay

Evaluation of the inhibitory activity of 1–12 against yeast alpha-glucosidase followed a previous procedure.^37^

### 3.5 Kinetic study of α-glucosidase inhibition of 2

The mechanisms of inhibition of alpha-glucosidase by 2 were determined by Lineweaver–Burk plots (Microsoft Excel 2010, Washington, USA), using methods similar to those reported in the literature.^40^ Enzyme inhibition due to various concentrations of 2 was evaluated by monitoring the effects of different concentrations of the substrate. For Lineweaver–Burk double reciprocal plots 1/enzyme velocity (1/*V*) *vs.* 1/substrate concentration (1/[S]), the inhibition type was determined using various concentrations of *p*NPG (1 mM, 2 mM, and 4 mM) as a substrate in the presence of different concentrations of the test compound (0, 3.61, 7.22, 14.44, and 28.88 μM for 2). The experiments were carried out in three replicates. The mixtures were incubated at 37 °C, and the optical density was measured at 405 nm every 1 min for 30 min with the ELx800 Absorbance Microplate Reader (BioTek Instruments, Inc., Vermont, USA). The optimal concentrations of the tested compound were chosen based on the IC_50_ value. The inhibition constants were obtained graphically from secondary plots (Microsoft Excel 2010, Washington, USA).

### 3.6 *In silico* molecular docking model

The 4J5T PDB structure from the Protein Data Bank was used to represent alpha-glucosidase in the docking study. The proteins and ligands were processed to calculate hydrogen bonds and Gasteiger–Marsili charges,^41^ then converted into PDBQT format using AutodockTools. Herein, AutoDock 4.2 was employed for the simulations of protein–ligand complexes. The docking study was modeled with 250 genetic algorithm runs with 25 000 000 maximum number of evals (long mode). The lowest binding energies among the conformational clusters were extracted as the best-estimated docking value. The interactions of the docked conformations were analyzed *via* the Poseview tool.^42^

## 4. Conclusions

Two new compounds, marchatoside (7) and marchanol (8), were isolated from the liverwort *Marchantia polymorpha*. Additionally, thirteen known compounds, including marchantin A (1), isoriccardin C (2), riccardin C (3), marchantin K (4), lunularin (5), 3*R*-(3,4-dimethoxybenzyl)-5,7-dimethoxyphthalide (6), vitexilactone (9), 12-oleanene-3-one (10), 3,11-dioxoursolic acid (11), ursolic acid (12), artemetin (13), kaempferol (14), and quercetin (15), were also isolated and their structures were determined using a bio-guided procedure. Compounds 1–12 were evaluated for their alpha-glucosidase inhibition. The most active compound, compound 2, was subsequently chosen for a kinetic study. It was determined to be of the non-competitive type. The inhibitory mechanism of compounds 1–4 was confirmed through a molecular docking study.

## Conflicts of interest

No potential conflict of interest was reported by the authors.

## Supplementary Material

RA-013-D3RA07503F-s001
