# Supplementary material for: Bio-guided isolation of alpha-glucosidase inhibitory compounds from Vietnamese liverwort Marchantia polymorpha: in vitro and in silico studies
Source: RSC Adv. 2023 Dec 5;13(50):35481–92. doi: 10.1039/d3ra07503f (PMC10697071; doi:10.1039/d3ra07503f)
Supplement: RA-013-D3RA07503F-s001 [file RA-013-D3RA07503F-s001.pdf]

Electronic Supplementary Material (ESI) for RSC Advances.
This journal is © The Royal Society of Chemistry 2023

Table S-1. ^{1}H NMR (500 MHz, δ_H, multi, (*J* in Hz) and ^{13}C NMR (125 MHz) data of compound **1** in $CDCl_3$

No.	**1**		**Marchantin A**	
	^{1}H-NMR ($CDCl_3$, 500 MHz)	^{13}C-NMR ($CDCl_3$, 125 MHz)	^{1}H-NMR ($CDCl_3$, 500 MHz)	^{13}C-NMR ($CDCl_3$, 125 MHz)
1	–	153.2	–	152.9
2	6.57 (1H, *d*, 8.0)	121.2	6.58 (1H, *d*, 8.5)	121.2
3	6.91 (1H, *d*, 8.5)	129.6	6.93 (1H, *d*, 8.5)	129.5
4	–	139.1	–	139.0
5	6.91 (1H, *d*, 8.5)	129.6	6.93 (1H, *d*, 8.5)	129.5
6	6.57 (1H, *d*, 8.0)	121.2	6.58 (1H, *d*, 8.5)	121.2
7	2.97–3.01 (2H, *m*)	35.3	2.96–3.01 (2H, *m*)	35.2
8	2.97–3.01 (2H, *m*)	30.3	2.96–3.01 (2H, *m*)	30.2
9	–	136.2	–	136.1
10	7.00 (1H, *dd*, 8.0, 1.5)	121.9	7.02 (1H, *dd*, 7.8, 1.5)	121.9
11	7.13 (1H, *dd*, 8.0, 7.5)	126.0	7.15 (1H, *t*, 7.8)	126.0
12	6.85 (1H, *dd*, 8.0, 1.5)	114.4	6.87 (1H, *dd*, 7.8, 1.5)	114.3
13	–	148.7	–	148.6
14	–	139.7	–	139.6
1′	–	130.8	–	130.6
2′	–	146.5	–	146.4
3′	5.13 (1H, *d*, 1.5)	107.9	5.13 (1H, *d*, 2.0)	107.9
4′	–	132.5	–	132.4
5′	6.46 (1H, *d*, 1.5)	109.3	6.47 (1H, *d*, 2.0)	109.3
6′	–	144.3	–	144.1
7′	2.78–2.80 (2H, *m*)	34.1	2.72–2.78 (2H, *m*)	34.0
8′	2.72–2.74 (2H, *m*)	35.5	2.72–2.78 (2H, *m*)	35.4
9′	–	143.1	–	143.0
10′	6.57 (1H, *dd*, 2.5, 2.0)	115.5	6.85 (1H, *t*, 2.0)	115.4
11′	–	156.8	–	156.6
12′	6.53 (1H, *dd*, 8.5, 2.0)	112.0	6.55 (1H, *dd*, 7.8, 2.1)	112.0
13′	6.97 (1H, *t*, 7.8)	128.9	6.98 (1H, *t*, 7.8)	128.8
14′	6.39 (1H, *brd*, 7.5)	123.2	6.41 (1H, *brd*, 7.8)	123.1

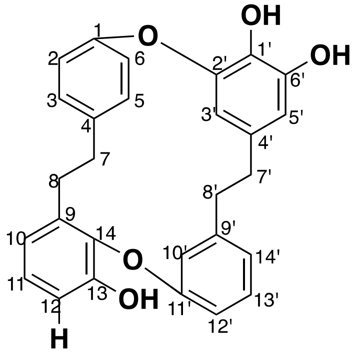

Marchantin A

Table S-2. ^{1}H NMR (500 MHz, δ_H, multi, (*J* in Hz) and ^{13}C NMR (125 MHz) data of compound **2** in $CDCl_3$

**No.**	**2**		**Riccardin C**	
	^{1}H-NMR (Acetone-d_6, 500 MHz)	^{13}C-NMR (Acetone-d_6, 125 MHz)	^{1}H-NMR ($CDCl_3$, 500 MHz)	^{13}C-NMR ($CDCl_3$, 125 MHz)
1	–	154.4	–	152.5
2	6.73 (1H, *m*)	122.9	6.72 – 6.80[b]	122.3
3	6.95 (1H, *d*, 8.0)	130.3	6.87 (*brs*)	129.2
4	–	140.8	–	139.8
5	6.95 (1H, *d*, 8.0)	130.3	6.87 (*brs*)	129.2
6	6.73 (1H, *m*)	122.9	6.72 – 6.80[b]	122.3
7	2.63-3.03	38.7	2.88 (*m*)	38.1
			2.95 (*m*)	36.1
8		36.0	2.23 – 2.75[b]	35.0
			3.03 (*m*)	117.5
9	–	144.3	–	143.7
10	6.93 (1H, *m*)	117.7	6.96 (*d*, 2.9)	117.5
11	–	157.8	–	155.9
12	6.73 (1H, *m*)	114.0	6.79 (*dd*, 8.6, 2.9)	114.3
13	7.03 (1H, *d*, 8.0)	133.4	7.03 (*d*, 8.6)	132.8
14	–	128.9	–	128.2
1′	–	145.4	–	143.7
2′	–	148.0	–	146.3
3′	5.36 (1H, *d*, 1.5)	117.4	5.35 (*d*, 2.0)	116.0
4′	–	133.5	–	133.1
5′	6.50 (1H, *m*)	121.5	6.73 (*dd*, 8.1, 2.0)	122.1
6′	6.72 (1H, *d*, 8.0)	116.6	6.92 (*d*, 8.1)	114.9
7′	2.63-3.03	37.9	2.23 – 2.75[b]	37.1
8′		38.5	2.23 – 2.75[b]	37.6
9′	–	141.8	–	141.9
10′	6.13 (1H, *d*, 8.0, 2.0)	122.7	6.23 (*dd*, 7.8, 1.7)	121.7
11′	6.77 (1H, *d*, 8.0)	133.1	6.77 (*d*, 7.8)	131.4
12′	–	126.8	–	124.4
13′	–	154.1	–	151.8
14′	6.36 (1H, *d*, 1.5)	117.0	6.39 (*d*, 1.7)	116.0

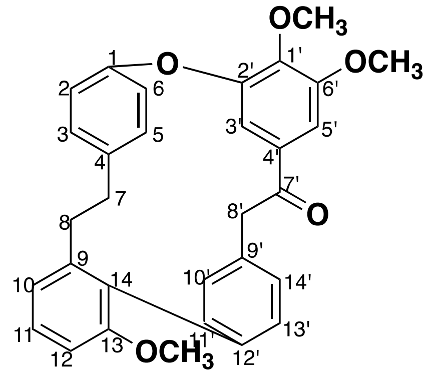

Riccardin C

Table S-3. ^{1}H NMR (500 MHz, δ_H, multi, (*J* in Hz) and ^{13}C NMR (125 MHz) data of compound **3** in acetone-d_6.

**No.**	**3**		**Isoriccardin C**	
	^{1}H-NMR (Acetone-d_6, 500 MHz)	^{13}C-NMR (Acetone-d_6, 125 MHz)	^{1}H-NMR ($CDCl_3$, 500 MHz)	^{13}C-NMR ($CDCl_3$, 125 MHz)
1	–	155.5	–	153.2
2	6.82 (1H, *d,* 8.5)	122.1	6.84 (1H, *dd*, 8.3, 1.7)	121.7
3	7.10 (1H, *dd*, 7.5, 2.5)	131.9	7.08 (1H, *dd*, 8.3, 1.7)	130.8
4	–	138.1	–	137.2
5	7.13 (1H, *dd*, 7.5, 2.5)	131.4	7.13 (1H, *dd*, 8.3, 1.7)	130.3
6	6.81 (1H, *d,* 8.5)	122.1	6.89 (1H, *dd*, 8.3, 1.7)	121.7
7	3.02-3.16 (4H, *m*)	35.4	3.13 (4H, *m*)	34.9
8		36.8	2.96 – 3.01 (2H, *m*)	36.1
9	–	142.1	–	143.3
10	6.65 (1H, *brs*)	117.5	6.77 (1H, *brd*, 1.3)	116.6
11	–	154.9	–	153.5
12	–	177.1	–	117.1
13	6.81 (1H, *m*)	131.6	6.91 (1H, *d*, 7.8)	130.5
14	6.54 (1H, *d*, 7.5)	122.4	6.65 (1H, *dd*, 7.8, 1.3)	122.6
1′	–	143.4	–	143.6
2′	–	145.5	–	147.8
3′	5.73 (1H, *d*, 1.0)	116.4	5.59 (1H, *d*, 2.0)	114.7
4′	–	134.6	–	133.6
5′	6.69 (1H, *dd*, 8.0, 2.0)	121.6	6.68 (1H, *dd*, 8.1, 2.0)	121.6
6′	6.74 (1H, *d*, 8.0)	116.3	6.83 (1H, *d*, 8.1)	114.6
7′	2.30-2.33 (2H, *m*)	38.5	2.50 (2H, *m*)	38.0
8′	2.50 (1H, *m*)<br>2.64 (1H, *m*)	37.6	2.29 (2H, *m*)	36.5
9′	–	138.1	–	142.9
10′	–	120.5	–	120.5

11′	–	206.1	–	154.0
12′	6.86 (1H, *d,* 7.5)	113.7	6.86 (1H, *dd*, 8.1, 1.0)	113.3
13′	7.05 (1H, *t*, 8.0)	129.1	7.28 (1H, *t*, 8.1)	130.1
14′	6.70 (1H, *dd*, 8.0, 2.0)	121.6	6.69 (1H, *dd*, 8.1, 1.0)	121.6

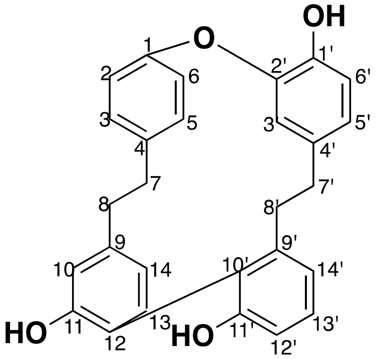

Isoriccadin C

Table S-4. ^{1}H NMR (500 MHz, δ_H, multi, (*J* in Hz) and ^{13}C NMR (125 MHz) data of compound **4** in acetone-d_6.

**No.**	**4**		**Marchantin K**	
	^{1}H-NMR (Acetone-d_6, 500 MHz)	^{13}C-NMR (Acetone-d_6, 125 MHz)	^{1}H-NMR ($CDCl_3$, 600 MHz)	^{13}C-NMR (CD_3OD, 150 MHz)
**1**	–	153.9	–	155.2
**2**	6.51 (1H, brd, *J* = 8.0 Hz)	123.4	6.50 (1H, brd, *J* = 8.0 Hz)	122.7
**3**	6.95 (1H, brd, *J* = 8.0 Hz)	129.4	6.91 (1H, brd, *J* = 8.0 Hz)	130.6
**4**	–	140.0	–	140.6
**5**	6.95 (1H, brd, *J* = 8.0 Hz)	129.4	6.91 (1H, brd, *J* = 8.0 Hz)	130.6
**6**	6.51 (1H, brd, *J* = 8.0 Hz)	121.4	6.50 (1H, brd, *J* = 8.0 Hz)	122.7
**7**	3.09–3.14 (1H, m)<br>2.95–3.06 (1H, m)	35.5	3.00–3.05 (1H, m)<br>2.90–2.97 (1H, m)	37.1
**8**	2.95–3.06 (2H, m)	30.1	2.90–2.97 (1H, m)<br>2.80 (1H, ddd, *J* = 15.0, 10.0, 2.0 Hz)	30.8

**9**	–	131.4	–	128.3
**10**	6.82 (1H, dd, *J* = 8.0, 1.5 Hz)	120.9	6.83 (1H, d, *J* = 8.5 Hz)	121.3
**11**	6.76 (1H, d, *J* = 8.5 Hz)	112.3	6.75 (1H, d, *J* = 8.5 Hz)	113.5
**12**	–	147.1	–	145.8
**13**	–	138.8	–	139.1
**14**	–	146.3	–	142.2
**1′**	–	136.8	–	134.8
**2′**	–	149.9	–	148.9
**3′**	4.97 (1H, d, *J* = 2.0 Hz)	107.6	4.96 (1H, d, *J* = 2.0 Hz)	109.2
**4′**	–	133.3	–	132.1
**5′**	6.59 (1H, d, *J* = 2.0 Hz)	106.0	6.52 (1H, d, *J* = 2.0 Hz)	107.0
**6′**	–	147.3	–	147.5
**7′**	4.08 (1H, dd, *J* = 9.5, 4.0 Hz)	84.1	4.06 (1H, dd, *J* = 10.0, 4.0 Hz)	85.9
**8′**	3.00 (1H, m)<br>2.59 (1H, dd, *J* = 13.0, 10.0 Hz)	43.8	3.03 (1H, dd, *J* = 12.0, 4.0 Hz)<br>2.56 (1H, dd, *J* = 12.0, 10.0 Hz)	45.0
**9′**	–	139.1	–	139.9
**10′**	6.68 (1H, d, *J* = 2.0 Hz)	116.9	6.68 (1H, d, *J* = 2.0 Hz)	118.1
**11′**	–	157.6	–	158.9
**12′**	6.45 (1H, dd, *J* = 8.5, 3.0 Hz)	114.4	6.46 (1H, ddd, *J* = 8.0, 3.0, 1.0)	114.0
**13′**	6.88 (1H, t, *J* = 7.5 Hz)	127.8	6.85 (1H, t, *J* = 8.0 Hz)	129.0
**14′**	6.05 (1H, brd, *J* = 7.0 Hz)	125.5	6.02 (1H, brd, *J* = 8.0 Hz)	124.6
**OCH_3**	3.20 (s)	–	3.26 (s)	–

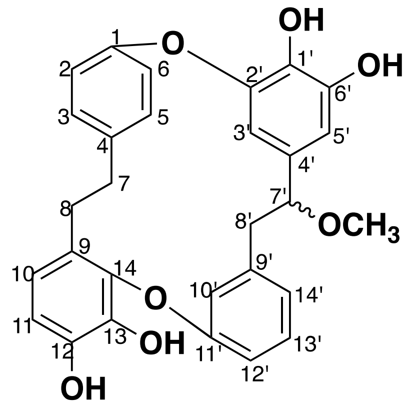

Marchantin K

Table S-5. ^{1}H NMR (500 MHz, δ_H, multi, (J in Hz) and ^{13}C NMR (125 MHz) data of compound **5** in acetone-d_6.

**No**	**5**		**Lunularin** (CD_3OD)	
	^{1}H-NMR (Acetone-d_6, 500 MHz)	^{13}C-NMR (Acetone-d_6, 125 MHz)	**δ_H, J (Hz)**	**δ_C**
1	–	155.5	–	156.5
2	6.73 (1H, *d*, 8.5)	115.0	6.67 (1H, *d*, 8.0)	116.1
3	7.03 (1H, *d*, 8.0)	129.1	6.96 (1H, *d*, 8.0)	130.5
4	–	132.6	–	134.1
5	7.03 (1H, *d*, 8.0)	129.1	6.96 (1H, *d*, 8.0)	130.5
6	6.73 (1H, *d*, 8.5)	115.0	6.67 (1H, *d*, 8.0)	116.1
7	2.78 (2H, *s*)	36.8	2.76 (2H, *s*)	38.3
8	2.78 (2H, *s*)	38.1	2.76 (2H, *s*)	39.6
1′	–	143.6	–	144.9
2′	6.67 (1H, *s*)	115.4	6.60 (1H, *s*)	116.5
3′	–	157.4	–	158.3
4′	6.69 (1H, *d*, 2.0)	112.7	6.61 (1H, *d*, 7.8)	113.8

5′	7.07 (1H, *t*, 7.8)	129.3	7.03 (1H, *t*, 7.8)	131.3
6′	6.63 (1H, *d*, 8.0)	119.5	6.64 (1H, *m*)	121.0

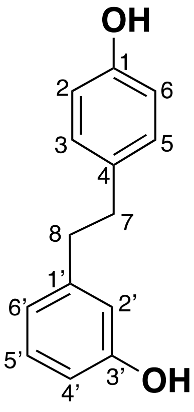

Lunularin

Table S-6. ^{1}H NMR (500 MHz, δ_H, multi, (J in Hz) and ^{13}C NMR (125 MHz) data of

No.	**6**		**3-(3,4-Dimethoxybenzyl)-5,7-dimethoxyphthalide**	
	δ_H (Acetone-d_6, 500 MHz)	δ_C (Acetone-d_6, 125 MHz)	δ_H (multi, J in Hz) (CDCl$_3$, 400 MHz)	δ_C (CDCl$_3$, 100 MHz)
1	/	167.1	/	168.0
3	5.85 (t, 6.0)	80.3	5.59 (t, 6.1)	79.9
4	6.61 (s)	99.5	6.21 (d, 1.2)	98.3
5	/	166.8	/	166.5
6	6.54 (d, 2.0)	99.6	6.38 (d, 1.7)	98.9
7	/	160.3	/	159.7
8	/	105.1	/	107.3
9	/	154.8	/	154.2
10	3.25 (dd, 14.0, 5.0)<br>3.10 (dd, 14.0, 6.0)	40.6	3.16 (dd, 14.2, 6.3)<br>3.07 (dd, 14.2,5.9)	40.5
1′	/	129.3	/	127.8
2′	6.87 (d, 1.5)	114.6	6.73 (br, s)	113.3
3′	/	150.0	/	149.0
4′	/	148.3	/	148.3
5′	6.82 (d, 8.0)	112.6	6.78 (d, 8.0)	111.5
6′	6.77 (dd, 8.5, 2.0)	122.9	6.74 (dd, 7.3, 1.8)	122.1
5-OCH$_3$	3.87 (s)	56.4	3.79 (s)	56.0
7-OCH$_3$	3.89 (s)	56.0	3.91 (s)	56.0
3′-OCH$_3$	3.75 (s)	56.3	3.83 (s)	55.9
4′-OCH$_3$	3.73 (s)	56.4	3.84 (s)	55.9

compound **6** in acetone-d_6.

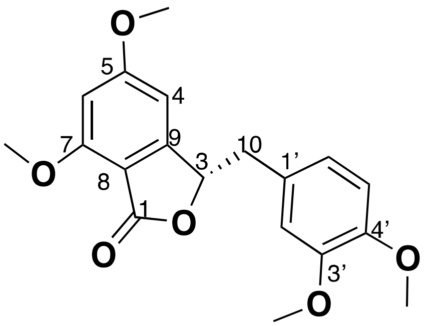

3-(3,4-Dimethoxybenzyl)-5,7-dimethoxyphthalide

Table S-7. ^{1}H NMR (500 MHz, δ_H, multi, (J in Hz) and ^{13}C NMR (125 MHz) data of compound **8** in acetone-d_6.

No.	**8**			(5*S*,8*R*,9*S*,10*R*)-2-Oxo-ent-3-cleroden-15-oic acid (A)		Methyl (5*S*,8*R*,9*S*,10*R*)-2-oxo-ent-clerod-3,13-dien-15-oate (B)	
	δ_H (multi, J in Hz) (Acetone-d_6, 500MHz)	δ_C (Acetone-d_6, 500 MHz)	δ_H (multi, J in Hz) ($CDCl_3$, 200 MHz)	δ_H (multi, J in Hz) ($CDCl_3$, 200 MHz)	δ_C ($CDCl_3$, 50 MHz)	δ_H (multi, J in Hz) ($CDCl_3$, 200 MHz)	δ_C ($CDCl_3$, 50 MHz)
1	2.66 (dd, 18.5, 7.0)<br>2.43 (d, 18.0)	35.7	2.69 (dd, 18.5, 6.5)<br>2.50 (d, 18.5)		35.1		35.4
2		198.8			199.1		200.3
3	5.56 (d, 1.0)	129.1	5.84 (brs)	5.70 (m)	128.5	5.65 (br s)	128.5
4		169.4			168.6		167.5
5		40.1			38.6		38.6
6		37.5			36.7		36.7
7		29.1			28.9		28.9

8		37.4			36.6		36.6
9		40.3			39.3		39.3
10	1.90 (m)	47.9	1.83 (d, 6.5)		45.7		45.7
11		31.3			35.4		34.0
12		35.8			36.2		36.8
13		72.8			30.7		160.3
14	5.93 (dd, 17.0, 10.5)	147.1	5.87 (dd, 10.5, 17.5)	5.70 (m)	41.4		115.2
15	5.21 (dd, 17.5, 2.0)<br>4.98 (dd, 11.0, 2.0)	111.5	5.09 (d, 11.0)<br>5.20 (d, 16.5)	7.65 (1H, br s)	178.7		167.0
16	1.24 (s)	28.5	1.22 (s)	2.17 (s)<br>1.10 - 0.8 (m)	19.9	2.09 (br s)<br>0.96 (d, 6.0)	19.1
17	0.78 (d, 7.0)	16.3	0.77 (d, 7.0)	1.10 - 0.8 (m)	16.0	0.80 - 0.50 (m)	15.9
18	1.96 (d, 1.0)	20.5	1.94 (d, 1.5)	1.90 (br s)	20.5	1.80 (br s)	20.5
19	1.25 (s)	32.3	1.25 (s)	1.10 - 0.8 (m)	32.1	1.06 (br s)	32.1
20	0.56 (s)	19.6	0.58 (s)		18.0	0.80 - 0.50 (m)	17.8

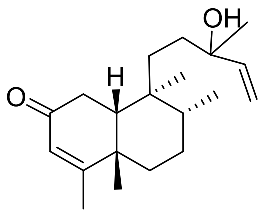

Marchanol (8)

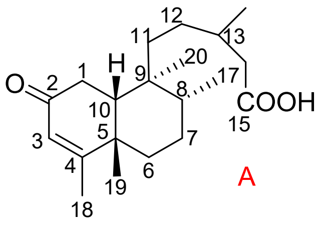

(5*S*,8*R*,9*S*,10*R*)-2-Oxo-*ent*-3-cleroden-15-oic acid

44-Molecules2009
2a-lopes1987

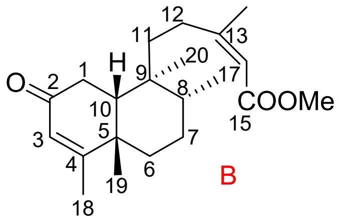

Methyl (5*S*,8*R*,9*S*,10*R*)-2-oxo-*ent*-clerod-3,13-dien-15-oate

49-molecules2009
2b-lopes1987

1. A.G. Pacheco, P. Machado De Oliveira, D. Piló-Veloso, A. Flávio De Carvalho Alcântara. 13C-NMR Data of Diterpenes Isolated from Aristolochia Species. *Molecules* **2009**, 14 (3), 1245–1262.
2. M. X. Lopes, L. M. V. Trevisan, and V. da S. Bolzani, *Clerodane diterpenes from Aristolochia species. Phytochemistry,* 1987, **26**, 2781-2784, DOI: 10.1016/S0031-9422(00)83590-6

Table S-8. ^{1}H NMR (500 MHz, δ_H, multi, (J in Hz) and ^{13}C NMR (125 MHz) data of compound **9** in acetone-d_6.

**No.**	**9**		**Vitexilactone**	
	δ_H (multi, J in Hz) (Acetone-d_6, 500 MHz)	δ_C (Acetone-d_6, 125 MHz)	δ_H (multi, J in Hz) (CDCl$_3$, 500 MHz)	δ_C (CDCl$_3$, 125 MHz)
1	1.43 (m)<br>1.32 (m)	32.5	1.45 (m)<br>1.36 (ca)	33.6
2	1.71 (m)<br>1.50 (m)	19.6	1.65 (m)<br>1.50 (ca)	18.6
3	1.37 (m)<br>1.24 (m)	43.0	1.36 (ca)<br>1.17 (ddd, 3.0, 13.5, 13.5)	43.6
4	–	34.7	–	34.0
5	1.76 (d, 2.3)	45.6	1.56 (d, 3.0)	47.7
6	5.34 (q, 3.0, 3.0, 2.5)	70.4	5.39 (ddd 3.0, 3.0, 3.0)	69.8

7	1.47 (m)<br>1.44 (m)	37.4	1.59 (ca)<br>1.50 (ca)	36.1
8	2.17 (m)	32.8	2.14 (m)	32.1
9	2.02	77.0	–	76.5
10	–	44.7	–	43.8
11	1.85 (q, 6.5, 3.0, 4.5)<br>1.82 (q, 4.0, 4.0, 6.5)	32.6	1.98 (ddd, 6.0, 10.5, 15.0)<br>1.75 (ddd, 6.0, 10.5, 15.0)	31.6
12	2.61 (m) 2H	26.0	2.50 (ca) 2H	25.4
13	–	173.4	–	171.1
14	5.86 (p, 1.5, 2.0, 1.5, 2.0)	114.8	5.84 (dddd, 1.5, 1.5, 1.5 1.5)	115.0
15	–	174.0	–	174.0
16	4.85 (d) 2H	73.8	4.76 (br s) 2H	73.2
17	0.92 (s)	16.4	0.90 (d, 6.5)	16.1
18	0.93 (s)	34.0	0.97 (s)	33.6
19	1.02 (s)	24.1	1.01 (s)	23.7
20	1.30 (s)	19.5	1.26 (s)	19.0
1’	–	170.5	–	170.4
2’	2.00 (s)	21.8	2.06 (s)	21.9

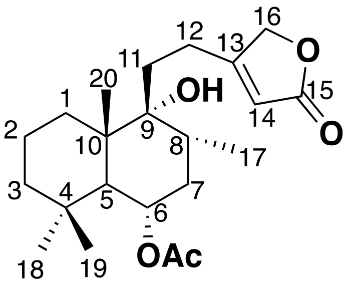

Vitexilactone

Table S-9. ^{1}H NMR (500 MHz, δ_H, multi, (*J* in Hz) in acetone-d_6, $CDCl_3$ and ^{13}C NMR (125 MHz) data of compound **10** in acetone-d_6.

No.	10			12-oleanene-3-one	
	δ_C (Acetone-*d*$_6$, 125 MHz)	δ_H (Acetone-*d*$_6$, 500 MHz)	δ_H ($CDCl_3$, 500 MHz)	δ_C ($CDCl_3$, 125 MHz)	δ_H ($CDCl_3$, 500 MHz)
1	39.8	–	–	39.8	–
2	34.7	–	–	34.5	–
3	216.2	–	–	217.2	–
4	47.7	–	–	47.6	–
5	56.0	–	–	55.3	–
6	20.4	–	–	18.8	–
7	33.1	–	–	33.2	–
8	40.0	–	–	40.1	–
9	47.8	2.31 (1H, m)	2.37 (1H, m)	47.7	2.33 (1H, m)
10	35.5	–	–	36.6	–
11	23.7	–	–	23.7	–
12	122.8	5.24 (1H, t, 4.0)	5.21 (1H, t, 3.5, 4.0)	122.4	5.27 (1H, dd, 3.7, 3.4)
13	146.0	–	–	144.1	–
14	41.9	–	–	42.5	–
15	26.4	–	–	26.2	–
16	26.9	–	–	26.9	–
17	32.3	–	–	32.5	–
18	47.0	–	–	47.2	–
19	47.3	–	–	47.1	–
20	31.3	–	–	31.0	–
21	34.7	–	–	34.6	–

22	37.1	–	–	36.9	–
23	26.4	1.03 (3H, s)	1.06 (3H, s)	26.4	1.04 (3H, s)
24	21.9	1.05 (3H, s)	1.07 (3H, s)	22.1	1.07 (3H, s)
25	15.6	1.10 (3H, s)	1.02 (3H, s)	15.6	1.01 (3H, s)
26	17.3	1.06 (3H, s)	1.00 (3H, s)	16.8	0.99 (3H, s)
27	24.6	1.19 (3H, s)	1.25 (3H, s)	25.9	1.24 (3H, s)
28	28.9	0.87 (3H, s)	0.84 (3H, s)	28.6	0.84 (3H, s)
29	23.7	0.89 (3H, s)	0.92 (3H, s)	23.7	0.90 (3H, s)
30	32.3	0.88 (3H, s)	0.87 (3H, s)	32.8	0.86 (3H, s)

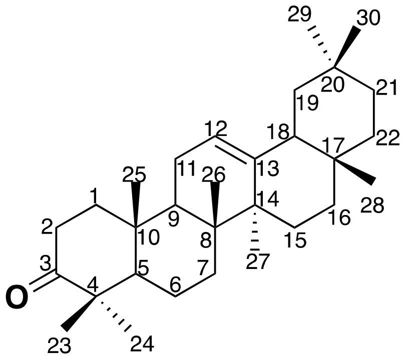

12-oleanene-3-one

Table S-10. ^{1}H NMR (400 MHz, δ_H, multi, (J in Hz) and ^{13}C NMR (125 MHz) data of compound **11** in $CDCl_3$

No.	**11**		**3,11-dioxo ursolic acid**	
	δ_H (multi, J in Hz) ($CDCl_3$, 400 MHz)	δ_C ($CDCl_3$, 100 MHz)	δ_H (multi, J in Hz) ($CDCl_3$, 300 MHz)	δ_C ($CDCl_3$, 100.6 MHz)
1	38.7	–	39.9	–
2	34.3	–	33.5	–
3	217.4	–	215.9	–
4	47.9	–	46.7	–

5	55.5	–	54.0	–
6	19.0	–	18.3	–
7	32.5	–	32.0	–
8	39.9	–	40.7	–
9	60.8	2.39 (1H, s)	59.7	2.48 (1H, s)
10	36.9	–	37.5	–
11	199.5	–	198.7	–
12	130.8	5.63 (1H, s)	131.8	5.61 (1H, s)
13	163.3	–	164.8	–
14	44.0	–	43.6	–
15	28.6	–	28.4	–
16	23.8	–	24.2	–
17	47.6	–	47.0	–
18	52.6	–	53.8	2.46 (1H, d, J=12)
19	41.5	–	71.9	–
20	41.5	–	41.1	–
21	26.6	–	25.6	–
22	36.1	–	36.4	–
23	26.6	0.90 (3H, s)	26.1	0.88 (3H, s)
24	21.2	0.95 (3H, s)	20.8	0.94 (3H, s)
25	15.7	1.02 (3H, s)	15.3	0.98 (3H, s)
26	18.8	1.24 (3H, s)	18.2	1.22 (3H, s)
27	21.1	1.31 (3H, s)	20.7	1.30 (3H, s)
28	182.8		180.0	
29	23.7	0.87 (3H, d, J= 6.5 Hz)	26.0	0.85 (3H, d, J= 7.5 Hz)
30	17.2	0.97(3H, d, J= 6.3 Hz)	16.0	0.90 (3H, d, J= 8.0 Hz)

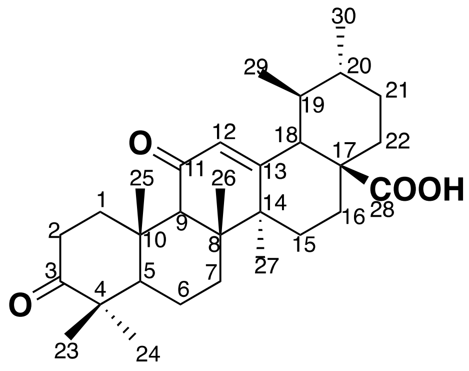

3,11-dioxo ursolic acid

Table S-11. ^{1}H NMR (500 MHz, δ_H, multi, (*J* in Hz) and ^{13}C NMR (125 MHz) data of compound **12** in $CDCl_3$

**No.**	**12**		**Ursolic acid**	
	δC (CDCl$_3$, 125 MHz)	δH (CDCl$_3$, 500 MHz)	δC	δH
1	38.9	–	38.9	–
2	27.4	–	27.1	–
3	79.2	3.22 (1H, dd, J= 5.0, 7.0, 4.0)	79.0	3.11 (1H, dd, J=9.0, 7.0)
4	39.0	–	39.0	–
5	55.4	–	55.6	–
6	18.5	–	18.6	–
7	33.2	–	33.4	–
8	39.6	–	39.8	–
9	47.7	–	47.9	–
10	36.8	–	37.2	–
11	23.7	–	23.6	–
12	126.0	5.26 (1H, t, J=4.0)	125.8	5.16 (1H, t, J=3.5)

13	138.1	–	138.5	–
14	42.2	–	42.4	–
15	30.8	–	29.9	–
16	24.4	–	24.5	–
17	48.0	–	48.1	–
18	53.0	2.19 (1H, d, J=11.5)	53.2	2.11 (1H, d, J=11.8)
19	41.7	–	39.4	–
20	39.6	–	39.2	–
21	26.6	–	30.9	–
22	36.1	–	37.1	–
23	28.3	0.92 (3H, s)	28.3	0.90 (3H, s)
24	15.6	0.77 (3H, s)	15.6	0.74 (3H, s)
25	15.8	0.78 (3H, s)	15.8	0.70 (3H, s)
26	17.2	0.85 (3H, s)	17.2	0.84 (3H, s)
27	21.1	0.99 (3H, s)	23.7	1.01 (3H, s)
28	182.8	–	180.8	–
29	17.1	0.86 (3H, d, J= 6.5)	17.1	0.86 (3H, d, J= 6.1)
30	21.3	0.77 (3H, d, J= 4)	21.3	0.78 (3H, d, J= 6.5)

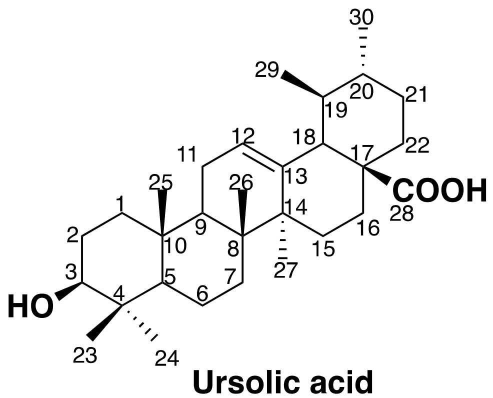

Ursolic acid

Table S-12. ^{1}H NMR (500 MHz, δ_H, multi, (*J* in Hz) in acetone-d_6, $CDCl_3$ and ^{13}C NMR (125 MHz) data of compound **13** in acetone-d_6.

**No.**	**13**		**Artemetin**	
	^{1}H-NMR (Acetone-d_6, 500 MHz)	^{13}C-NMR (Acetone-d_6, 125 MHz)	^{1}H-NMR (DMSO-d_6, 500 MHz)	^{13}C-NMR (DMSO-d_6, 125 MHz)
**1**	–	–	–	–
**2**	–	155.8	–	155.6
**3**	–	138.6	–	138.1
**4**	–	178.9	–	178.4
**4a**	–	106.2	–	105.7
**5**	–	152.7	–	151.9
**6**	–	132.3	–	131.7
**7**	–	159.3	–	158.8
**8**	6.81 (1H, s)	90.8	6.89 (1H, s)	91.6
**8a**	–	152.3	–	151.7
**1′**	–	122.8	–	122.2
**2′**	7.78 (1H, d, *J* = 2.0 Hz)	122.1	7.61 (1H, d, *J* = 2.0 Hz)	124.0
**3′**	–	149.2	–	148.7
**4′**	–	152.0	–	151.4
**5′**	7.14 (1H, d, *J* = 8.5 Hz)	111.3	7.11 (1H, d, *J* = 8.5 Hz)	111.3
**6′**	7.76 (1H, dd, *J* = 2.0, 3.0 Hz)	111.8	7.69 (1H, dd, *J* = 2.0, 8.5 Hz)	111.7
**3-OCH₃**	3.92 (3H, s)	59.4	3.77 (3H, s)	56.6
**5-OH**	12.69 (1H, s)	–	12.53 (1H, s)	–

**6-OCH_3**	3.96 (3H, s)	55.9	3.88 (3H, s)	55.8
**7-OCH_3**	3.90 (6H, s)	59.7	3.82 (3H, s)	60.1
**3′-OCH_3**		55.4	3.81 (3H, s)	55.8
**4′-OCH_3**	3.80 (3H, s)	55.3	3.68 (3H, s)	55.7

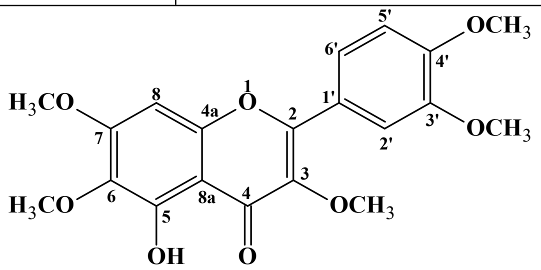

Armetin

Table S-13. ^{1}H NMR (500 MHz, δ_H, multi, (J in Hz) in acetone-d_6, $CDCl_3$ and ^{13}C NMR (125 MHz) data of compound **1a** and **1** in acetone-d_6.

**No**	**1a**		**1**	
	^{1}H-NMR (acetone-d_6, 500 MHz)	^{13}C-NMR ($CDCl_3$, 125 MHz)	^{1}H-NMR (acetone-d_6, 500 MHz)	^{13}C-NMR ($CDCl_3$, 125 MHz)
1	–	154.1	–	153.2
2	6.50 (1H, *d*, 7.5)	121.8	6.57 (1H, *d*, 8.0)	121.2
3	6.96 (1H, *d*, 8.0)	129.1	6.91 (1H, *d*, 8.5)	129.6
4	–	137.1	–	139.1
5	6.96 (1H, *d*, 8.0)	129.1	6.91 (1H, *d*, 8.5)	129.6
6	6.50 (1H, *d*, 7.5)	121.8	6.57 (1H, *d*, 8.0)	121.2
7	3.02 – 3.06 (4H, *m*)	35.7	2.97–3.01 (2H, *m*)	35.3
8	3.02 – 3.06 (4H, *m*)	34.1	2.97–3.01 (2H, *m*)	30.3
9	–	130.7	–	136.2
10	6.82 (1H, *dd*, 8.0, 1.5)	121.5	7.00 (1H, *dd*, 8.0, 1.5)	121.9

11	7.00 (1H, *t*, 8.0)	122.1	7.13 (1H, *dd*, 8.0, 7.5)	126.0
12	6.61 (1H, *dd*, 8.5, 2.5)	113.8	6.85 (1H, *dd*, 8.0, 1.5)	114.4
13	–	118.5	–	148.7
14	–	142.4	–	139.7
1′	–	139.5	–	130.8
2′	–	147.0	–	146.5
3′	5.34 (1H, *s*)	109.6	5.13 (1H, *d*, 1.5)	107.9
4′	–	133.8	–	132.5
5′	–	105.0	6.46 (1H, *d*, 1.5)	109.3
6′	–	144.5	–	144.3
7′	2.91 – 2.93 (2H, *m*)	34.8	2.78–2.80 (2H, *m*)	34.1
8′	2.91 – 2.93 (2H, *m*)	35.7	2.72–2.74 (2H, *m*)	35.5
9′	–	142.4	–	143.1
10′	6.28 (1H, *d*, 7.5)	115.2	6.57 (1H, *dd*, 2.5, 2.0)	115.5
11′	–	158.7	–	156.8
12′	7.04 (1H, *dd*, 8.0, 1.5)	116.5	6.53 (1H, *dd*, 8.5, 2.0)	112.0
13′	7.10 (1H, *t*, 7.8)	130.5	6.97 (1H, *t*, 7.8)	128.9
14′	6.28 (1H, *d*, 7.5)	126.4	6.39 (1H, *brd*, 7.5)	123.2

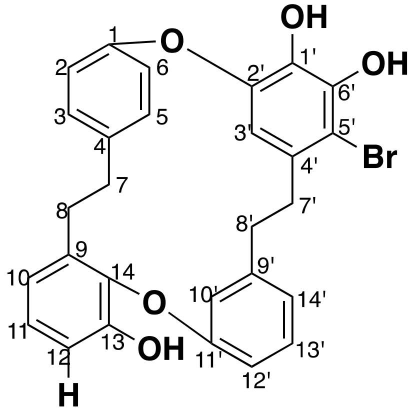

1a

Figure S-1. Compounds reported in *Marchantia polymorpha* L.

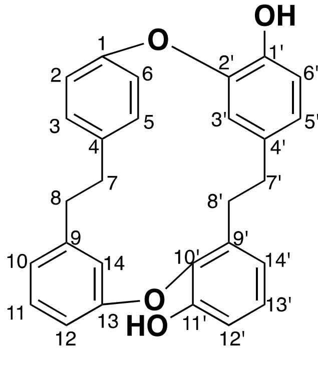

Isomarchantin C **(1)**

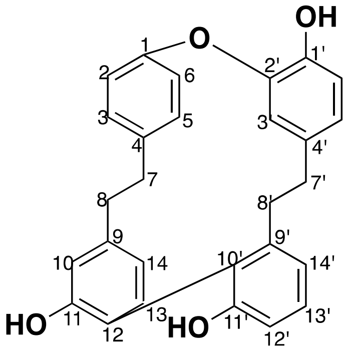

Isoriccadin C **(2)**

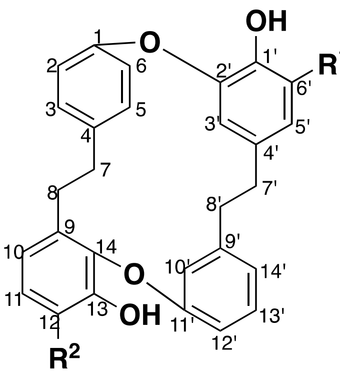

R^1=OH R^2=H Marchantin A **(3)**
R^1=H R^2=H Marchantin C **(4)**

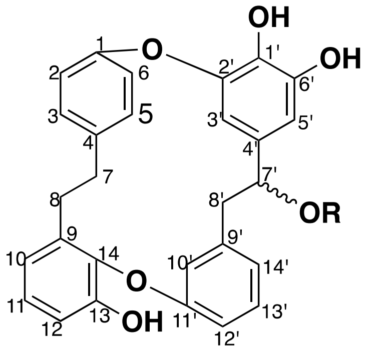

R=H Marchantin D **(5)**
R=CH_3 Marchantin E **(6)**

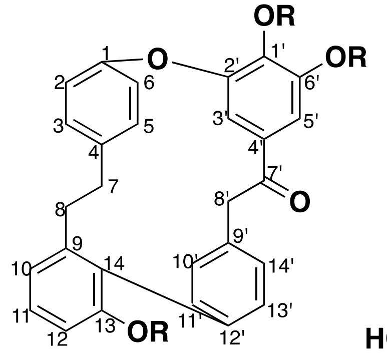

R=H Marchantin G **(7)**
R=CH_3 Riccardin C **(8)**

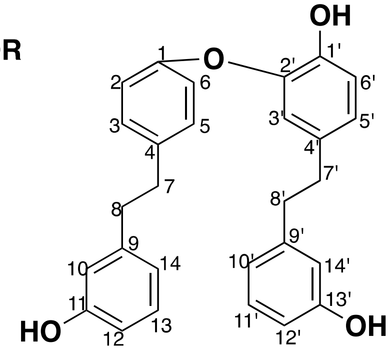

Perrottetin **(9)**

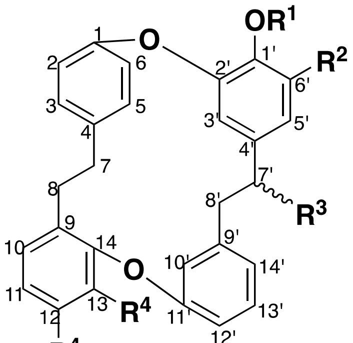

R^1=H R^2=H R^3=H R^4=OH Marchantin B **(15)**
R^1=H R^2=OH R^3=OCH_3 R^4=OH Marchantin K **(17)**

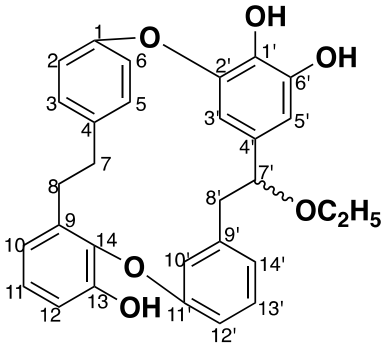

Marchantin J **(16)**

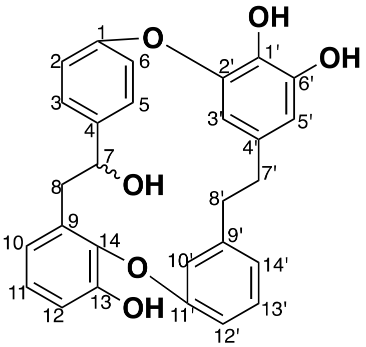

Marchantin L **(18)**

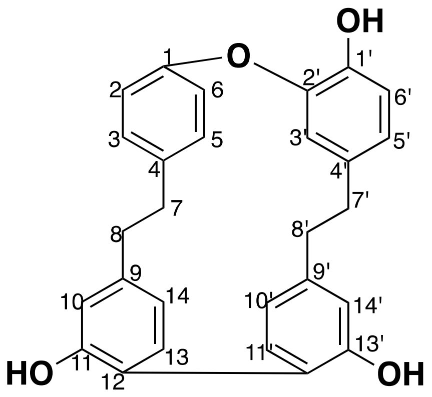

Polymorphatin A **(29)**

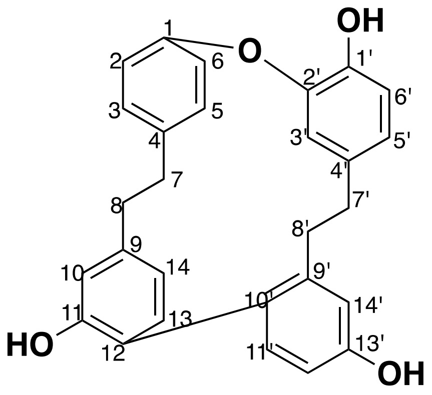

Isorricardin D **(30)**

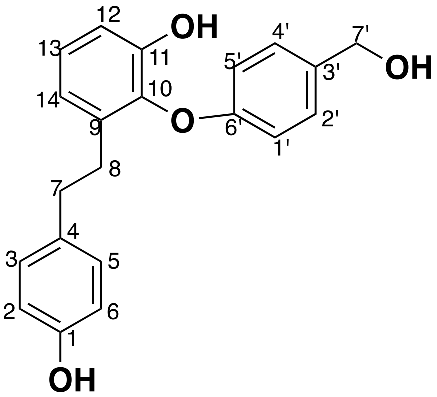

2-[3-(hydroxymethyl)phenoxy]-3-[(4-hydroxyphenyl)ethyl]phenol **(31)**

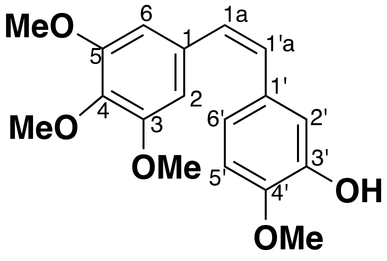

Combrerastatin A **(32)**

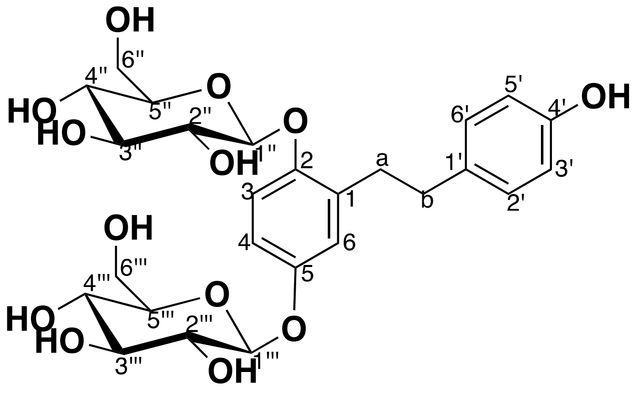

α,β-dihydrostilbene-2,4',5-triol-2,5-di(β-D-glucopyranoside) **(49)**

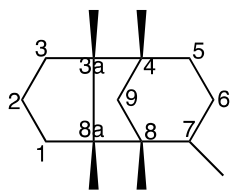

(-)-Gymnomitrene **(19)**

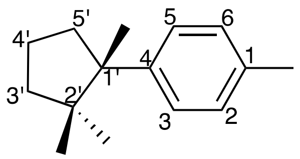

(-)-Parene **(20)**

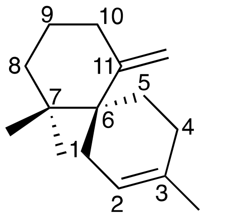

(+)-β-Chamigrene **(21)**

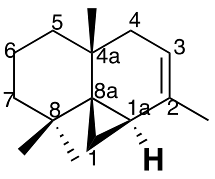

(+)-Thujopsene **(22)**

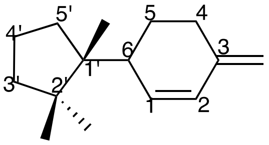

(-)-δ-Cuprenene **(23)**

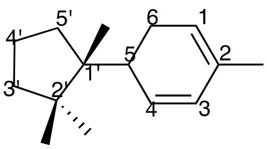

(+)-ε-Cuprenene **(24)**

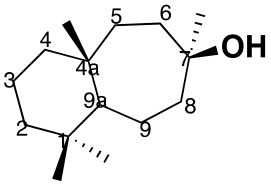

(-)-Widdrol **(25)**

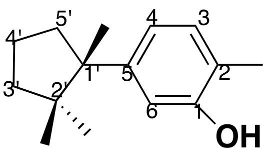

(-)-δ-Cuparenol **(26)**

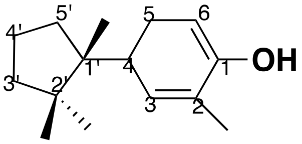

(-)-β-Herbertenol **(27)**

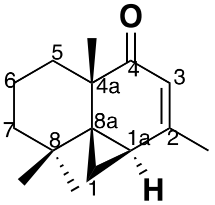

(-)-Thujopsenone **(28)**

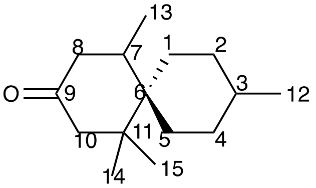

Ent-9-oxo-α-chamigrene **(10)**

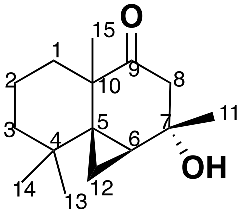

Ent-thujopsan-7β-ol **(11)**

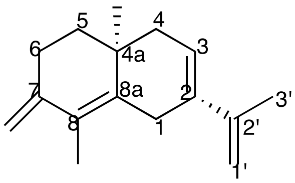

Ent-α-cyperone **(12)**

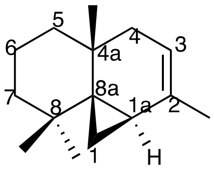

Ent-11-thujopsenone **(13)**

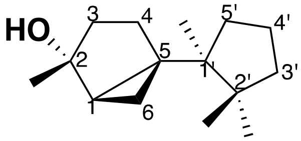

(-)-Cyclopropane cuparenol **(14)**

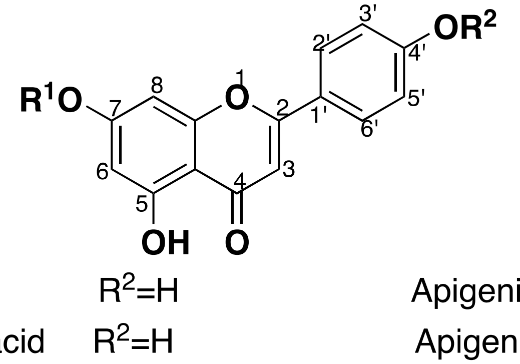

R^1=H R^2=H Apigenin **(33)**

R^1=glucuronic acid R^2=H Apigenin 7-glucuronide **(34)**

R^1=glucuronic acid R^2=glucuronic acid Apigenin 7,4'-di-O-β-D-glucuronide **(35)**

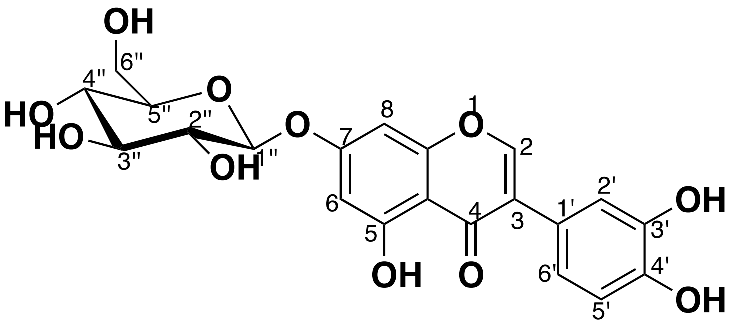

3',4',5,7-tetrahydroxyisoflavone 7-(β-D-glucopyranoside) **(55)**

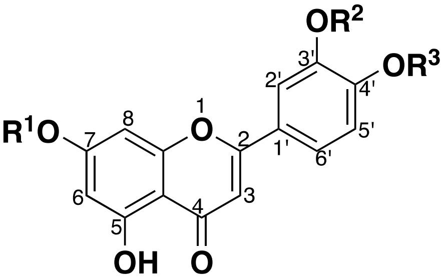

R^1=H	R^2=H	R^3=H	Luteolin **(36)**
R^1=glucuronic acid	R^2=H	R^3=H	Luteolin 7-glucuronide **(37)**
R^1=H	R^2=glucuronic acid	R^3=H	Luteolin 3'-O-β-D-glucuronide **(38)**
R^1=glucuronic acid	R^2=glucuronic acid	R^3=H	Luteolin 7,3'-diglucuronide **(39)**
R^1=glucuronic acid	R^2=H	R^3=glucuronic acid	Luteolin 7,4'-diglucuronide **(40)**
R^1=H	R^2=glucuronic acid	R^3=glucuronic acid	Luteolin 3',4'-O-β-D-diglucuronide **(41)**
R^1=glucuronic acid	R^2=glucuronic acid	R^3=glucuronic acid	Luteolin 7,3',4'-tri-O-β-D-glucuronide **(42)**

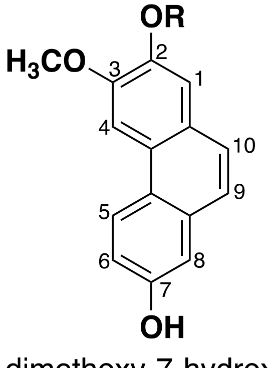

R=OCH_3 2,3-dimethoxy-7-hydroxyphenanthrene **(43)**
R=H 2,7-dimethoxy-3-hydroxyphenanthrene **(44)**

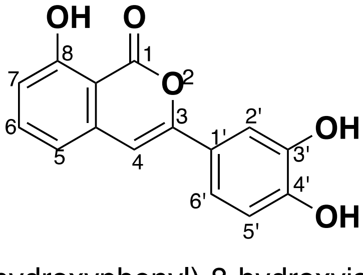

3-(3,4-dihydroxyphenyl)-8-hydroxyisocoumarin **(48)**

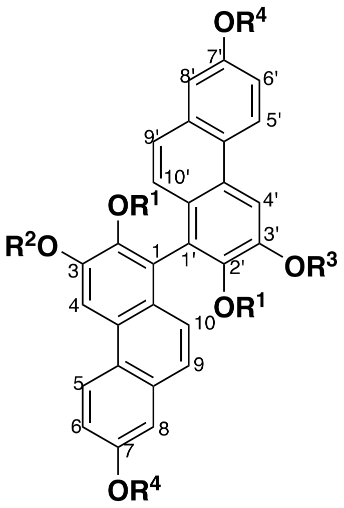

$R^1 = R^4 = H$, $R^2 = R^3 = OCH_3$ 3,3¢-dimethoxy-2,2¢,7,7¢-tetrahydroxy-1,1¢-biphenanthrene **(45)**
$R^1 = R^3 = R^4 = H$, R2= OCH_3 3-methoxy-2,2¢,3¢,7,7¢-pentahydroxy-1,1¢-biphenanthrene **(46)**
$R^1 = R^3 = R^3 = R^4 = OH$ 2,2¢,3,3¢,7,7¢-hexahydroxy-1,1¢-biphenanthrene **(47)**

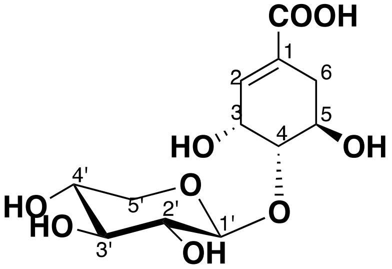

Shikimic acid 4-*β*-D-xylopyranoside **(50)**

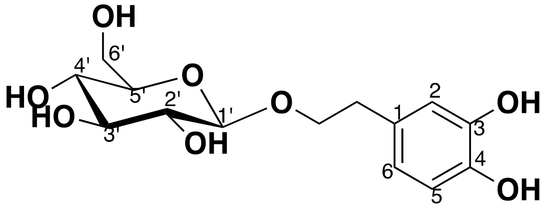

2-(3,4-dihydroxyphenyl)ethyl-*β*-D-glucopyranoside **(54)**

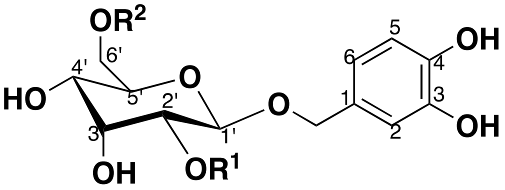

R^1=*α*-Rah*p*	R^2=H	2-(3,4-dihydroxyphenyl)ethyl-O-*α*-L -rhamnopyranosyl-(1→2)-*β*-D-allopyranoside **(51)**
R^1=H	R^2=*β*-Xyl*p*	2(3,4-dihydroxyphenyl)ethyl-O-*β*-D -xylopyranosyl-(1→6)-*β*-D-allopyranoside **(52)**
R^1=H	R^2=H	2-(3,4-dihydroxyphenyl)ethyl-*β*-D-allopyranoside **(53)**

Figure S-2. Compounds reported in Vietnamese *Marchantia polymorpha* L.

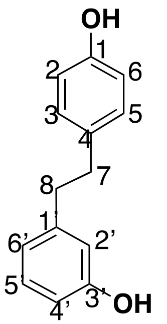

Lunularin (**N1**)

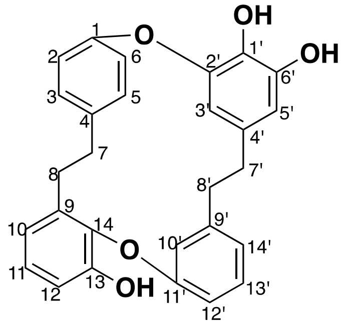

Marchantin A (**N2**)

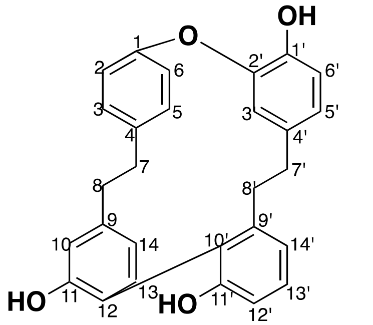

Isoriccadin C (**N3**)

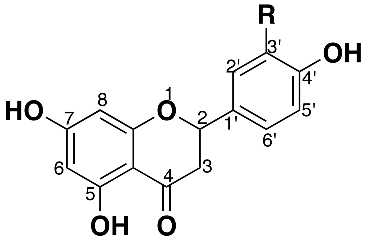

R = OH Luteolin (**N4**)
R = H Apigenin (**N5**)

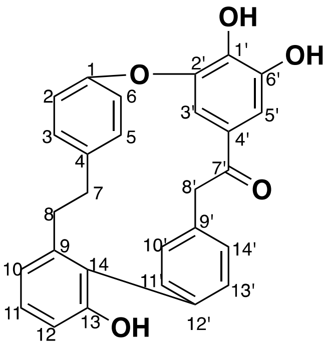

Marchantin G (**N6**)

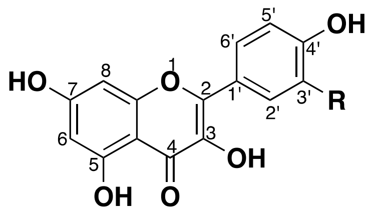

R = OH Quercetin (**N7**)
R = H Kaempferol (**N8**)

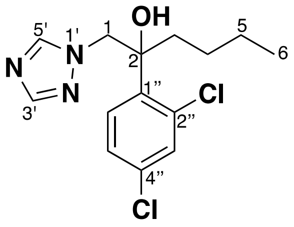

Hexaconazole (**N9**)

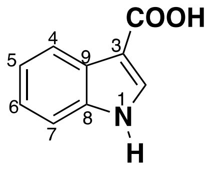

Indole-3-carboxylic acid (**N10**)

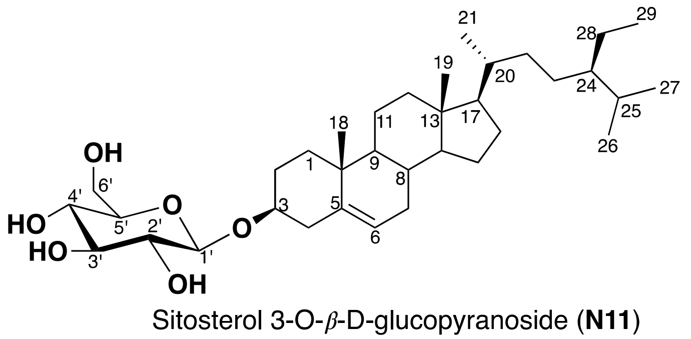

Sitosterol 3-O-β-D-glucopyranoside (**N11**)

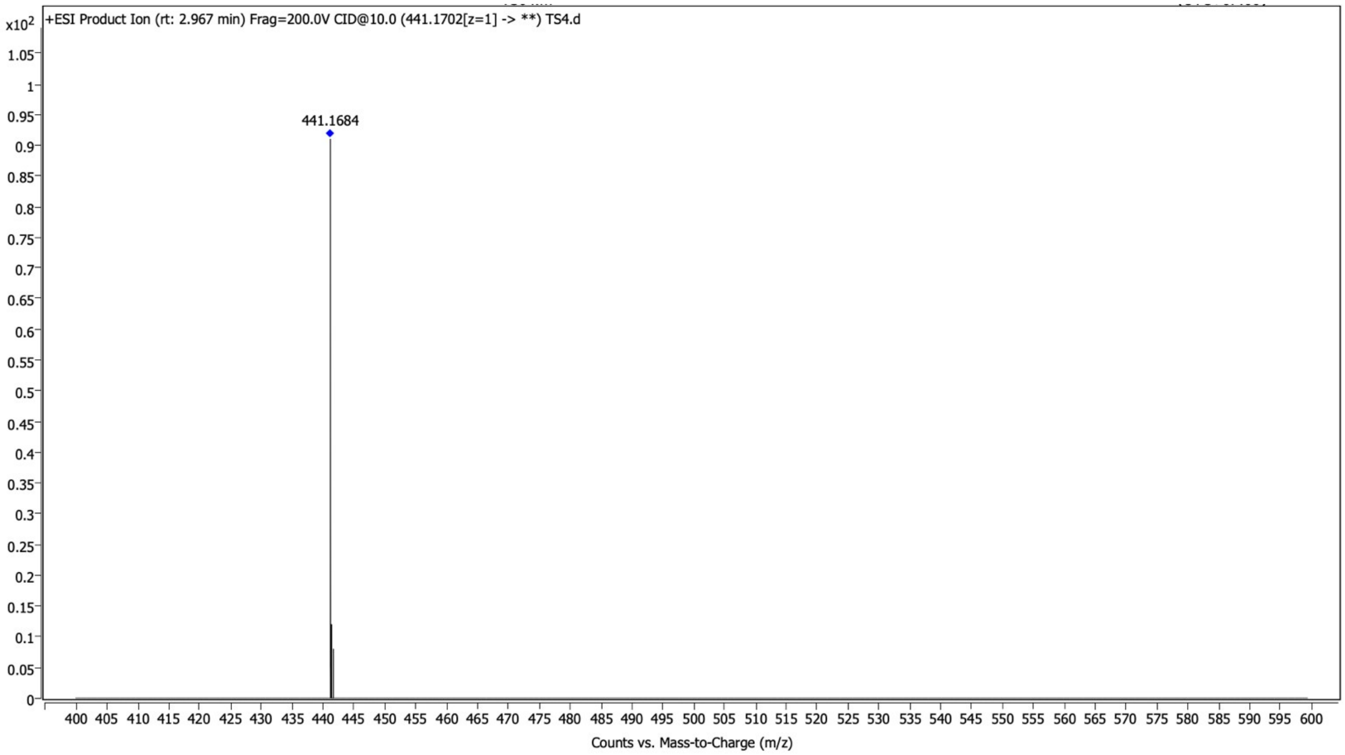

Figure S-3A. The HRESIMS spectrum of **1**.

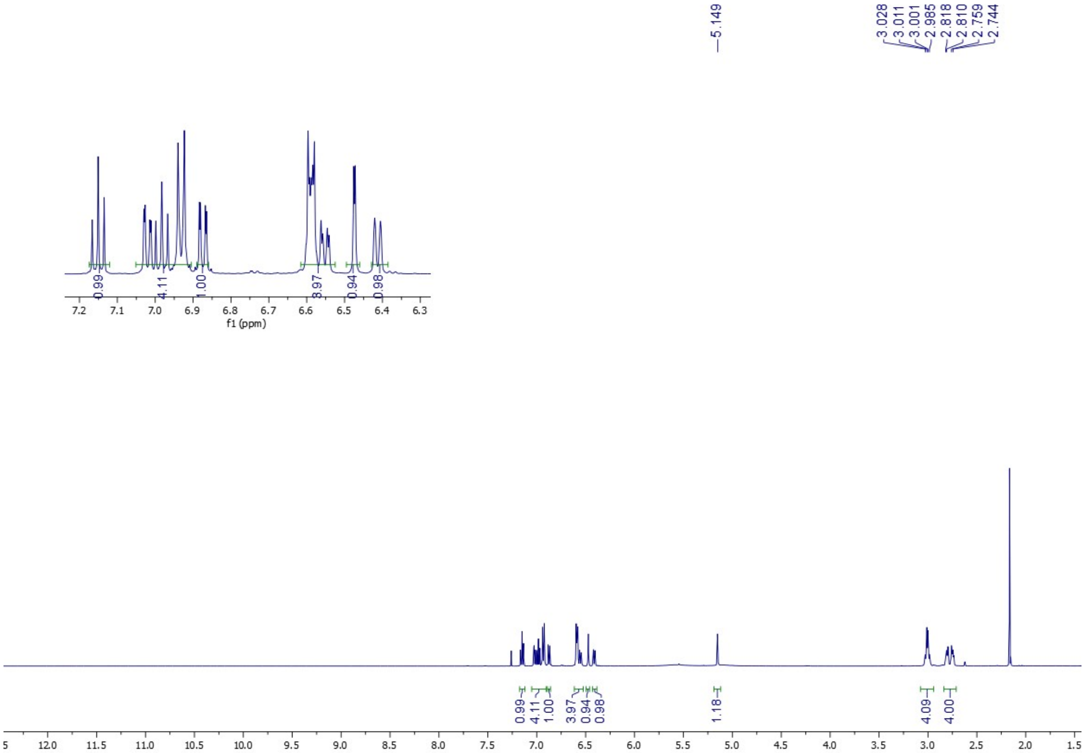

Figure S-3B. The ^{1}H NMR spectrum of **1** in acetone-d_6

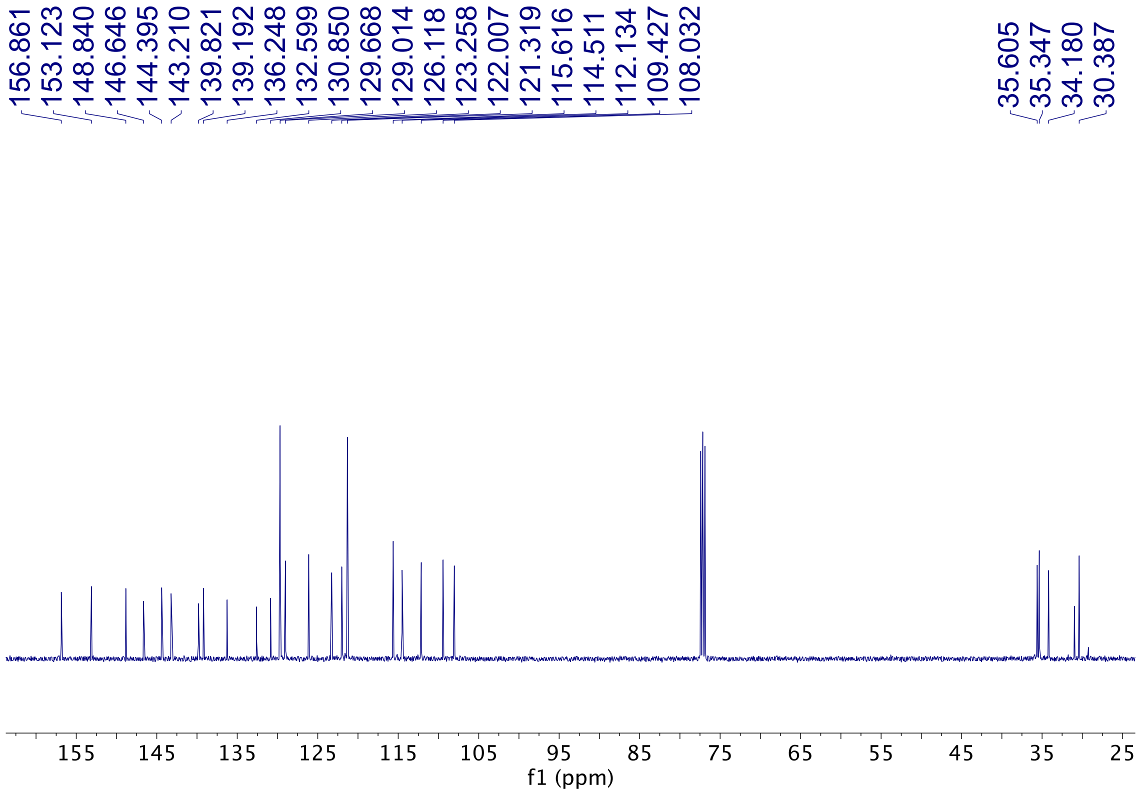

Figure S-3C. The ^{13}C-NMR spectrum of 1 in $CDCl_3$.

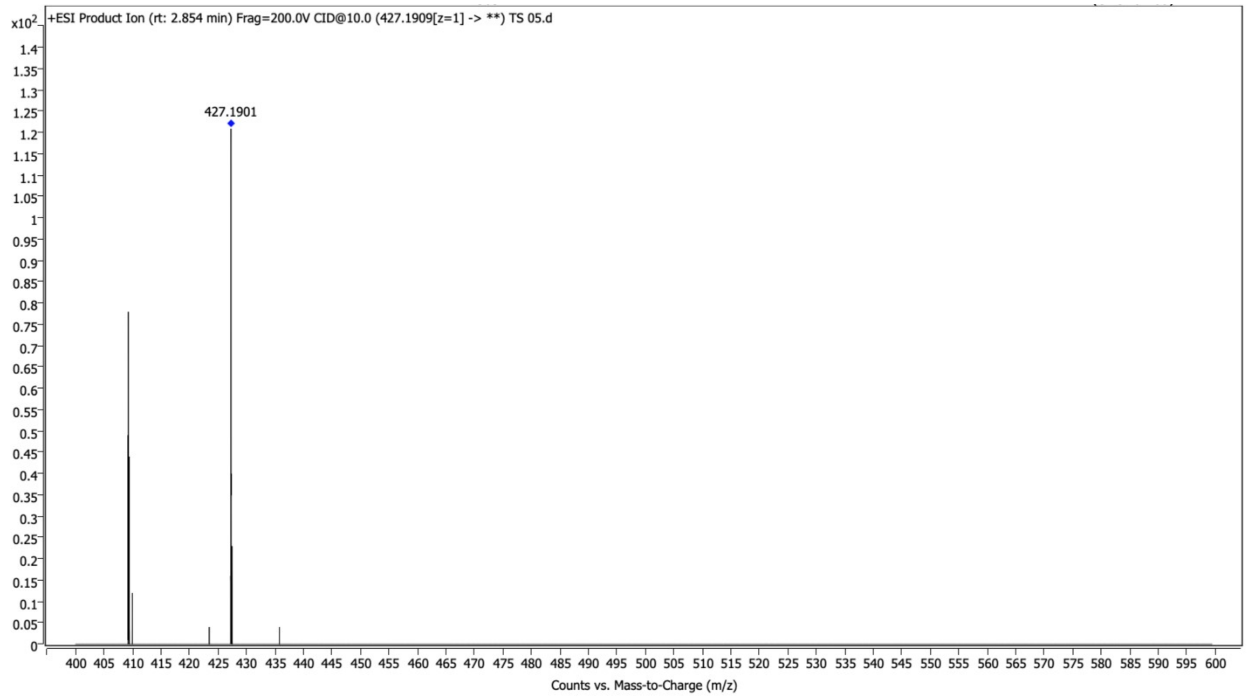

Figure S-4A. The HRESIMS spectrum of **2**.

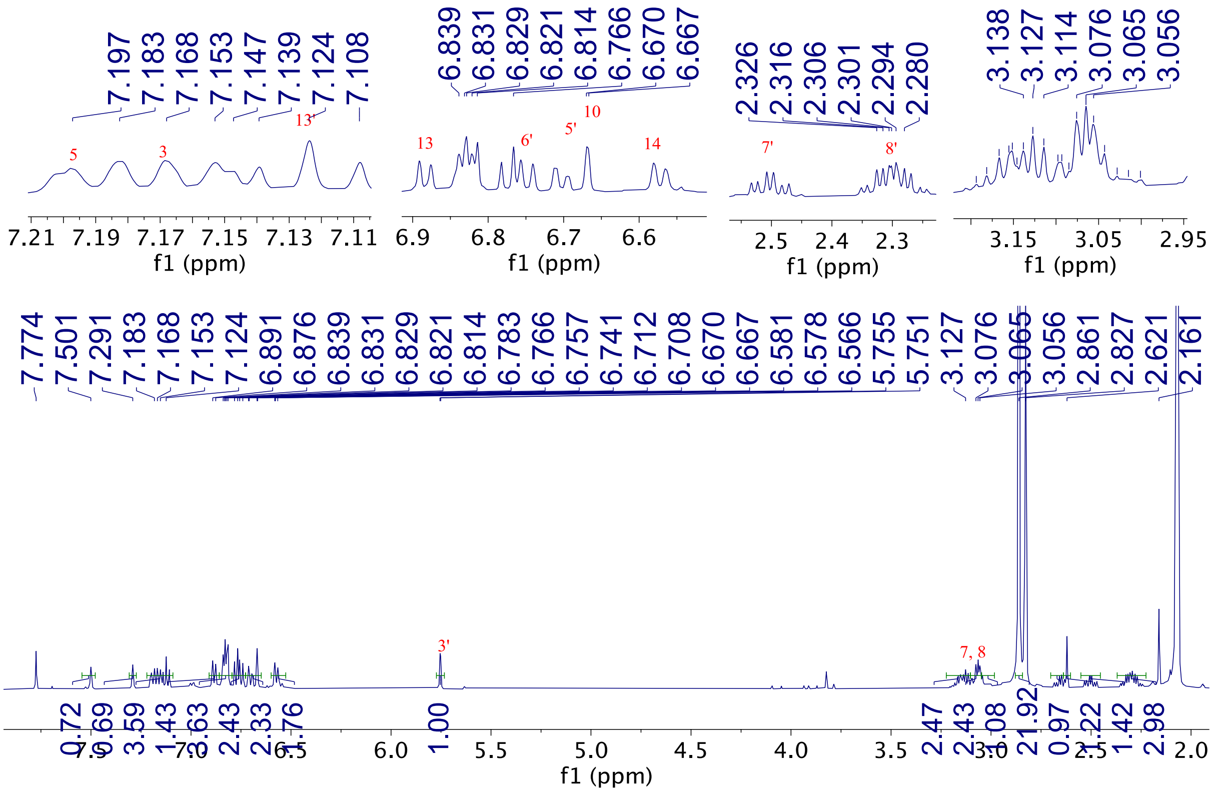

Figure S-4B. The ^{1}H NMR spectrum of **2** in acetone-d_6.

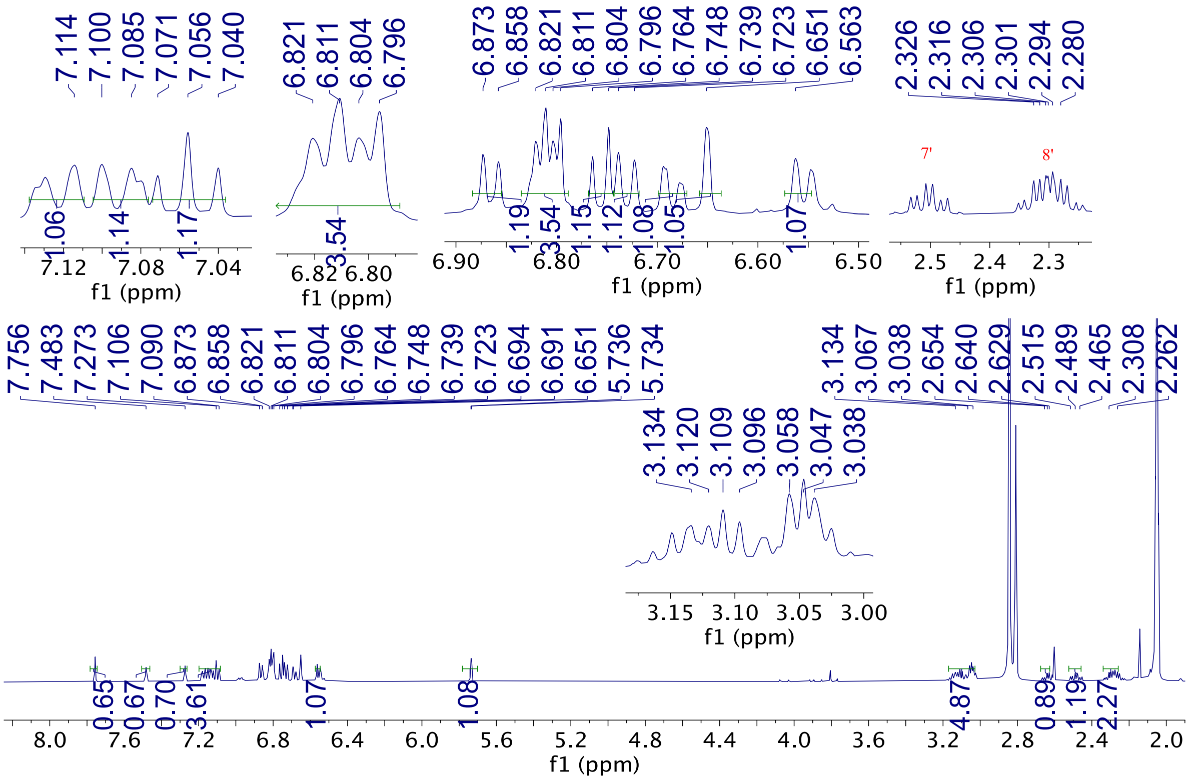

Figure S-4C. The ^{1}H NMR spectrum of **2** in acetone-d_6.

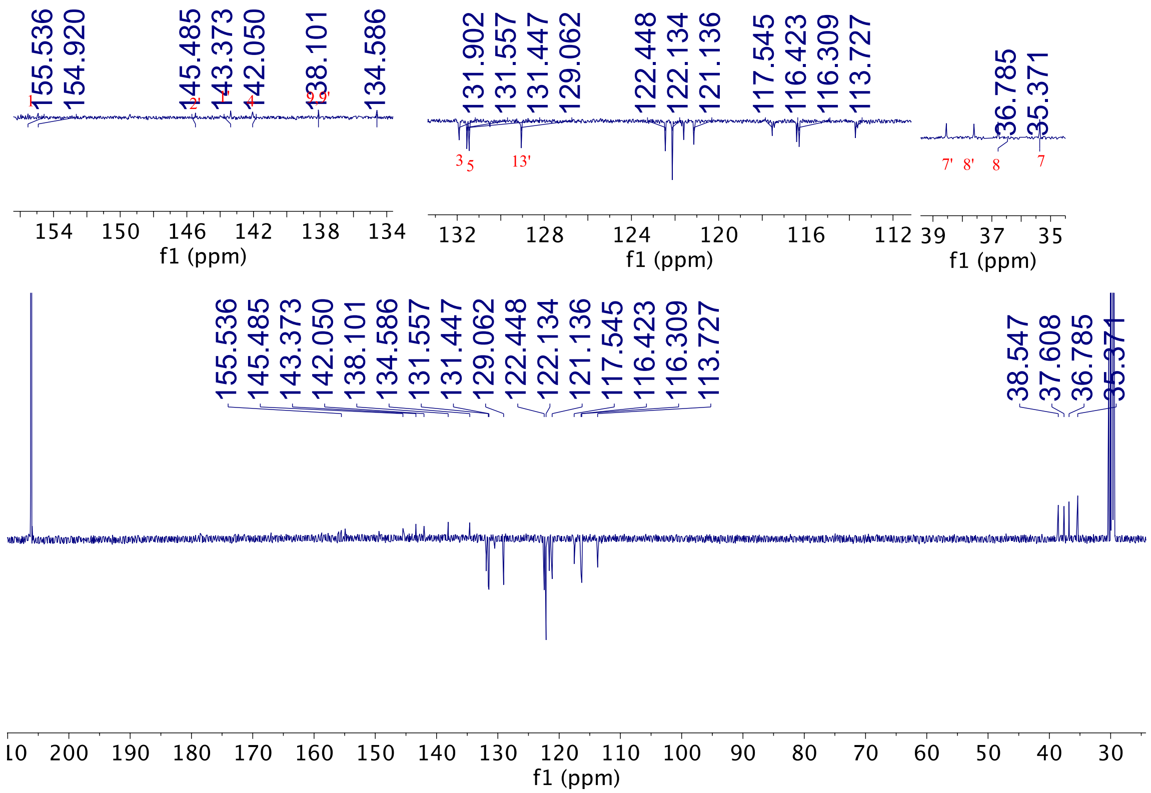

Figure S-4D. The ^{13}C NMR spectrum of **2** in acetone-d_6.

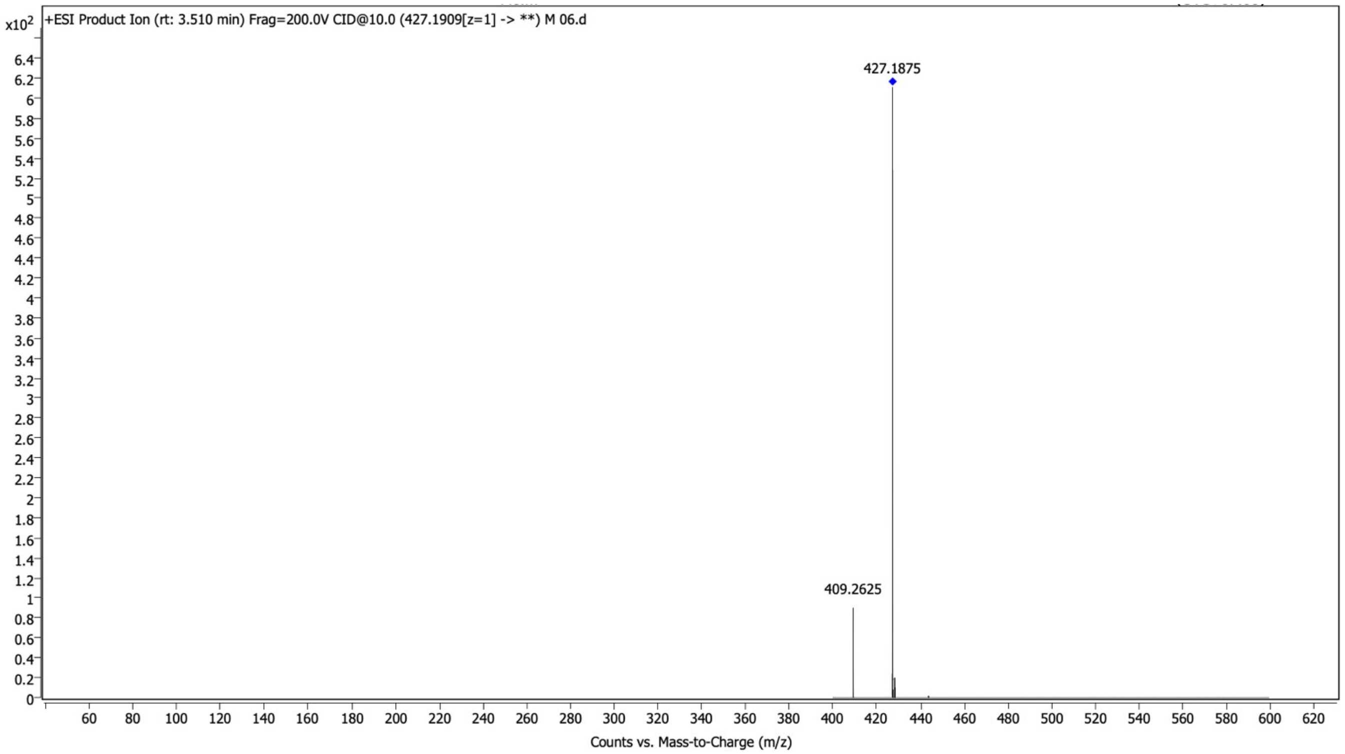

Figure S-5A. The HRESIMS spectrum of **3**.

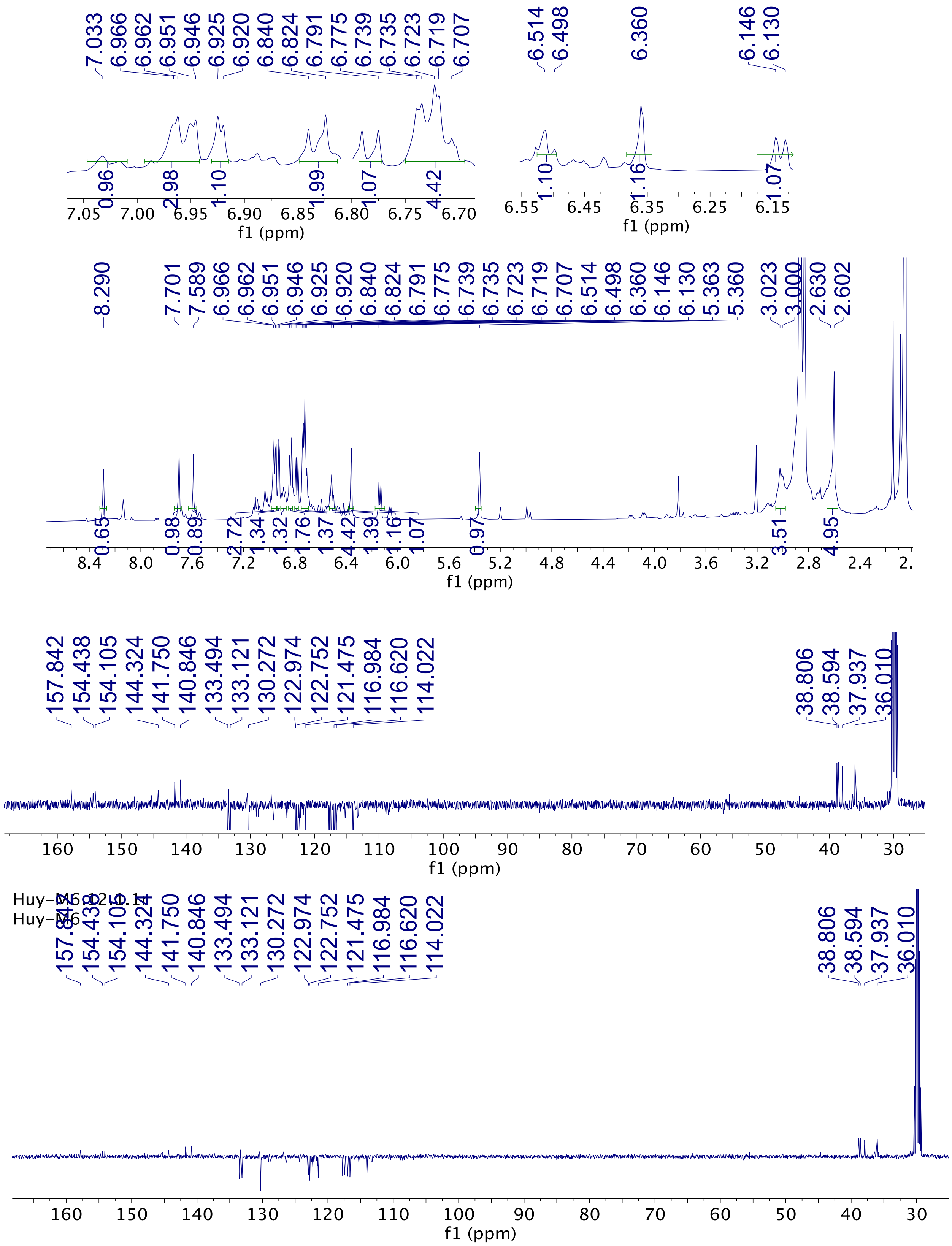

Figure S-5B. The ^{1}H NMR spectrum of **3** in acetone-d_6.

Figure S-5C. The ^{13}C NMR spectrum of **3** in acetone-d_6.

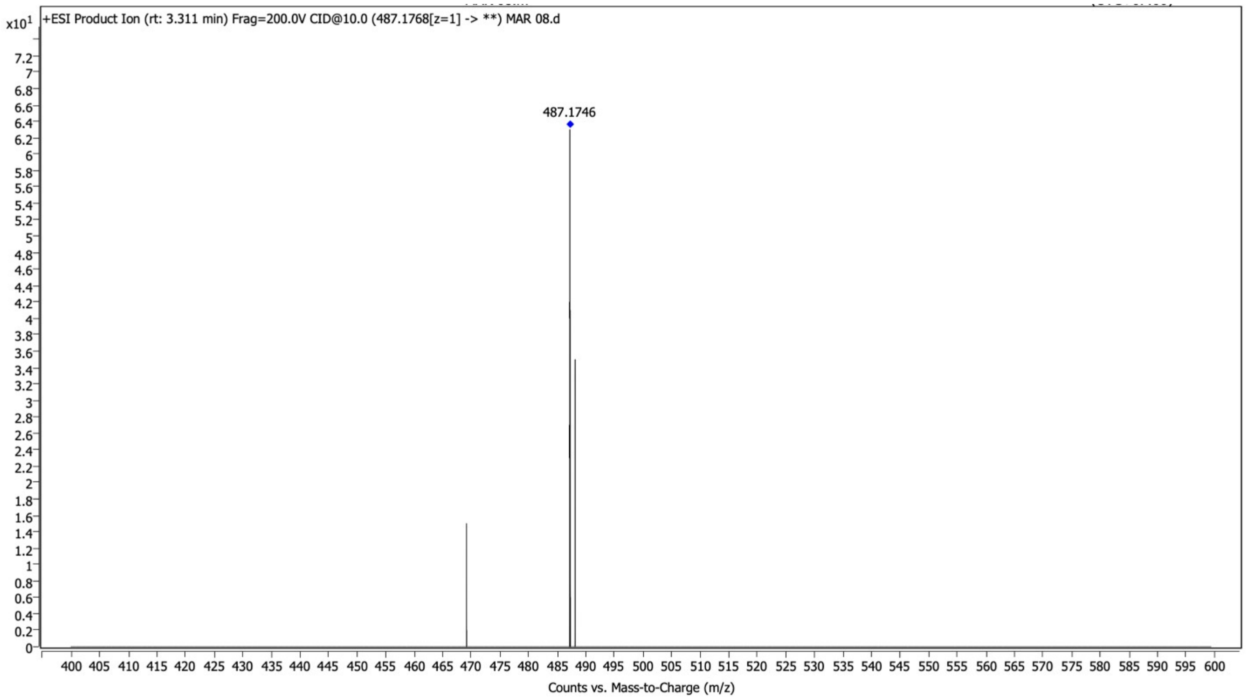

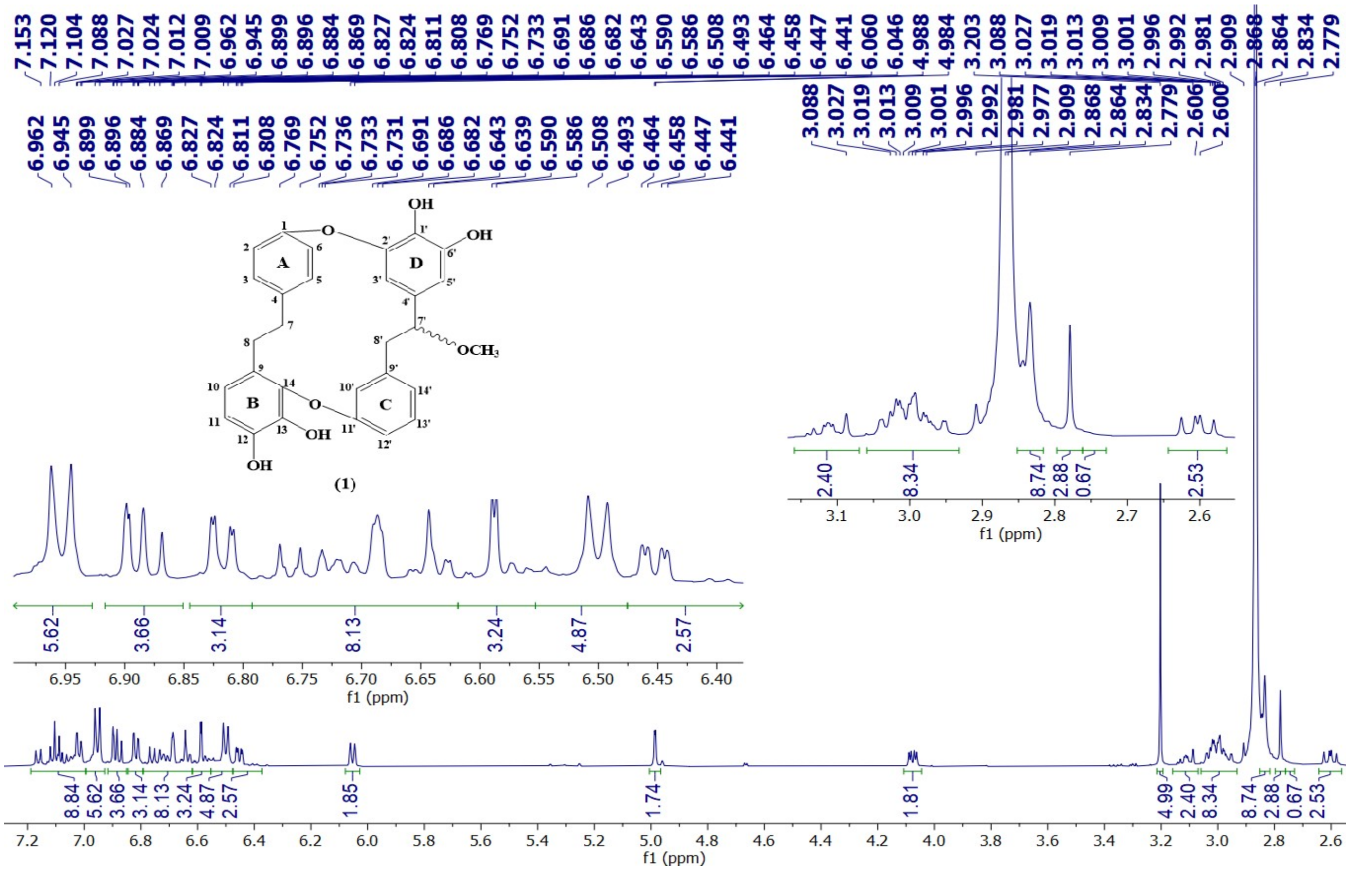

Figure S-6A. The HRESIMS spectrum of **4**.

Figure S-6B. The ^{1}H NMR spectrum of **4** in acetone-d_6.

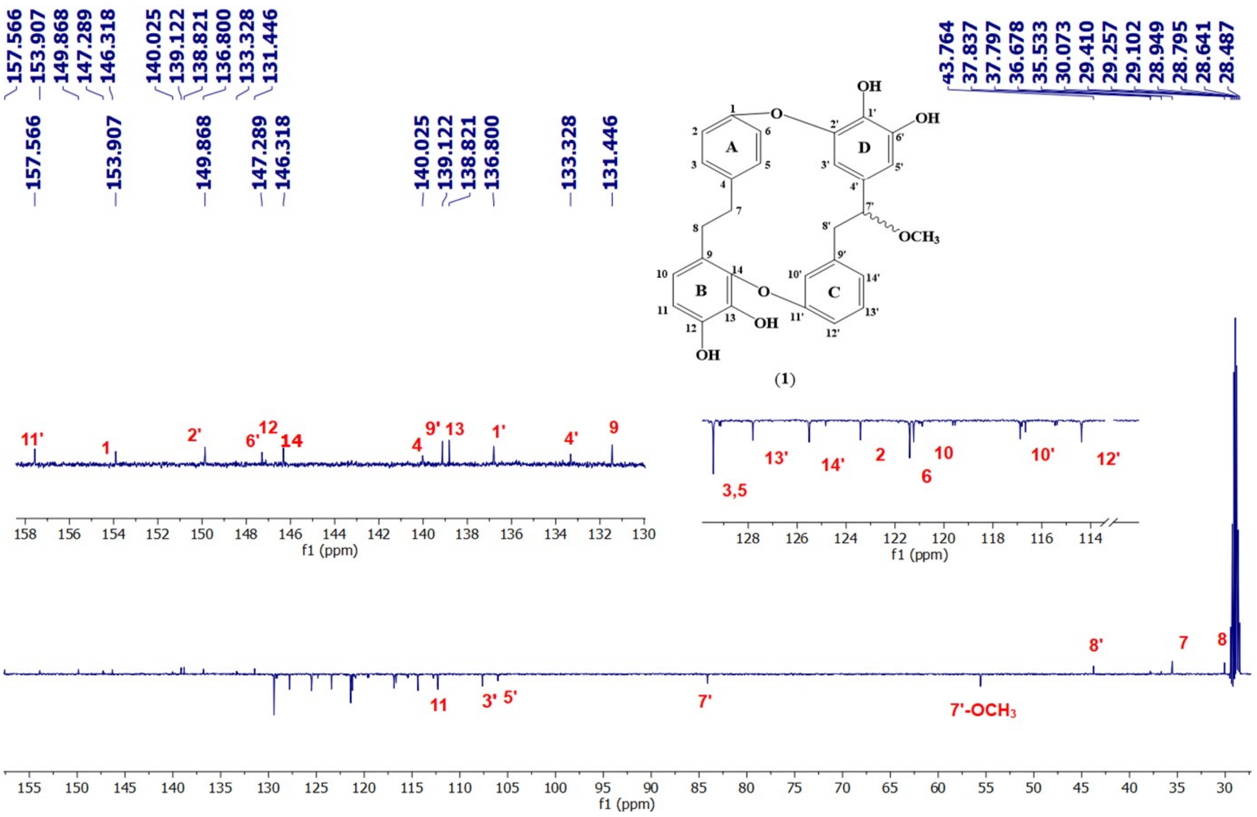

Figure S-6C. The ^{13}C NMR spectrum of **4** in acetone-d_6.

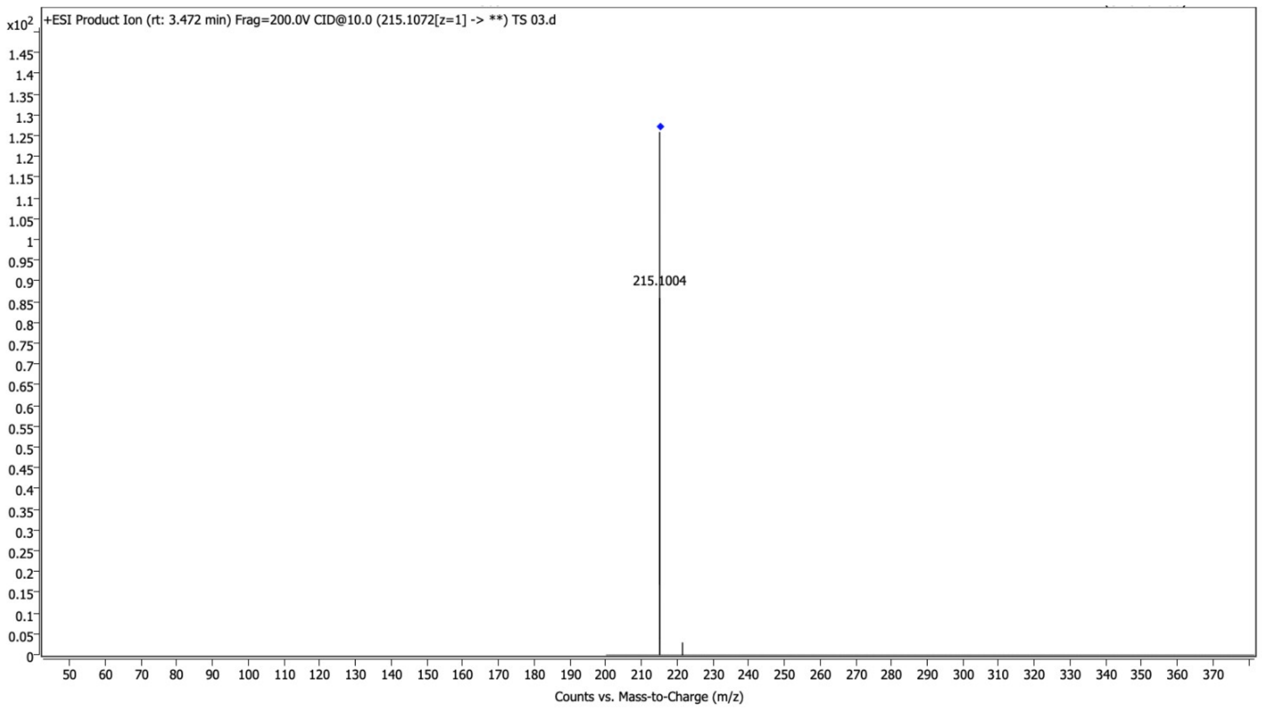

Figure S-7A. The HRESIMS spectrum of **5.**

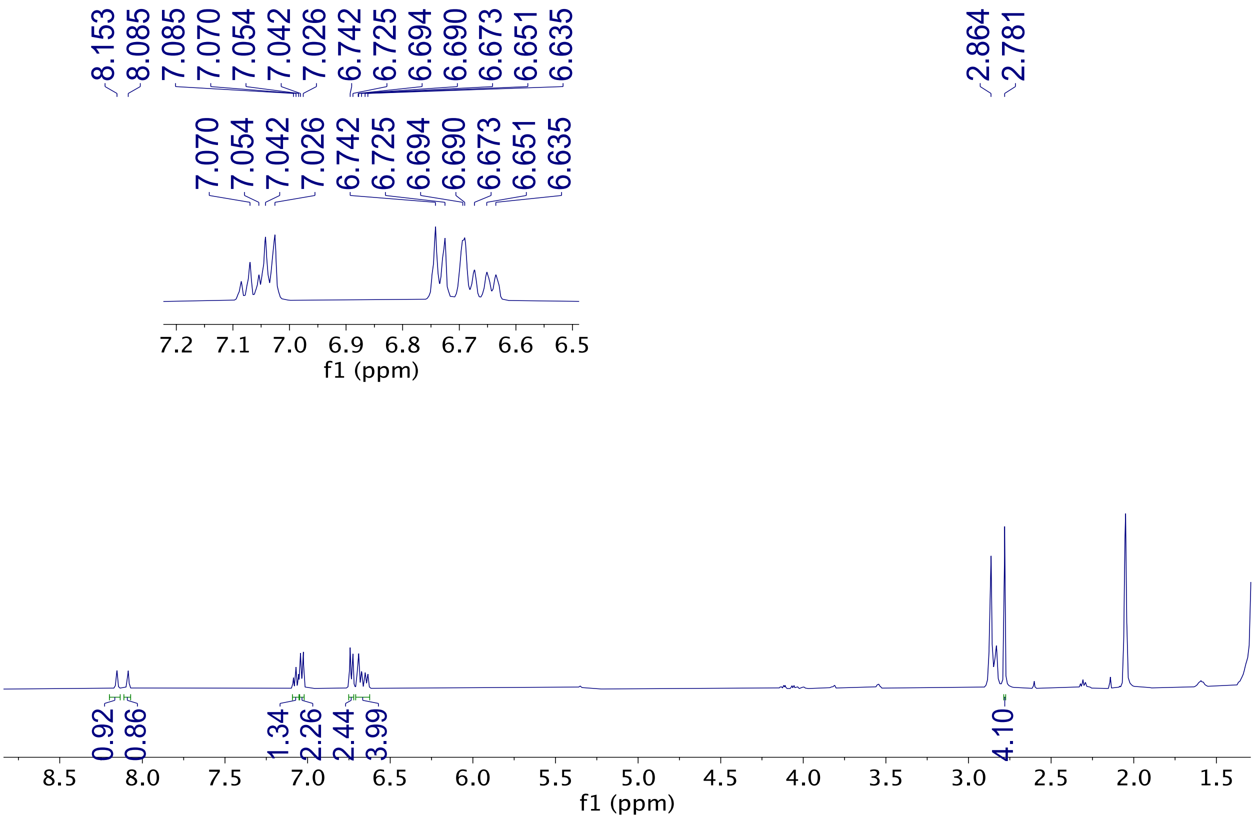

Figure S-7B. The ^{1}H NMR spectrum of **5** in acetone-d_6.

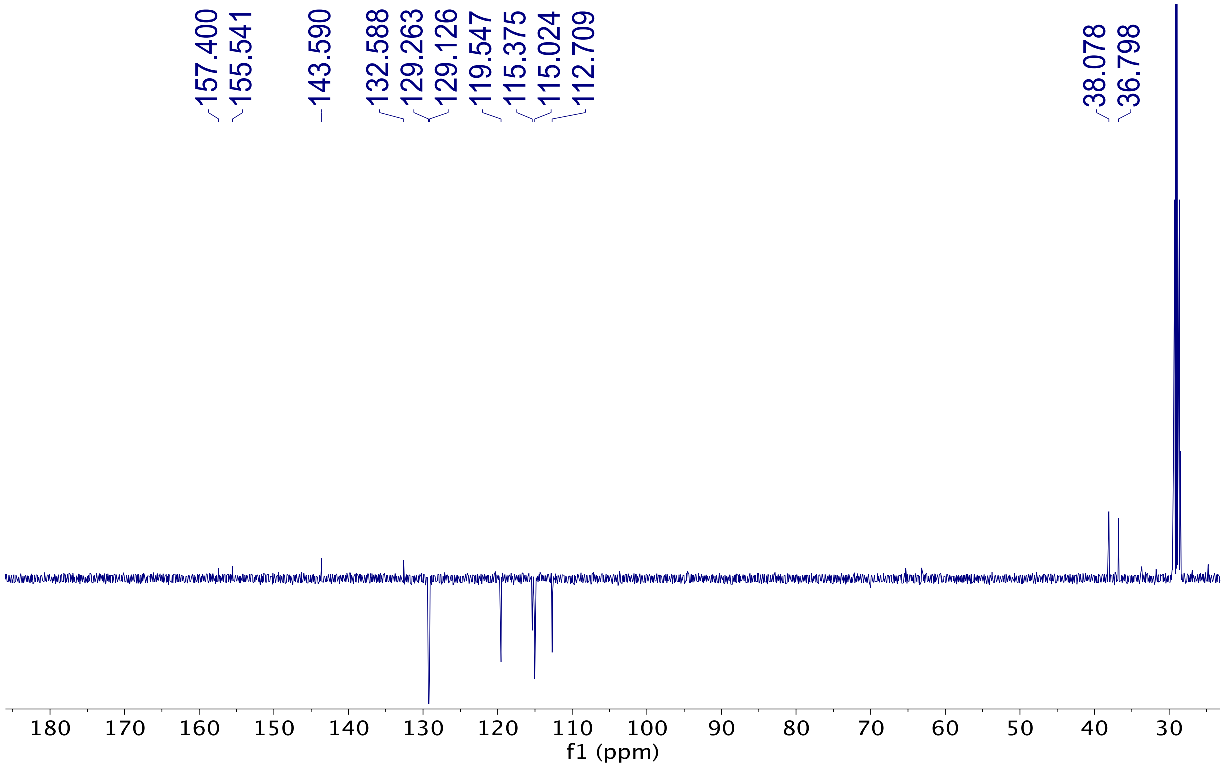

Figure S-7C. The ^{13}C NMR spectrum of **5** in acetone-d_6.

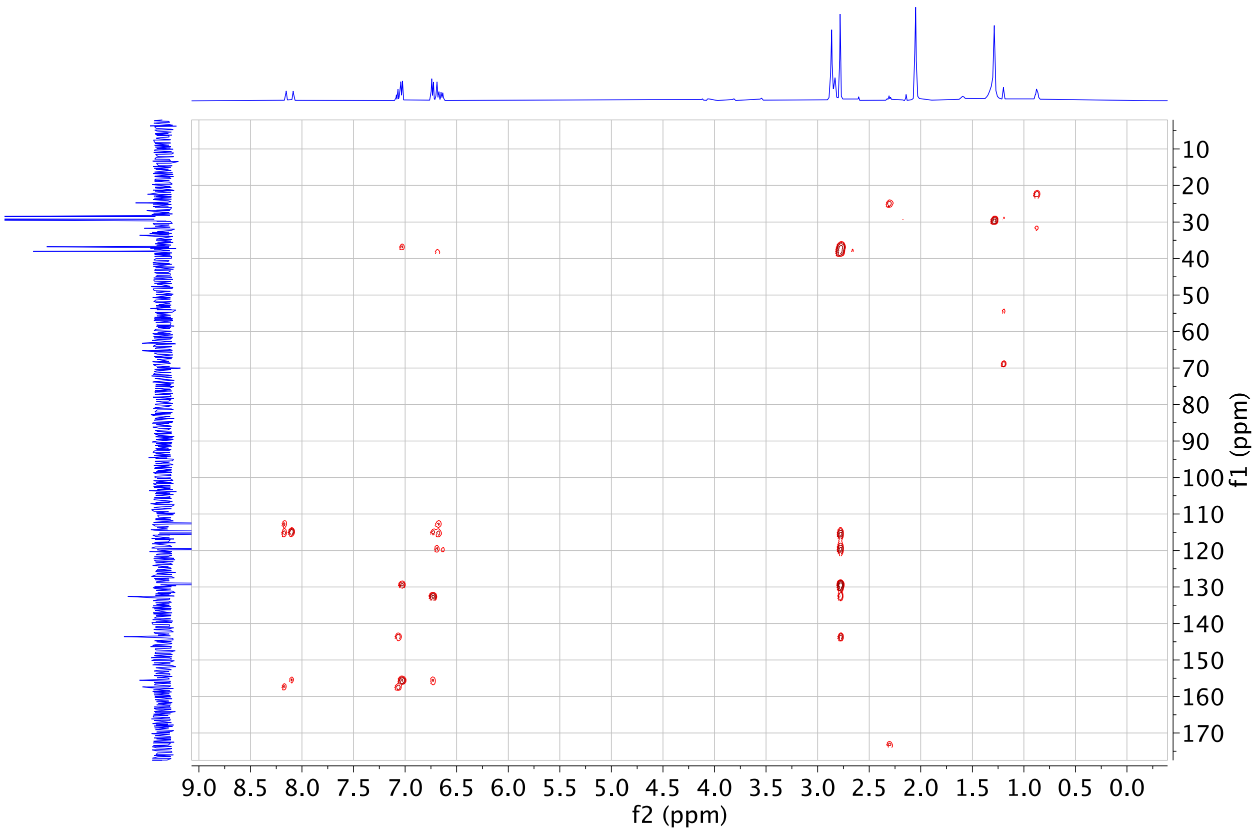

Figure S-7D. The HMBC spectrum of **5** in acetone-d_6.

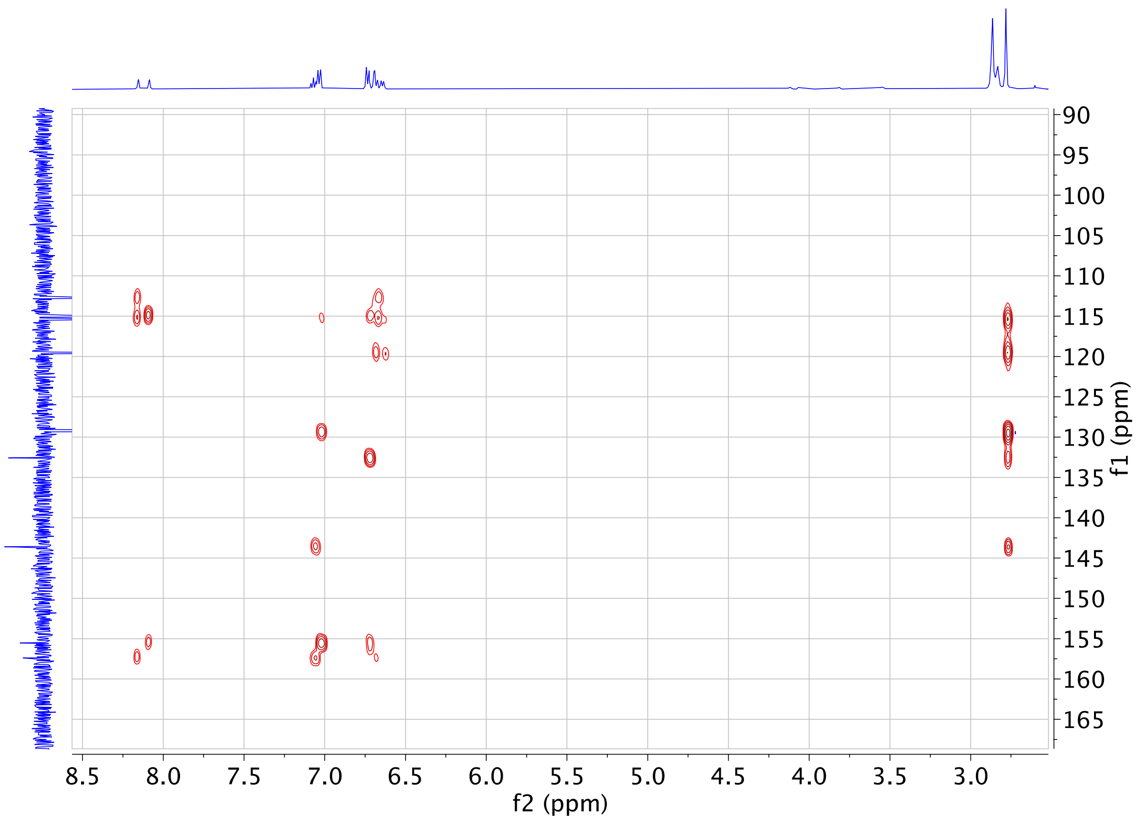

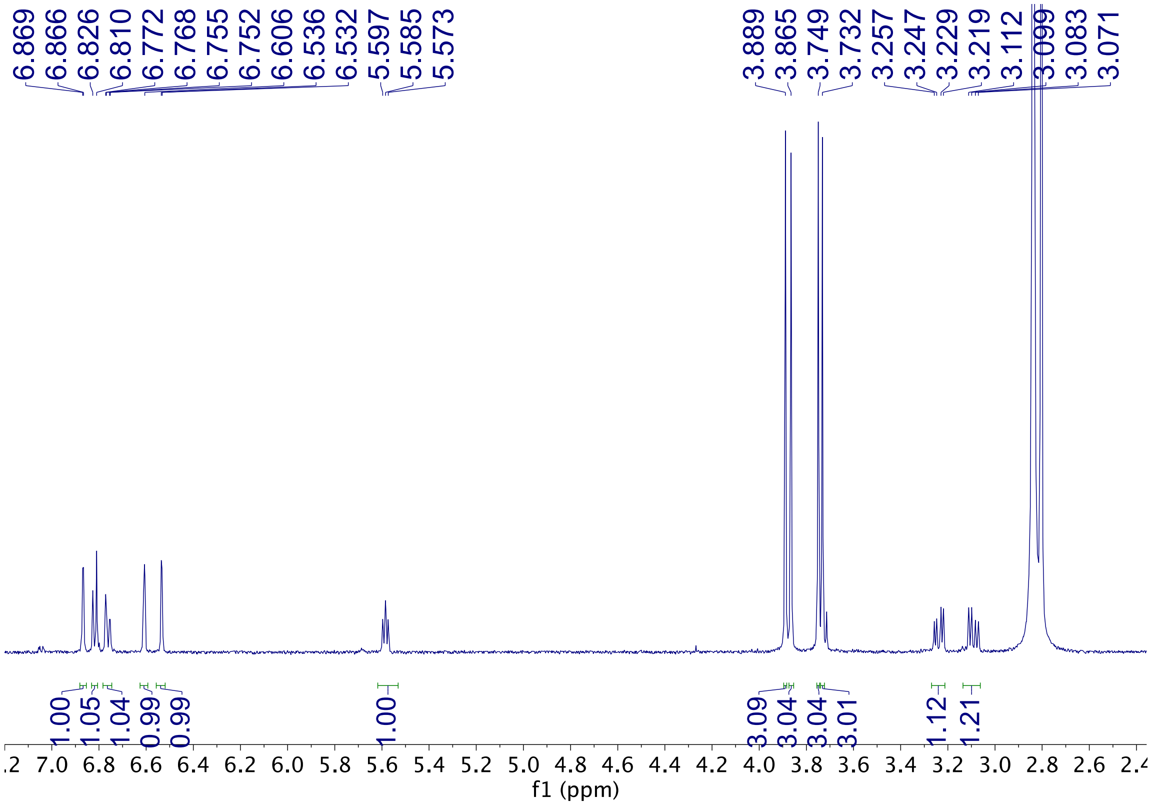

Figure S-7E. The HMBC spectrum of **5** in acetone-d_6

Figure S-8A. The ^{1}H NMR spectrum of **6** in acetone-d$_{6}$.

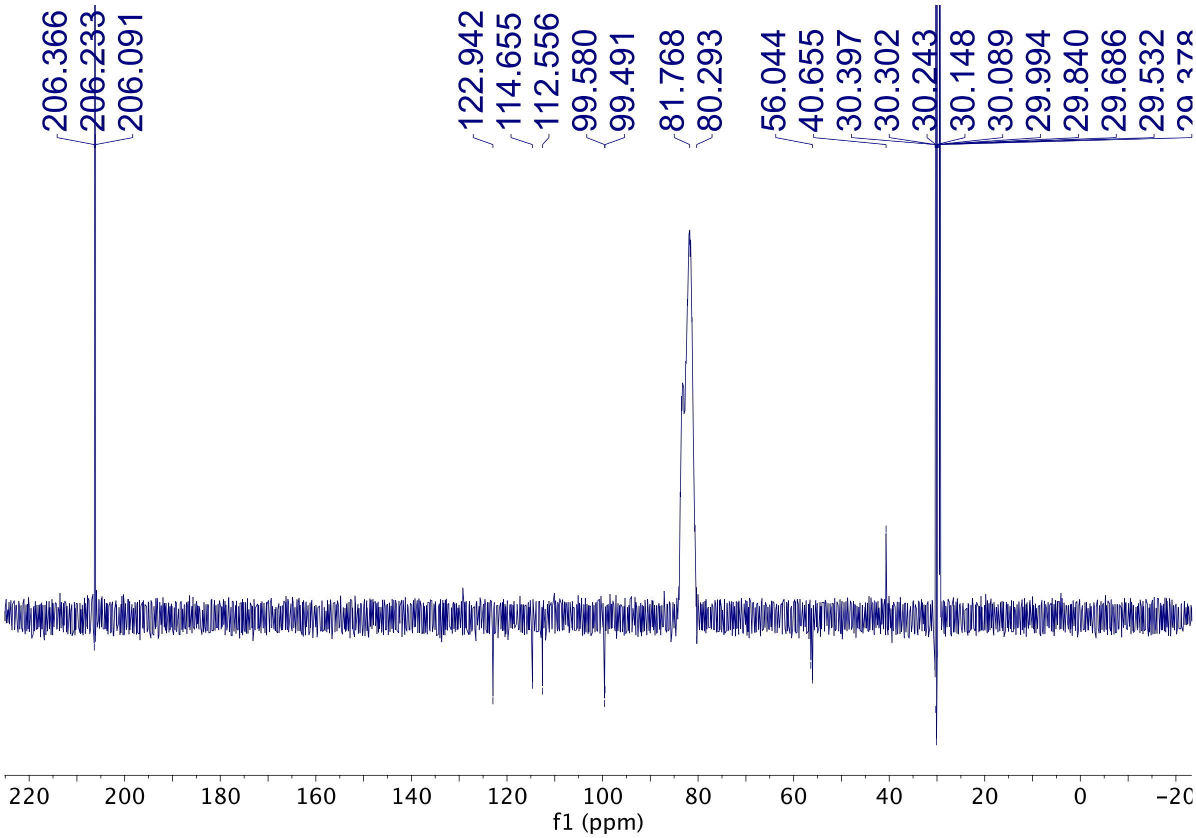

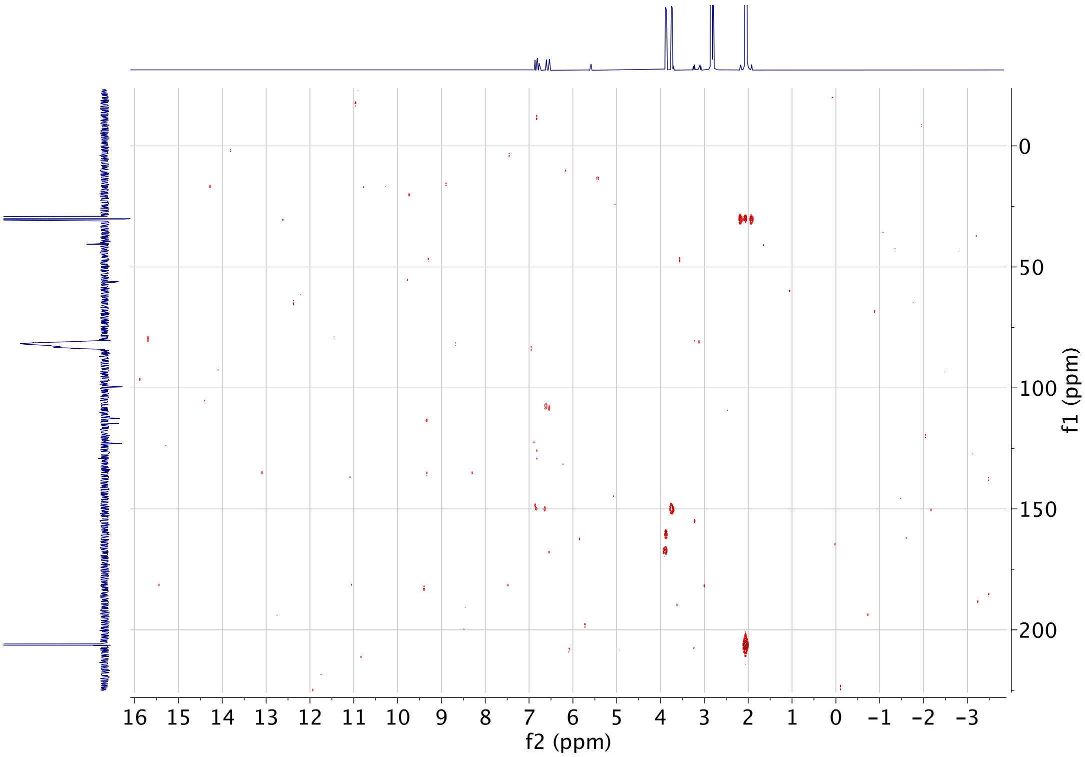

Figure S-8B. The ^{13}C NMR spectrum of **6** in acetone-d_6.

Figure S-8C. The HMBC spectrum of **6** in acetone-d_6.

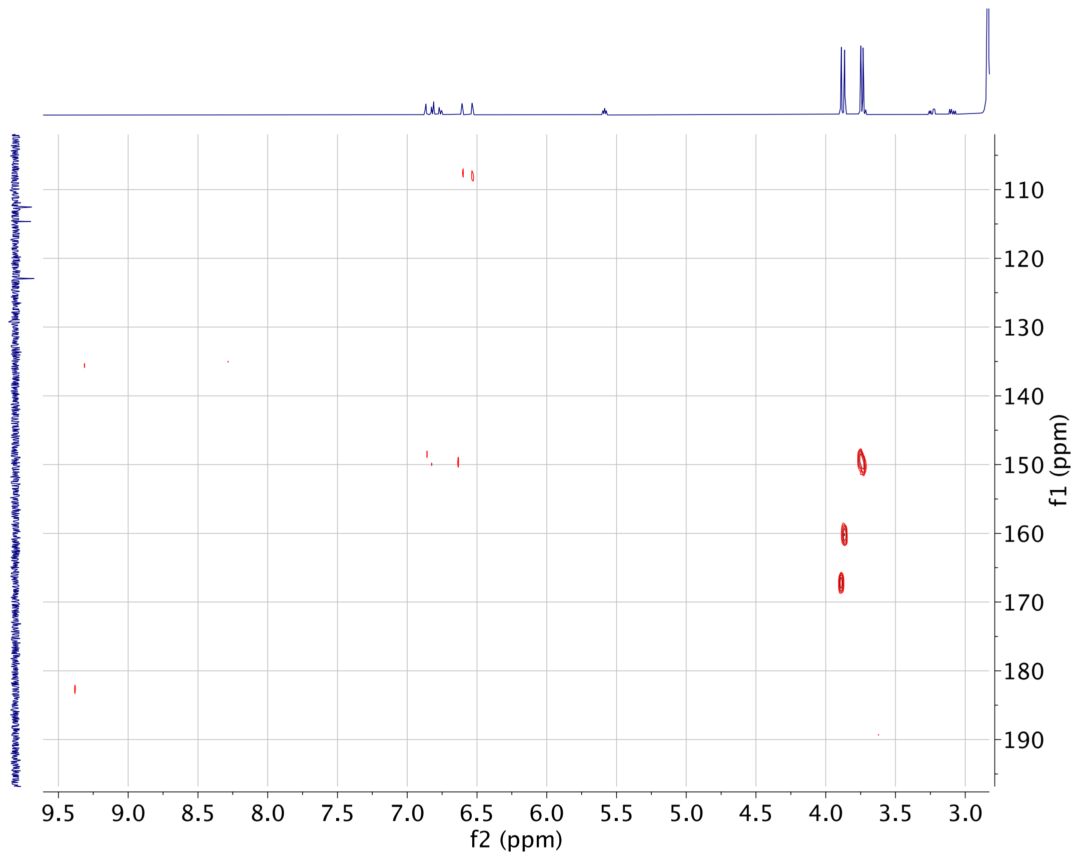

Figure S-8D. The HMBC spectrum of **6** in acetone-d_6.

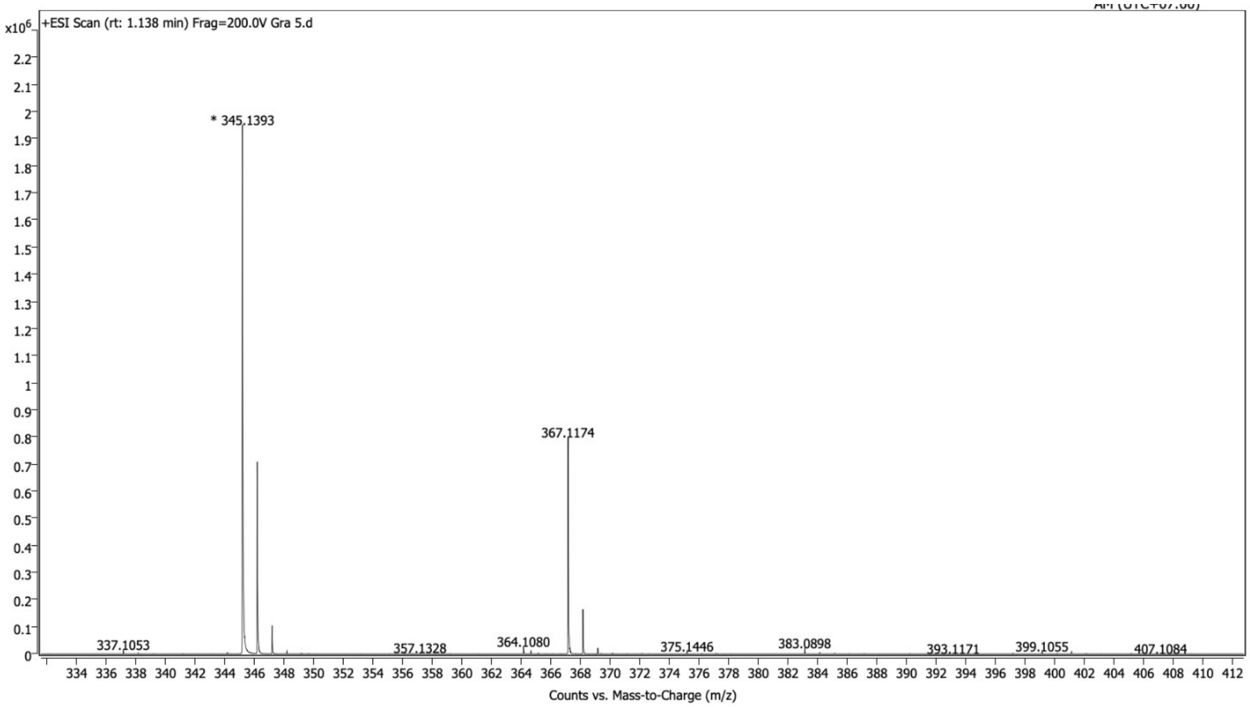

Figure S-8E. The HRESIMS spectrum of **6**.

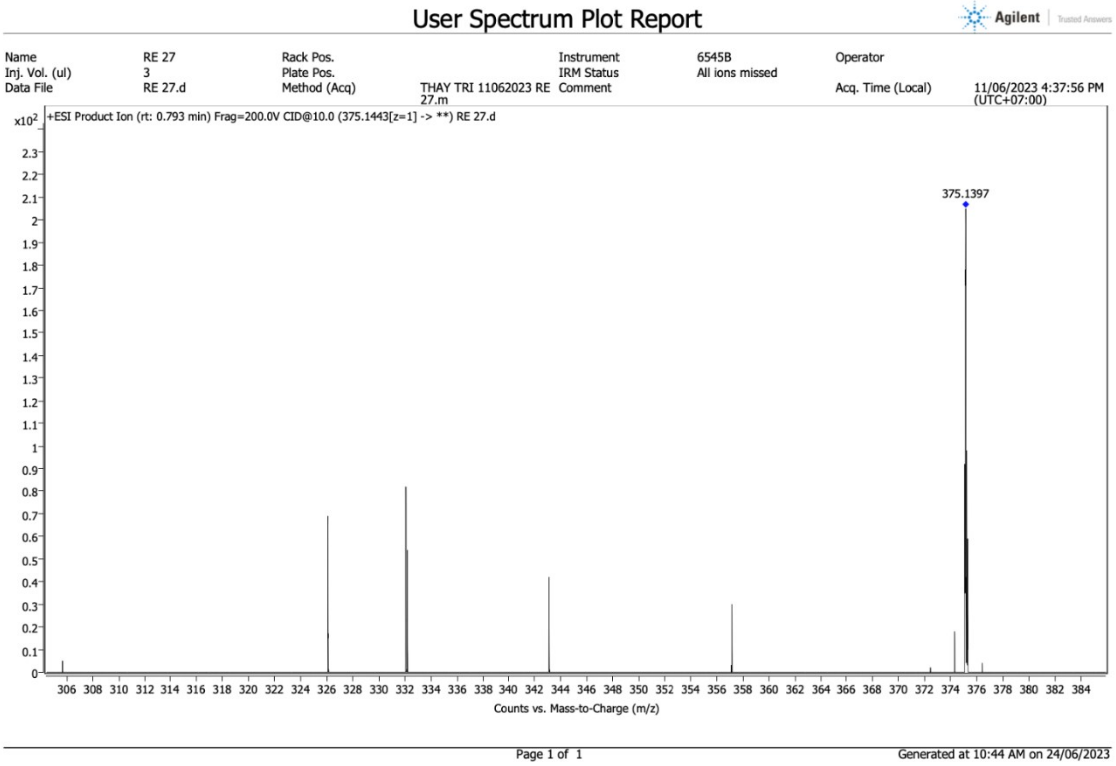

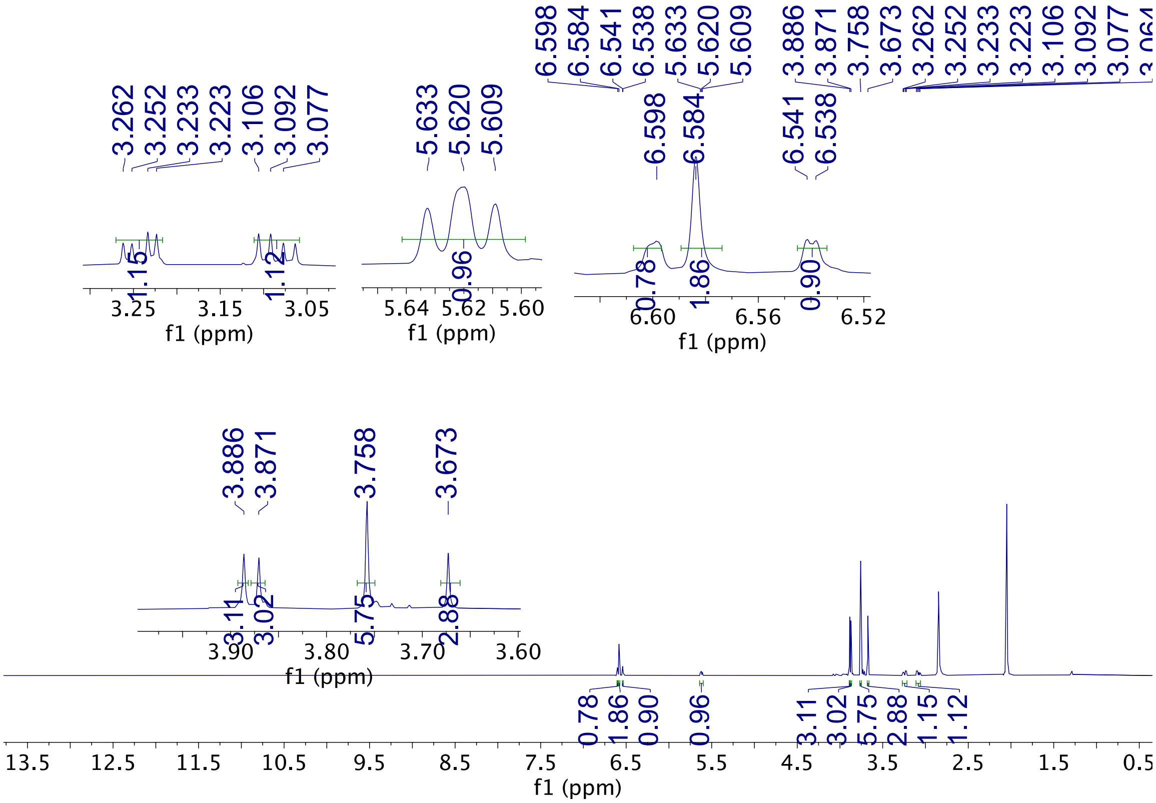

Figure S-9A. The HRESIMS spectrum of **7**.

Figure S-9B. The ^{1}H NMR spectrum of **7** in acetone-d_6.

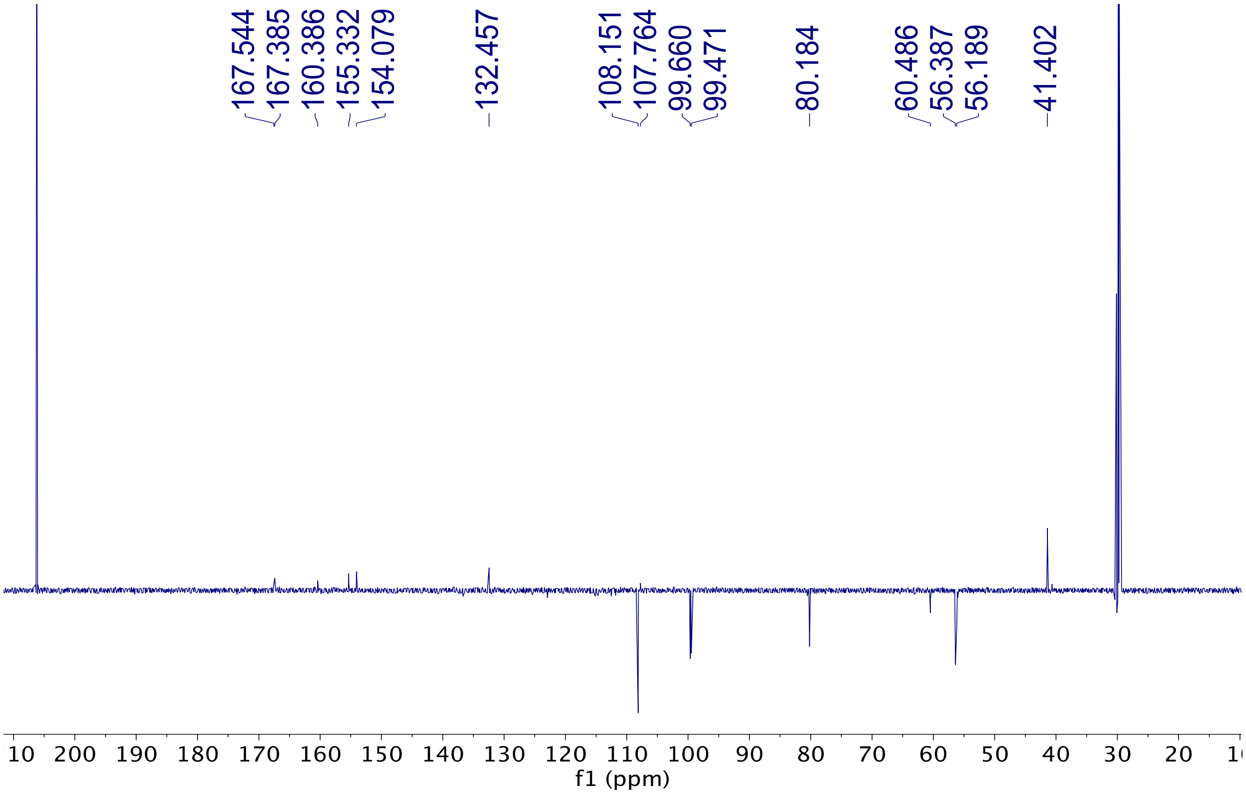

Figure S-9C. The ^{13}C NMR spectrum of **7** in acetone-d_6.

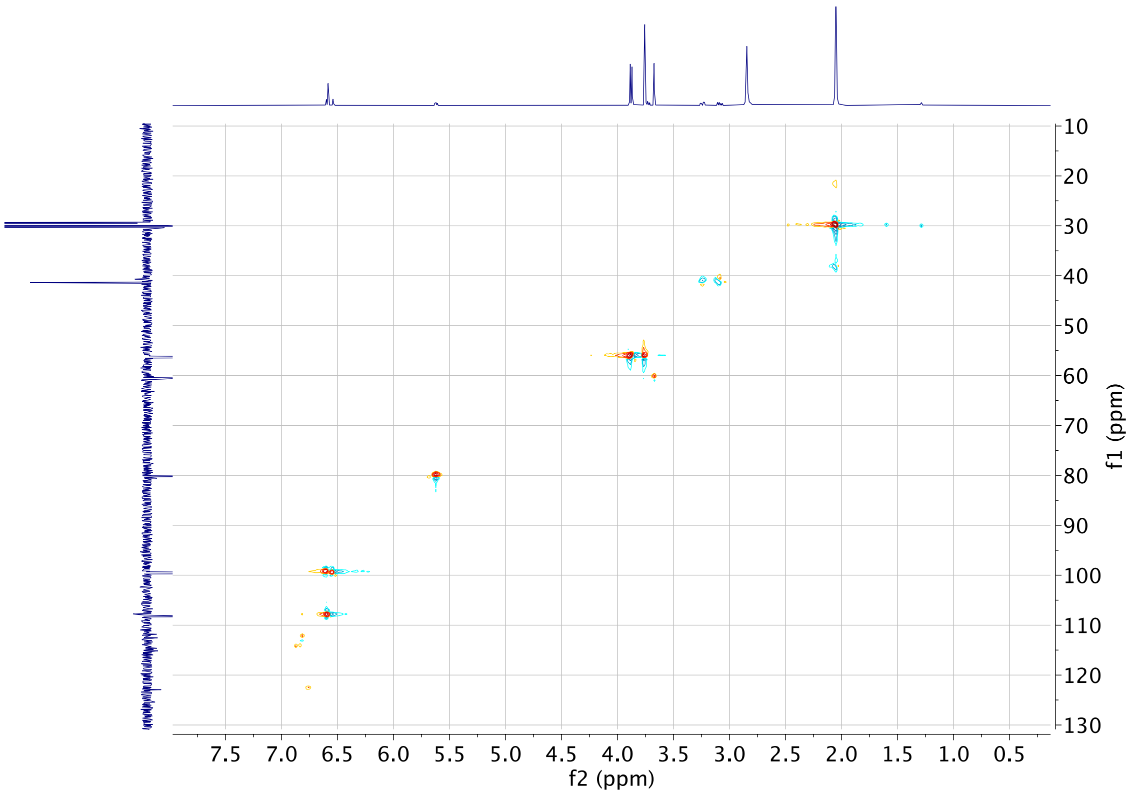

Figure S-9D. The HSQC spectrum of **7** in acetone-d_6.

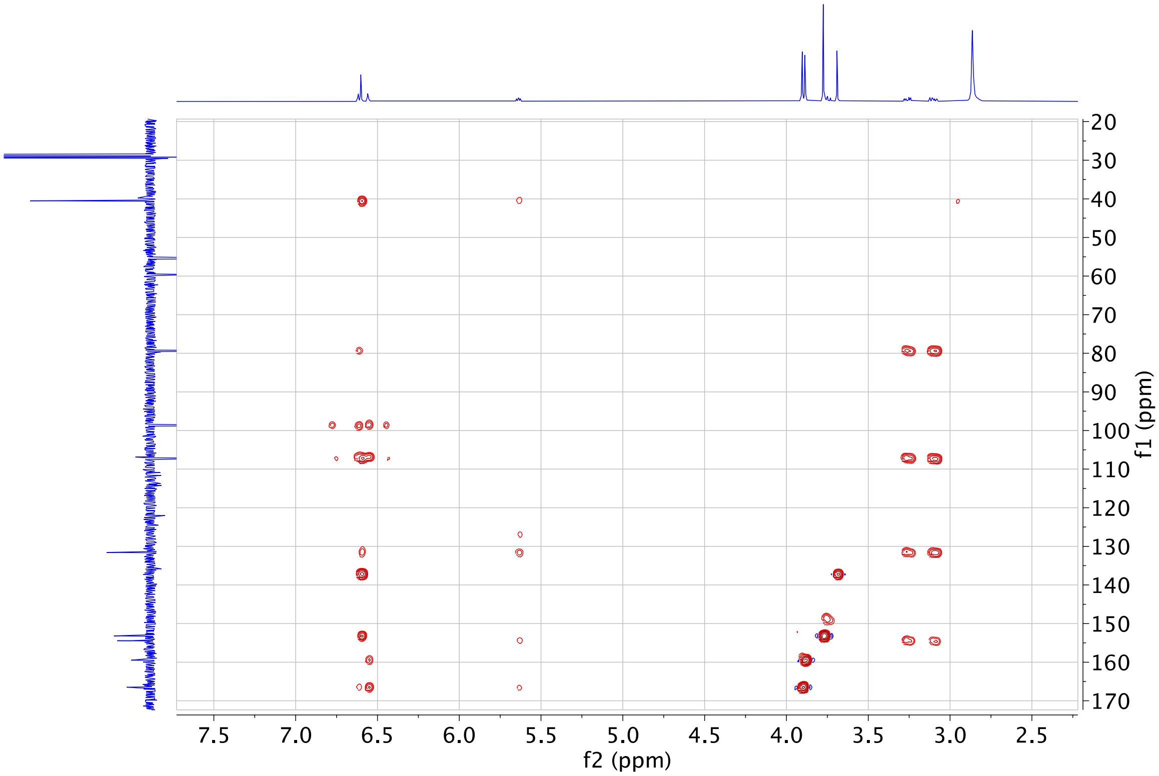

Figure S-9E. The HMBC spectrum of **7** in acetone-d_6

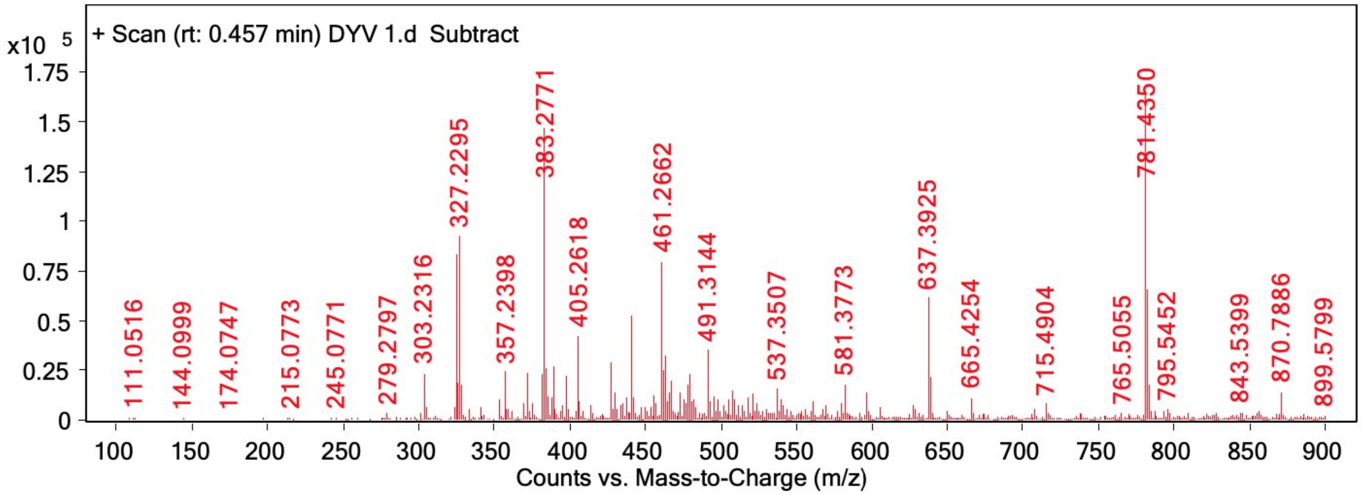

Figure S-10A. The HRESIMS spectrum of **8**.

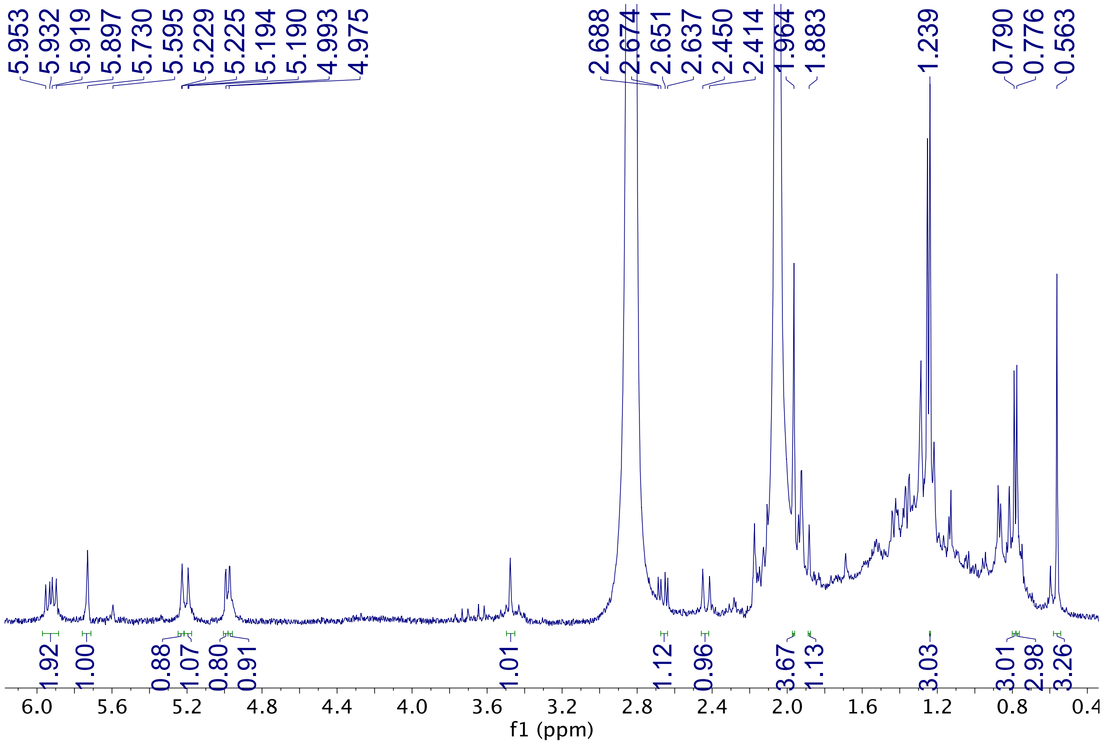

Figure S-10B. The ^{1}H NMR spectrum of **8** in acetone-d_6.

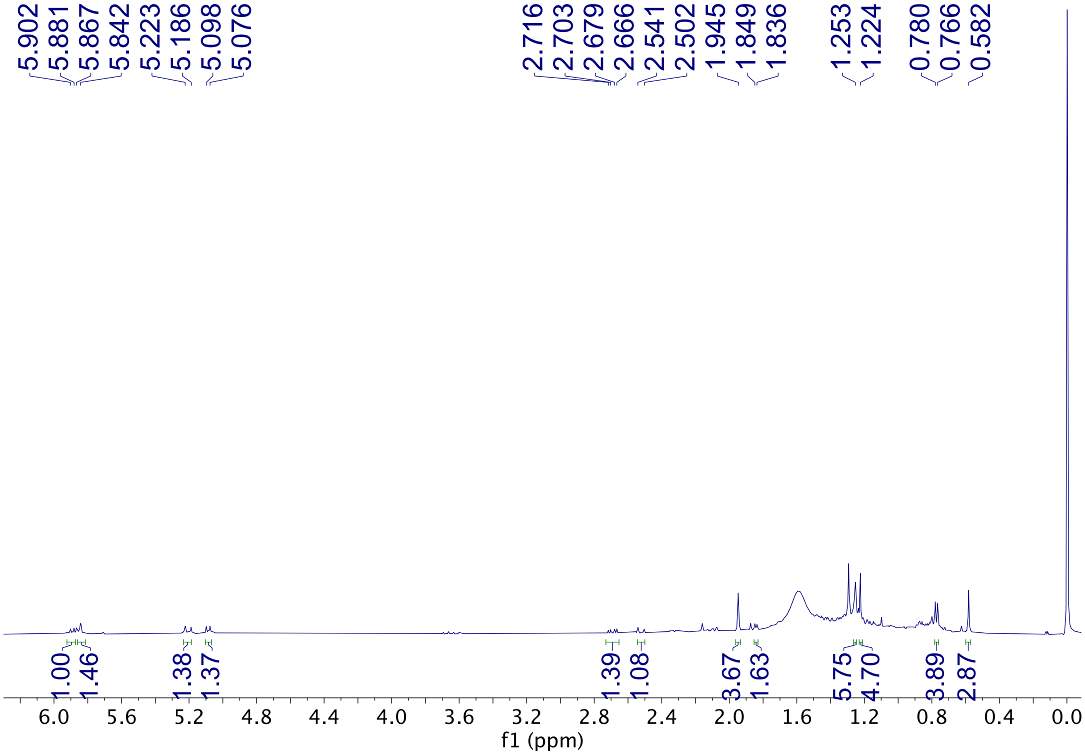

Figure S-10C. The ^{1}H NMR spectrum of **8** in $CDCl_3$.

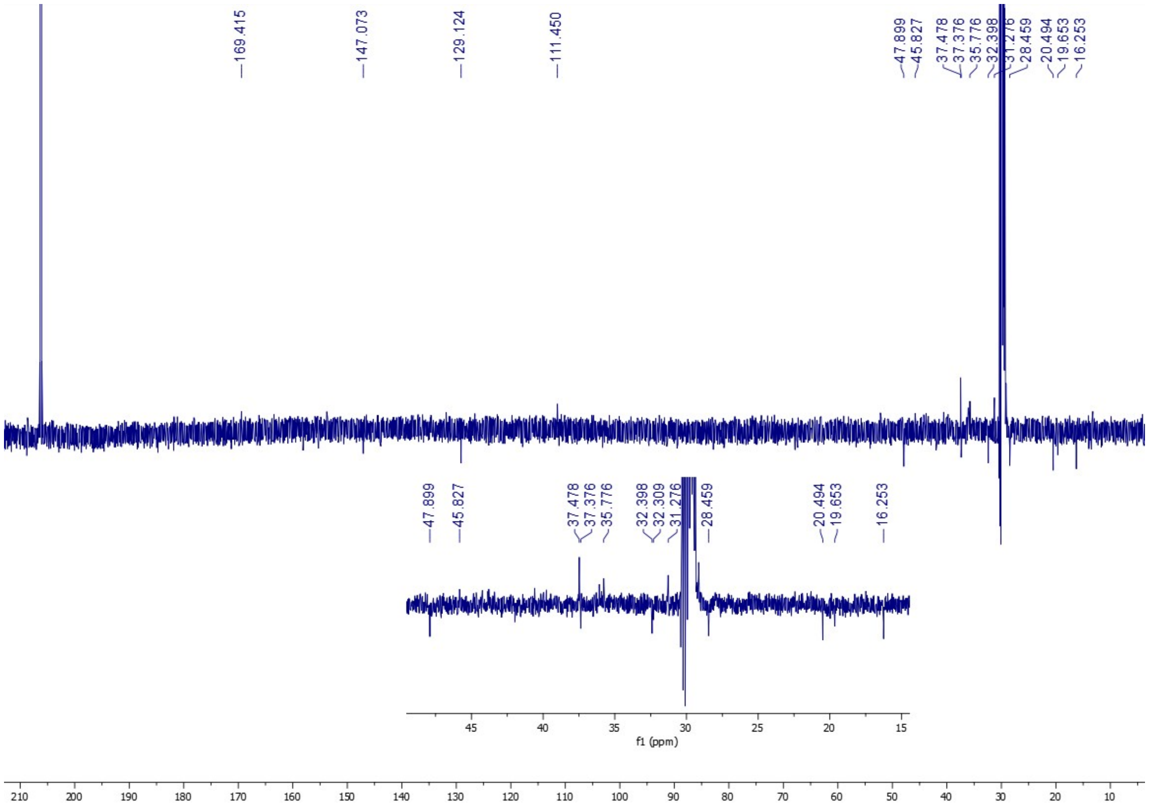

Figure S-10D. The ^{13}C NMR spectrum of **8** in acetone-d_6

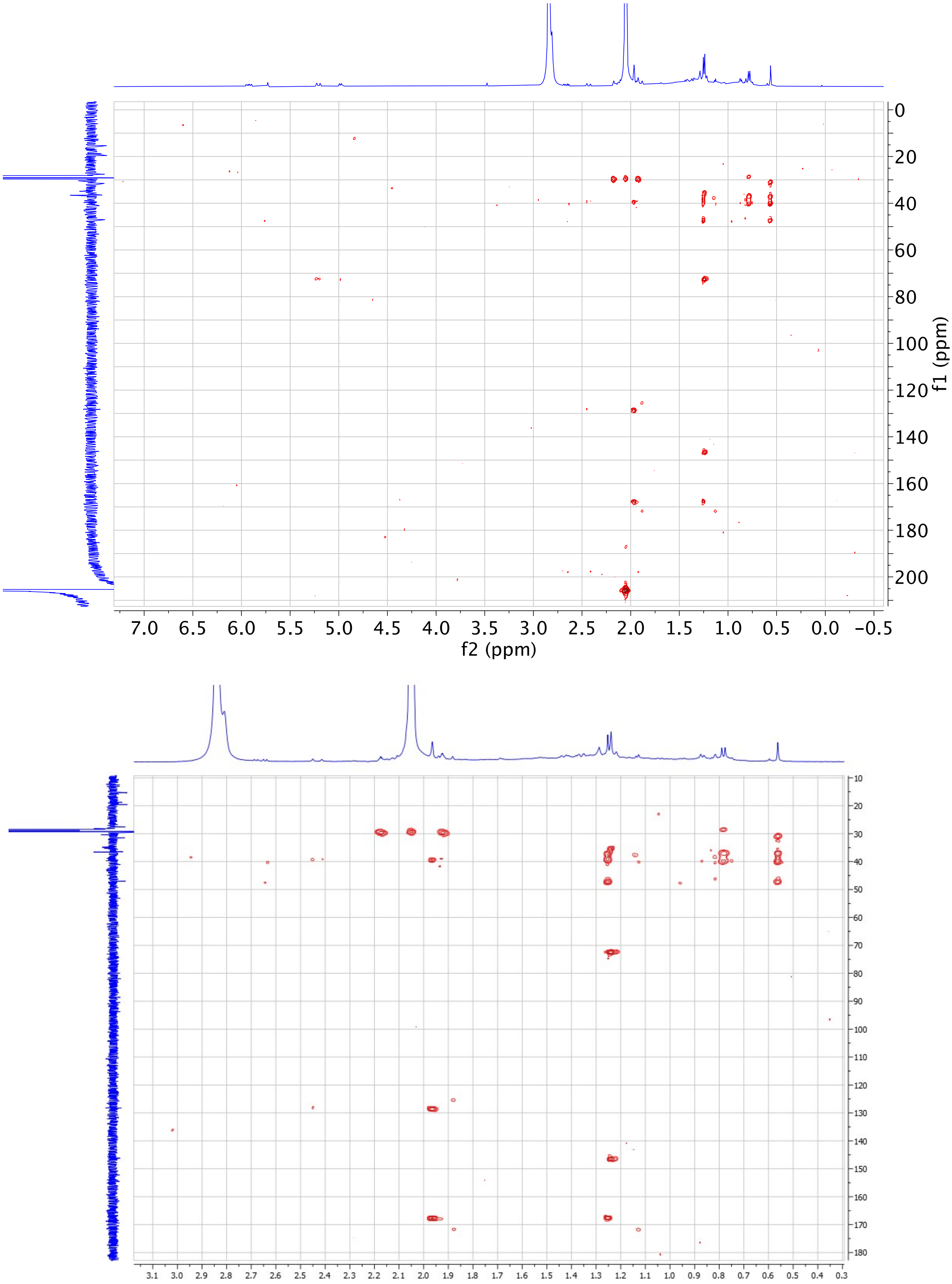

Figure S-10E. The HMBC spectrum of **8** in acetone-d_6.

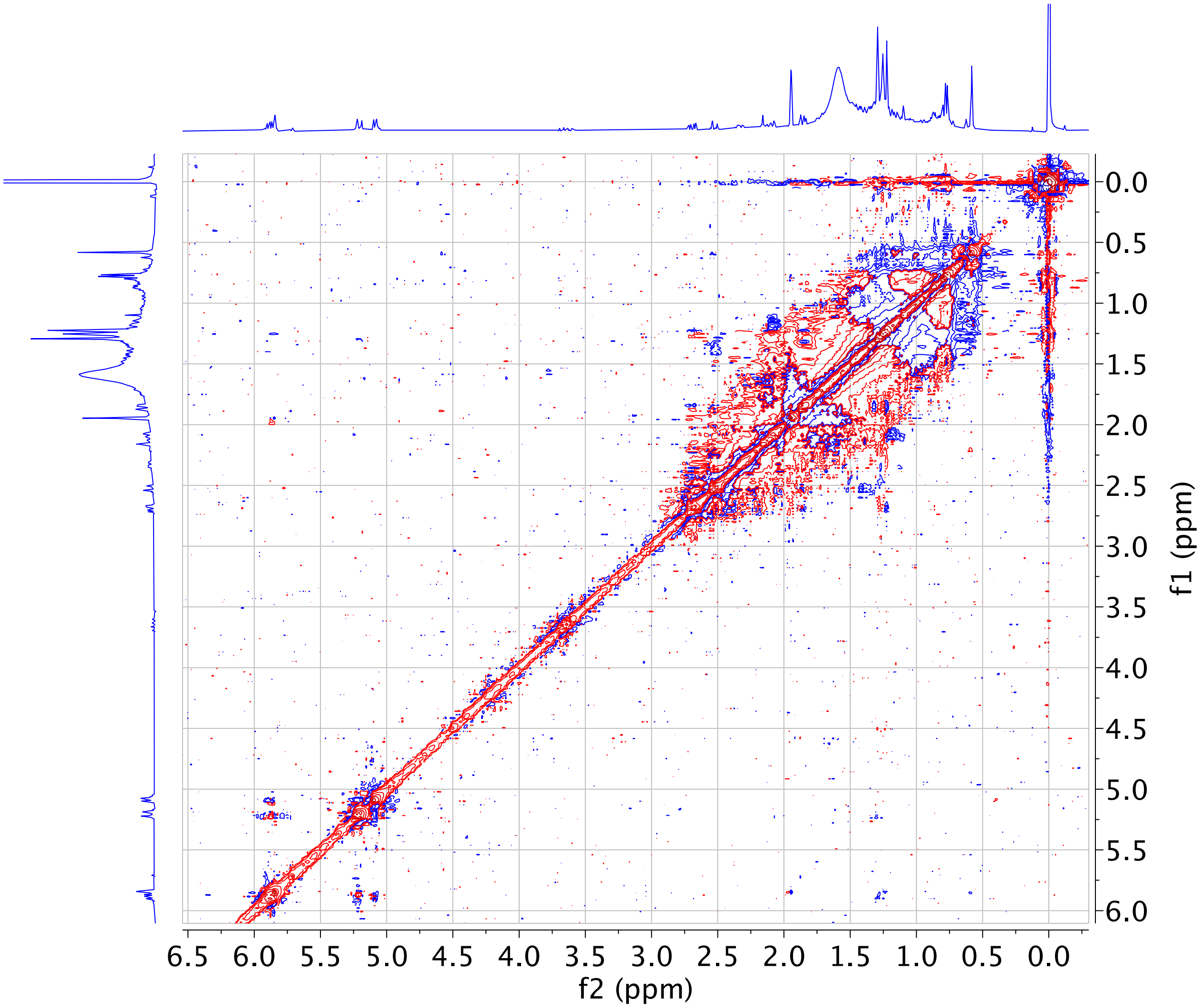

Figure S-10F. The NOESY spectrum of **8** in $CDCl_3$.

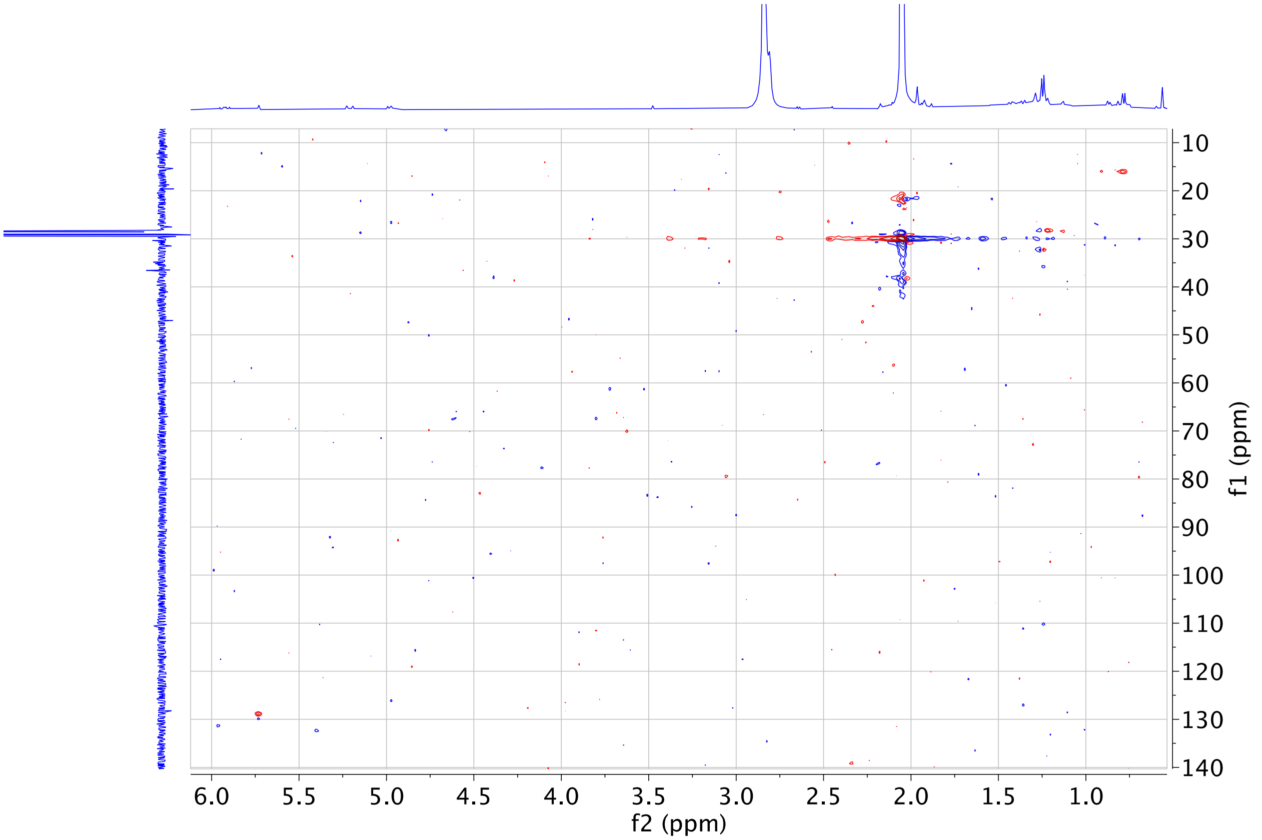

Figure S-10G. The HSQC spectrum of **8** in acetone-d_6.

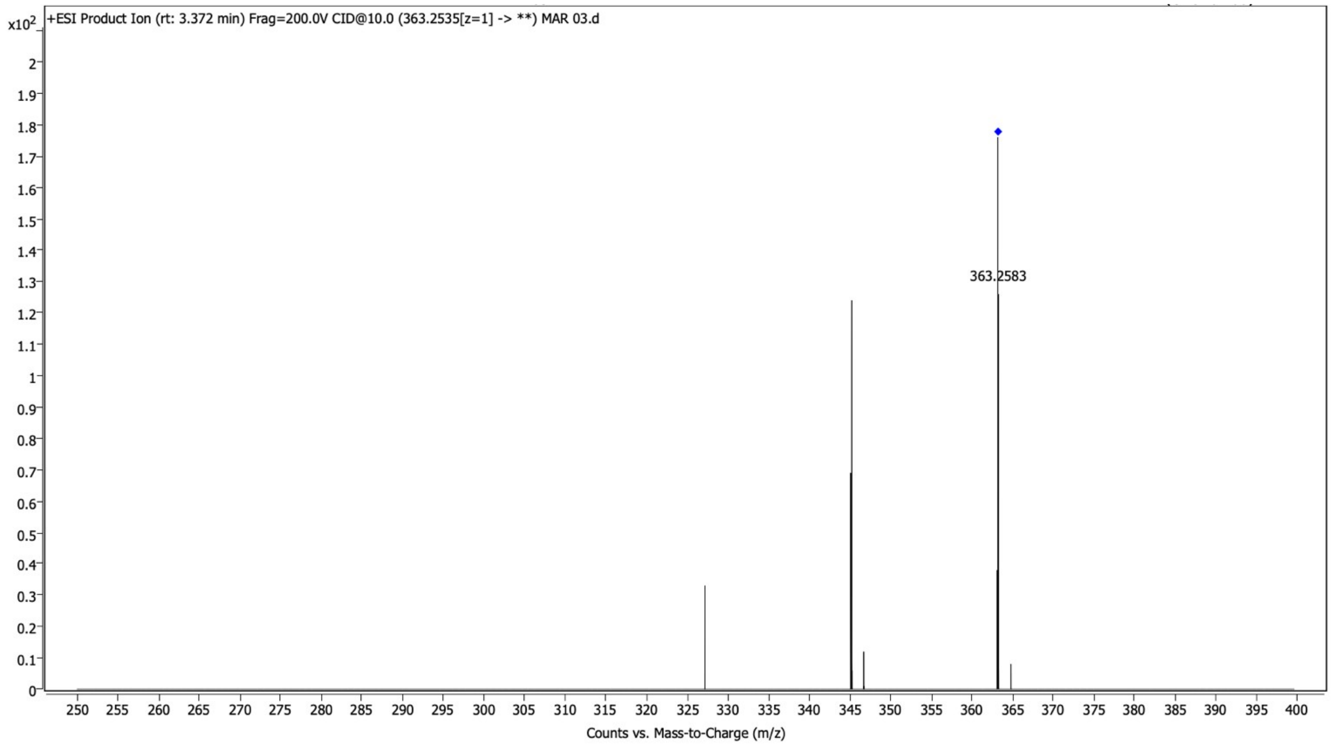

Figure S-11A. The HRESIMS spectrum of **9**.

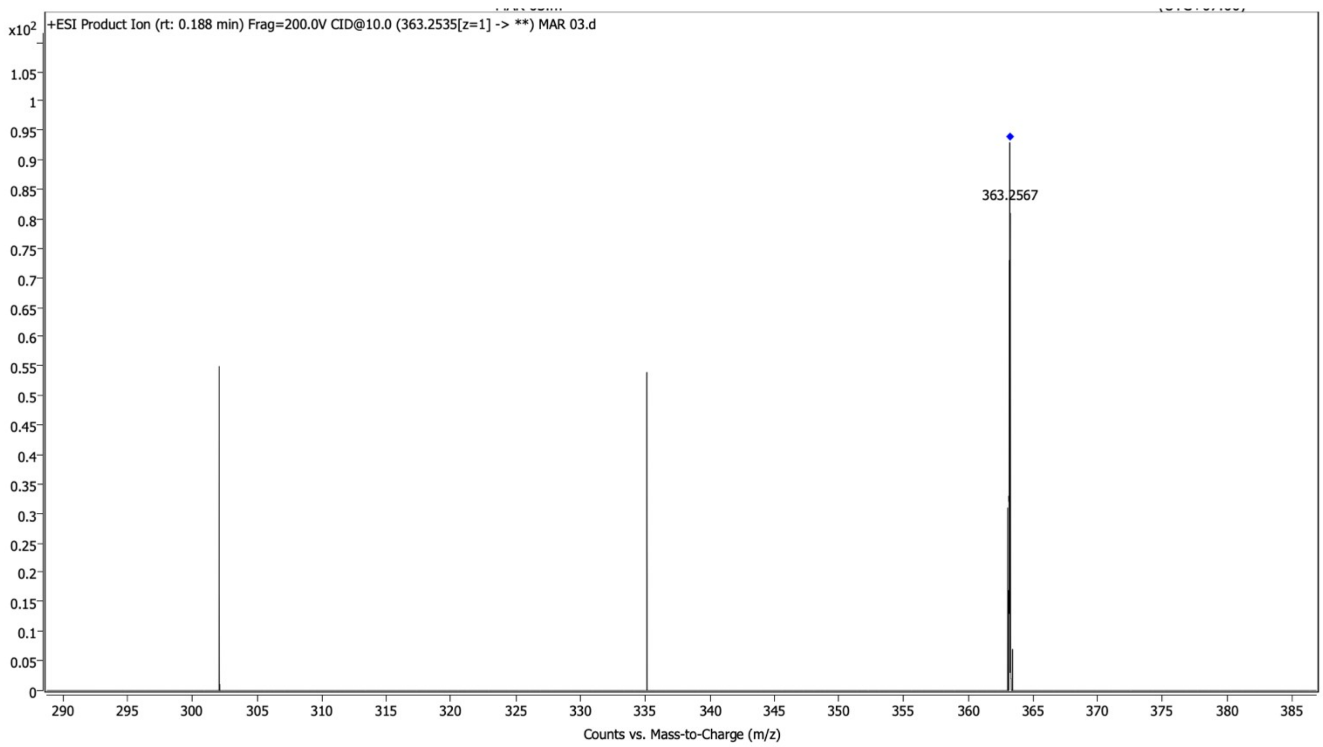

Figure S-11B. The HRESIMS spectrum of **9**.

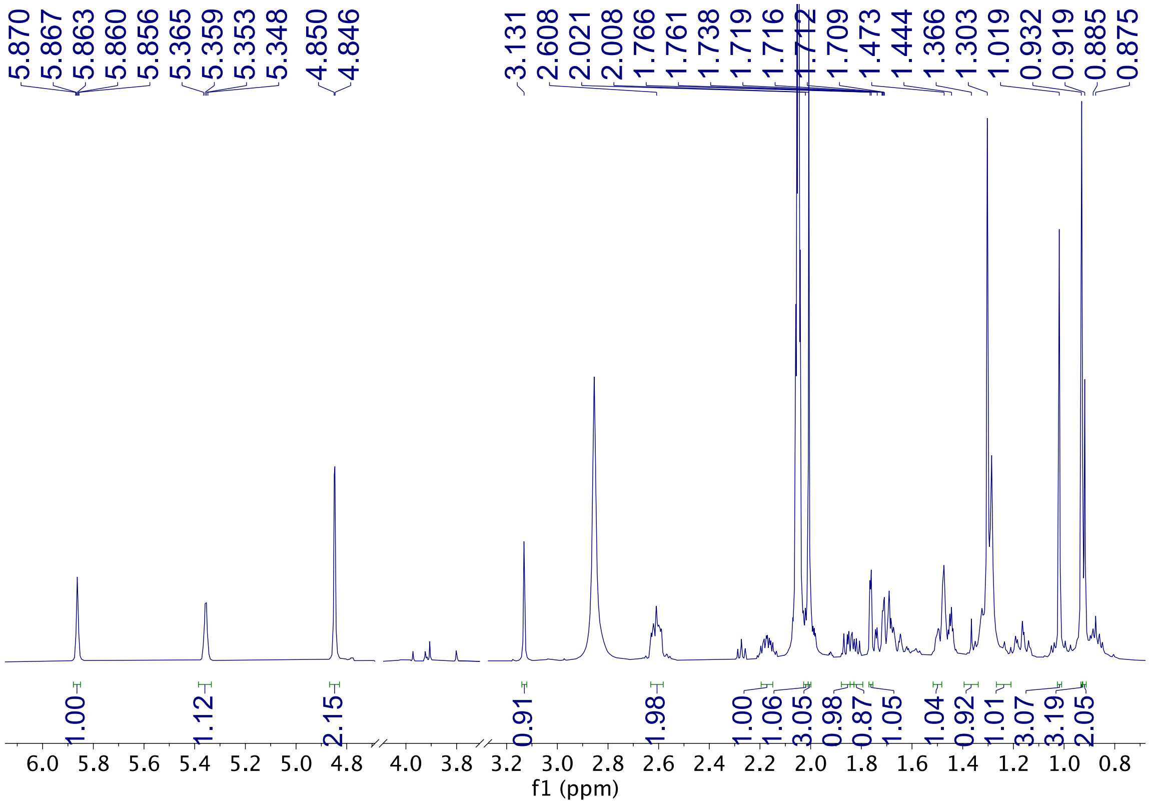

Figure S-11C. The ^{1}H NMR spectrum of **9** in acetone-d_6.

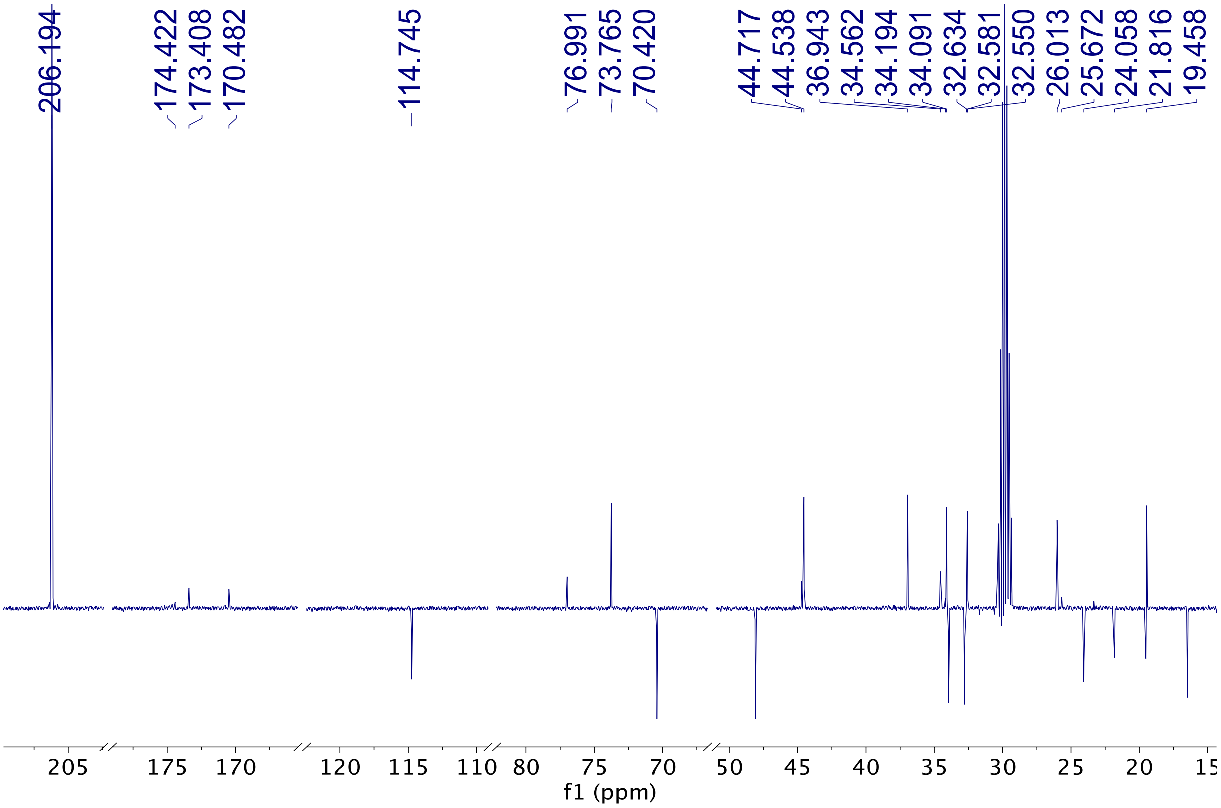

Figure S-11D. The ^{13}C NMR spectrum of **9** in acetone-d_6.

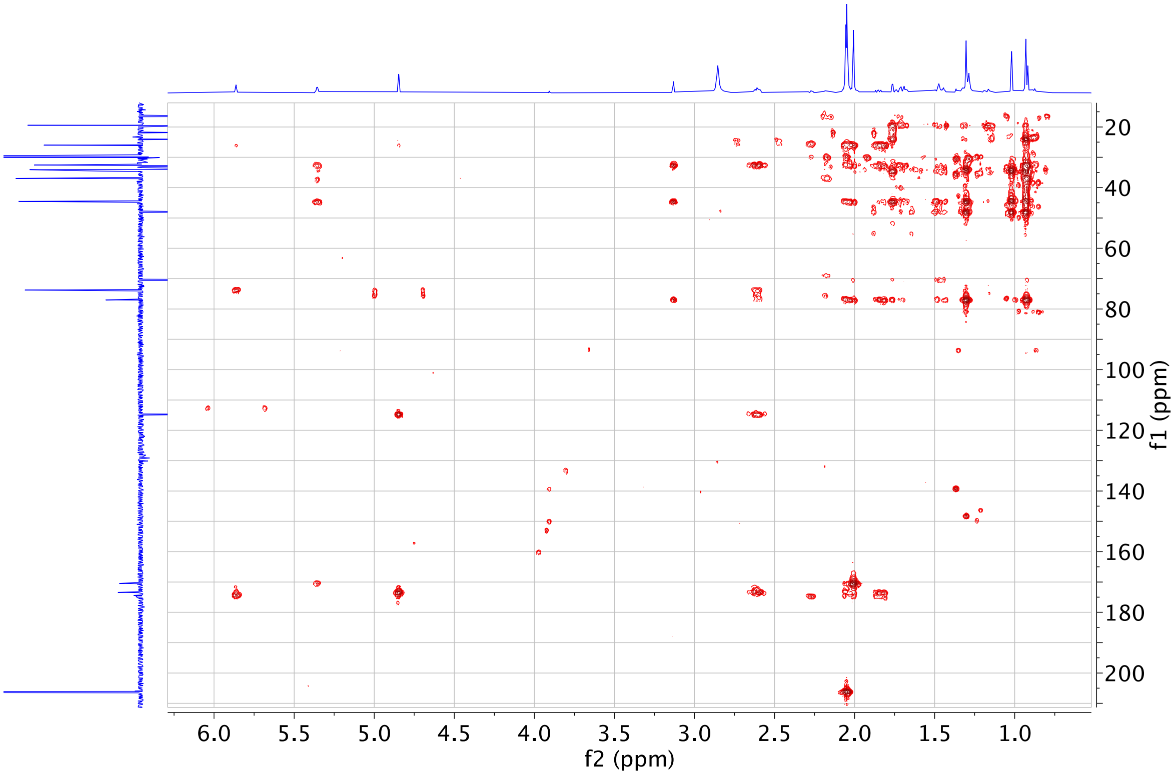

Figure S-11E. The HMBC spectrum of **9** in acetone-d_6.

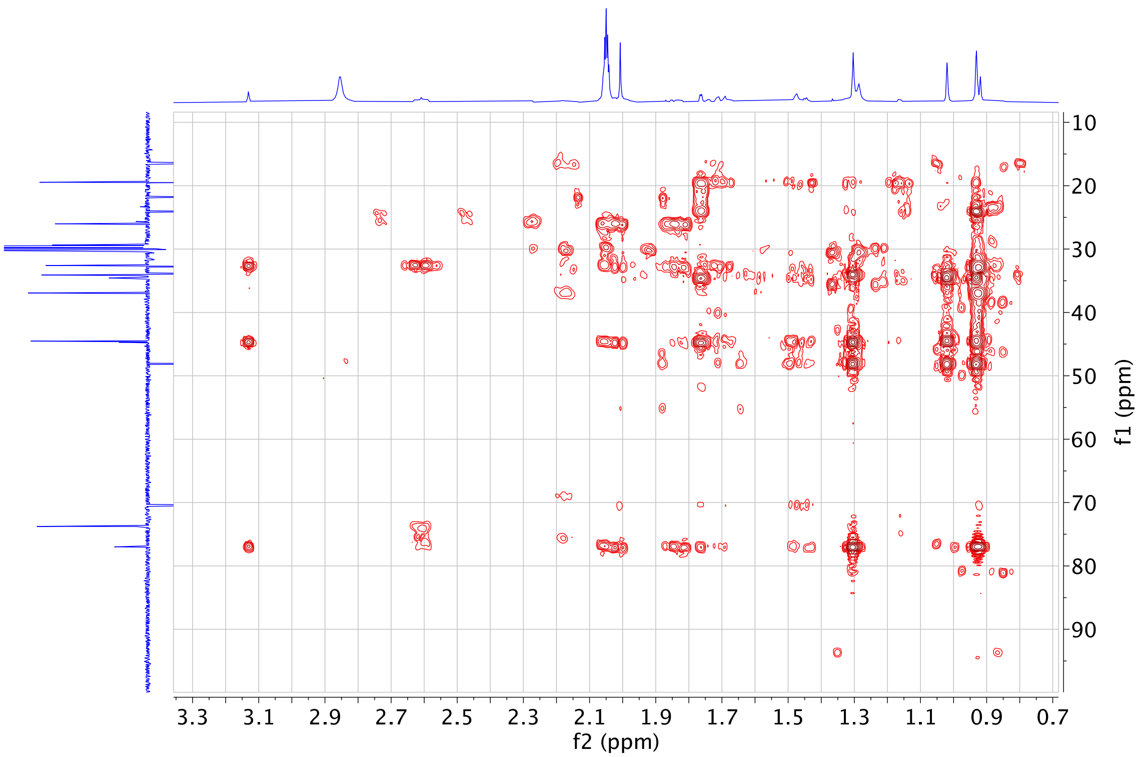

Figure S-11F. The HMBC spectrum of **9** in acetone-d$_{6}$.

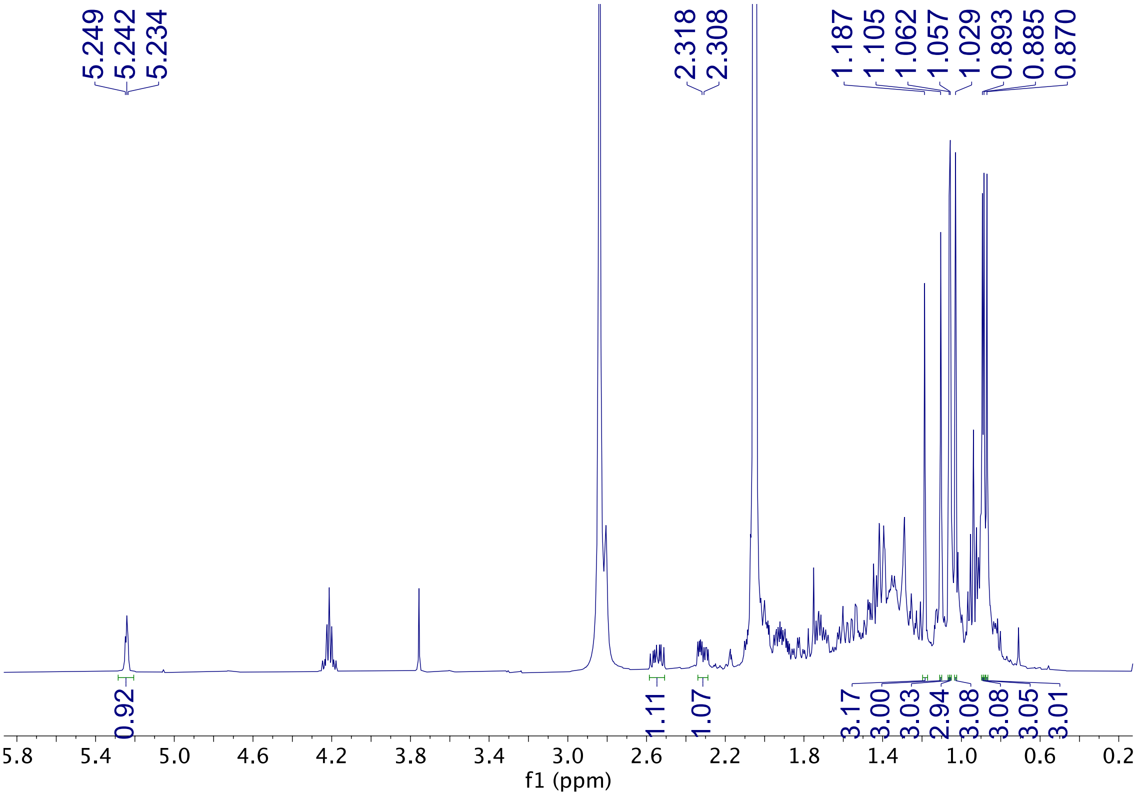

Figure S-12A. The ^{1}H NMR spectrum of **10** in acetone-d_{6}.

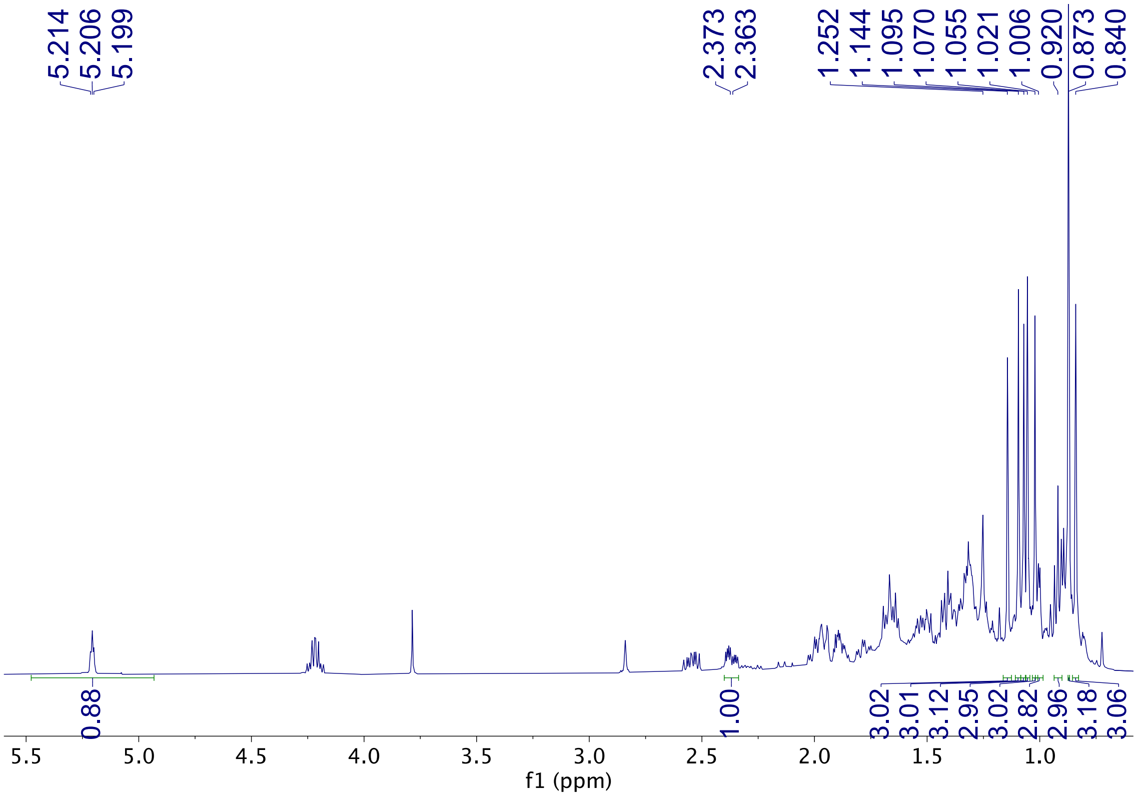

Figure S-12B. The ^{1}H NMR spectrum of **10** in $CDCl_3$.

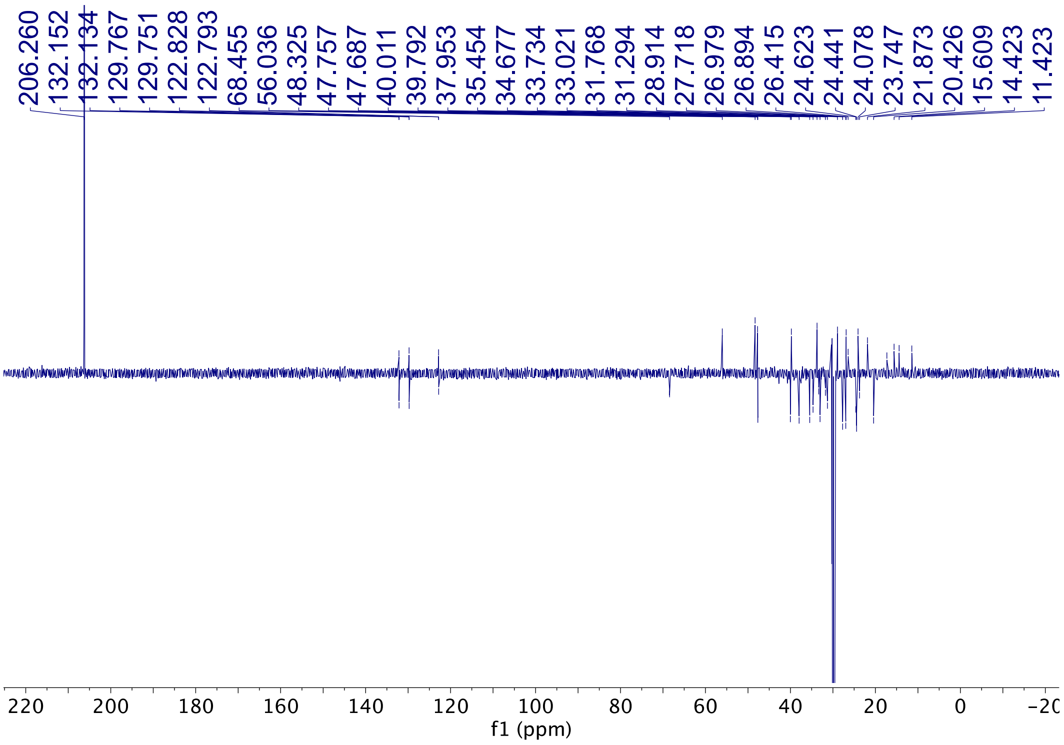

Figure S-12C. The ^{13}C NMR spectrum of **10** in acetone-d_6.

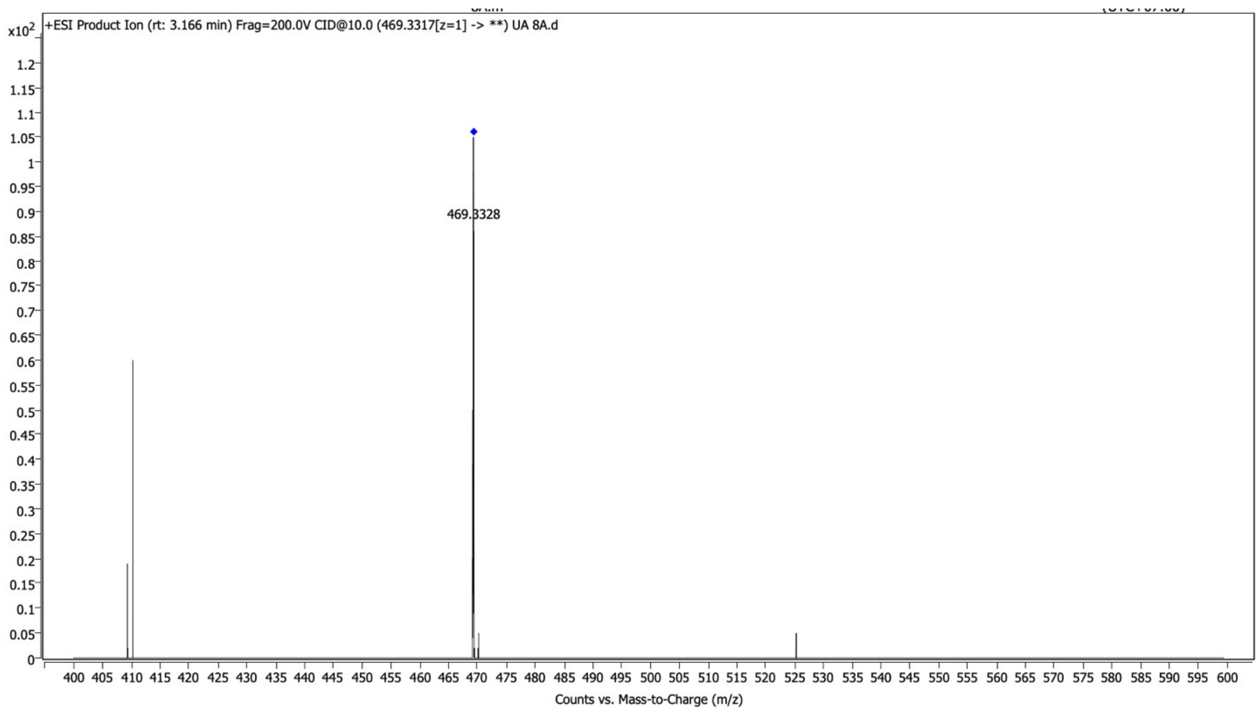

Figure S-13A. The HRESIMS spectrum of **11**.

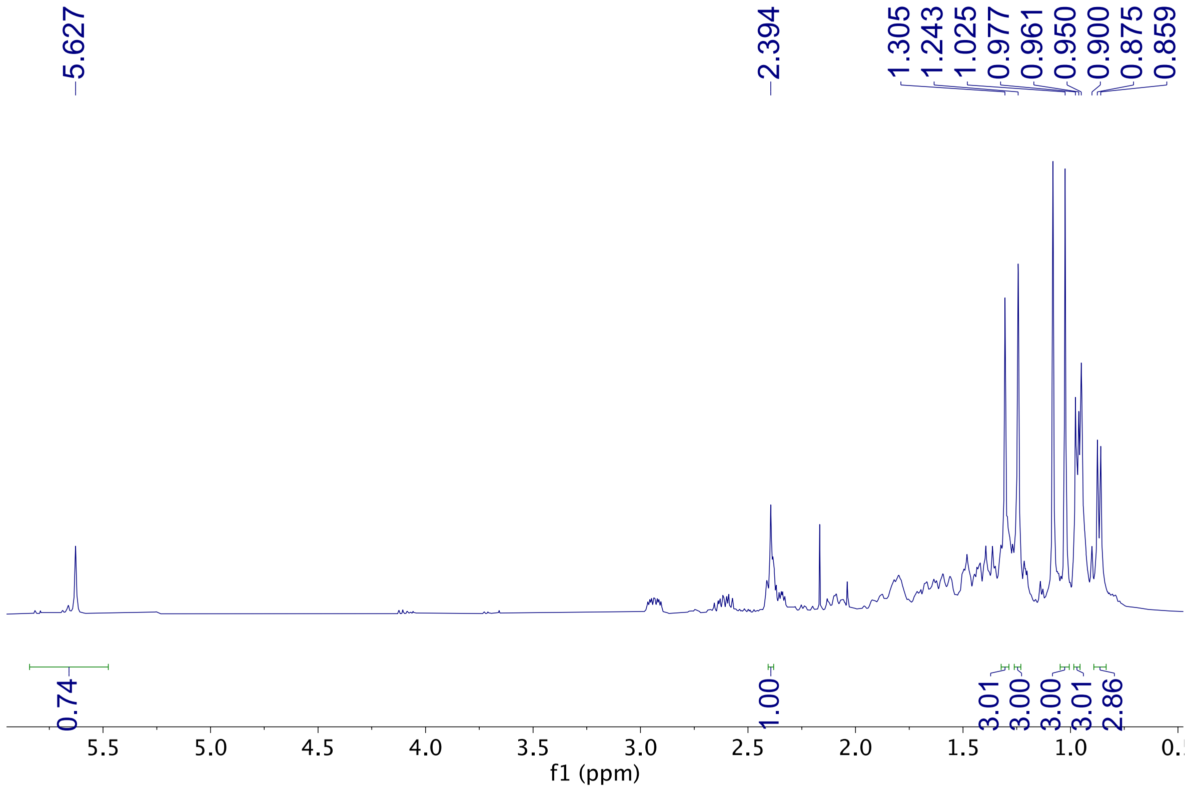

Figure S-13B. The ^{1}H NMR spectrum of **11** in $CDCl_3$.

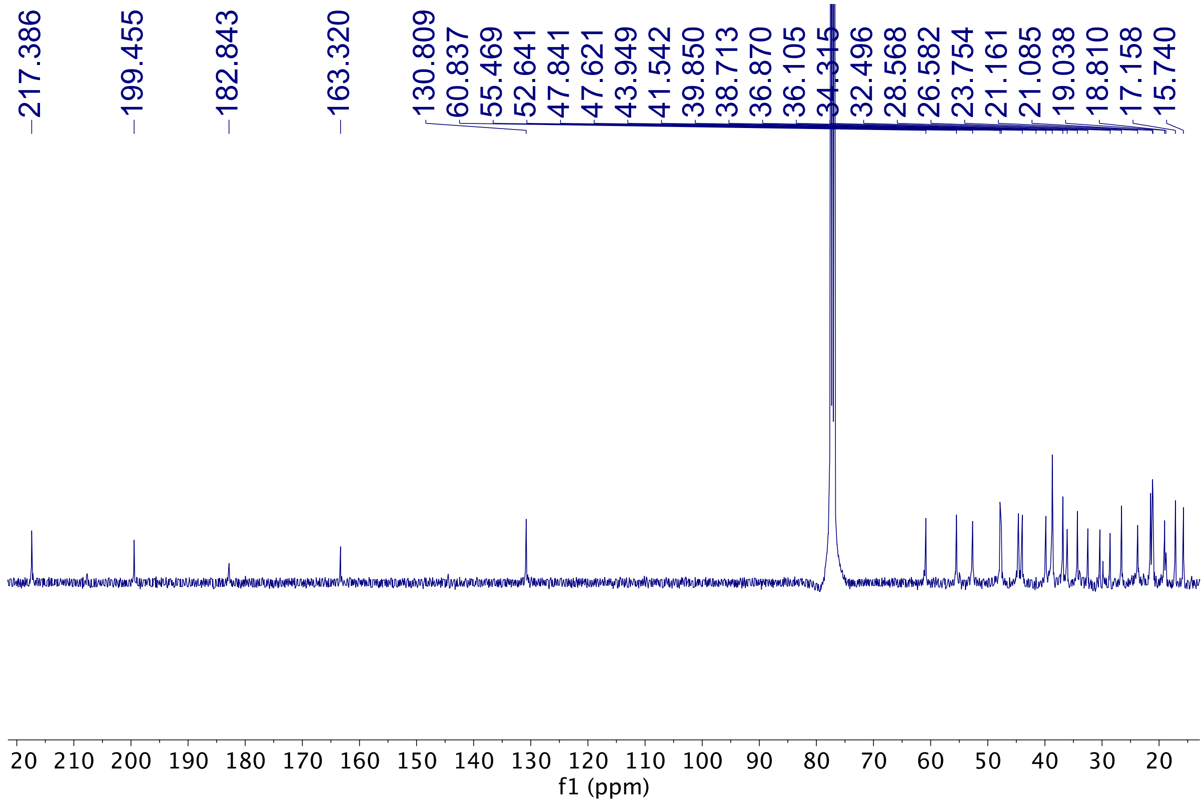

Figure S-13C. The ^{13}C NMR spectrum of **11** in $CDCl_3$.

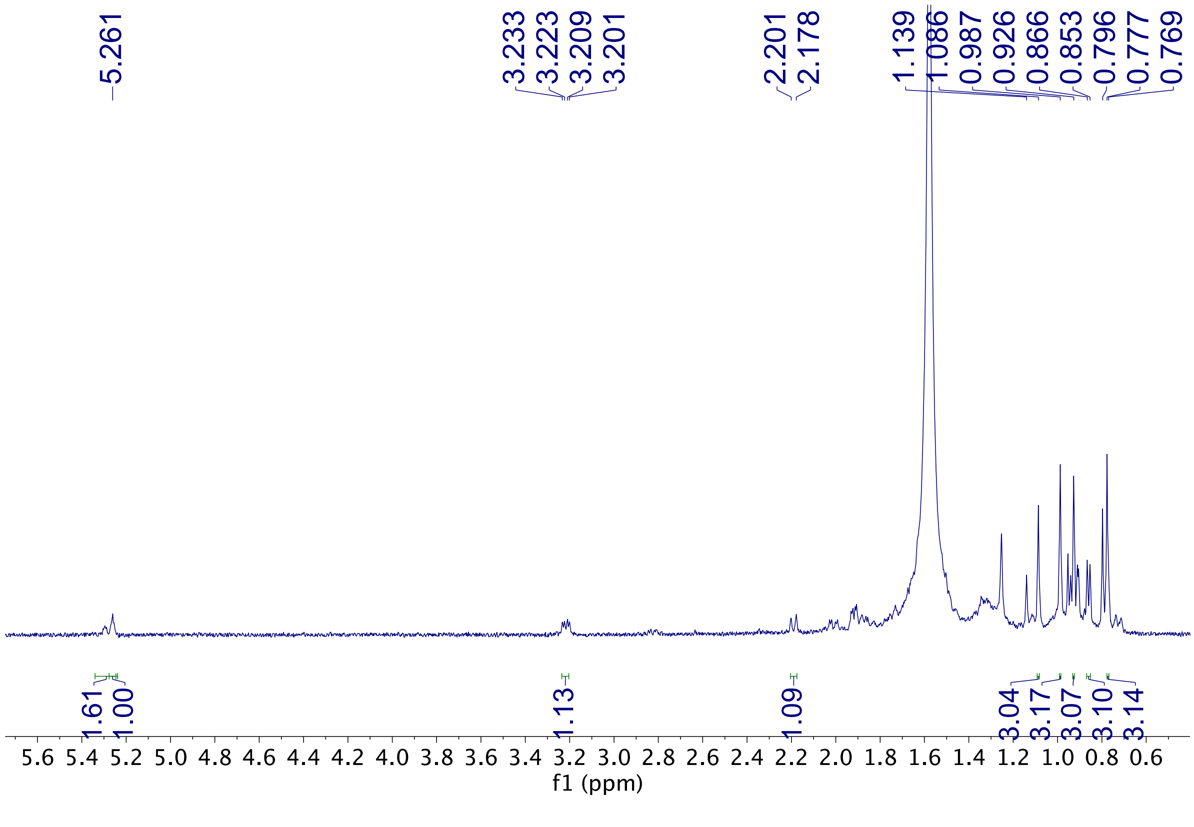

Figure S-14A. The ^{1}H NMR spectrum of **12** in $CDCl_3$.

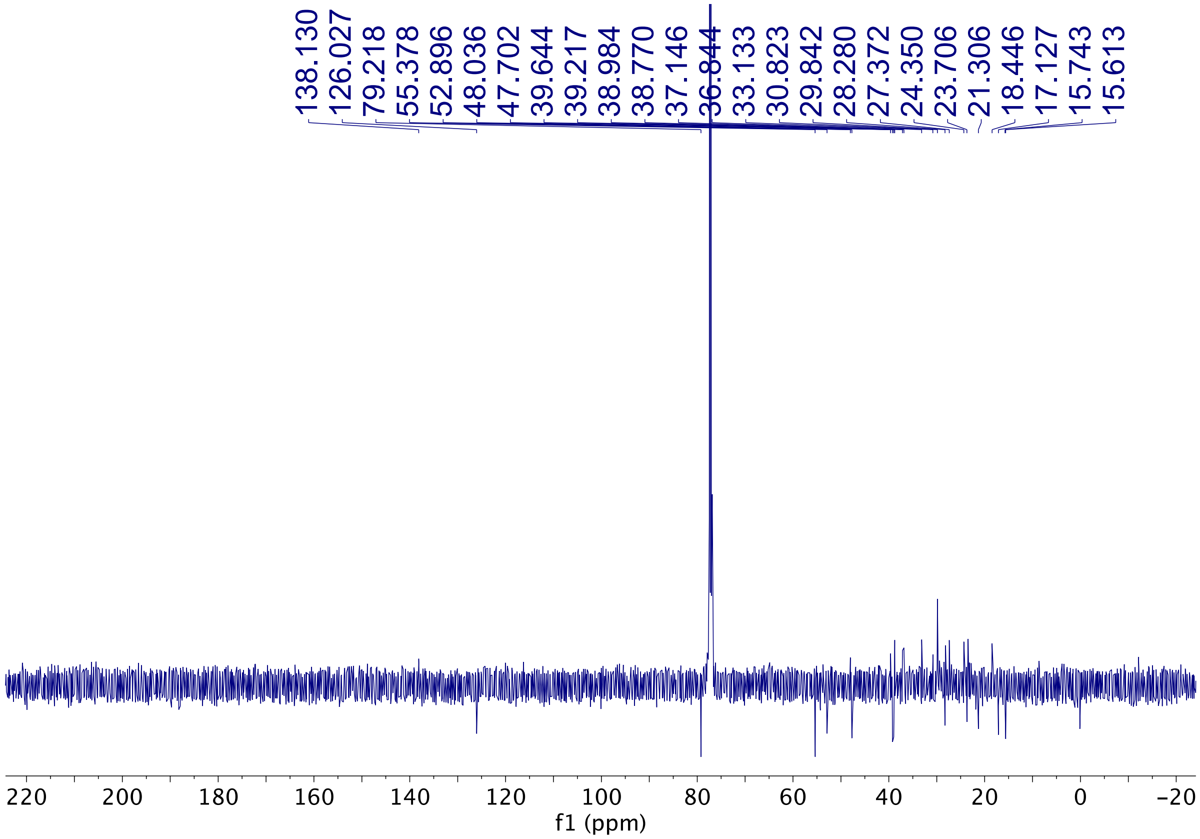

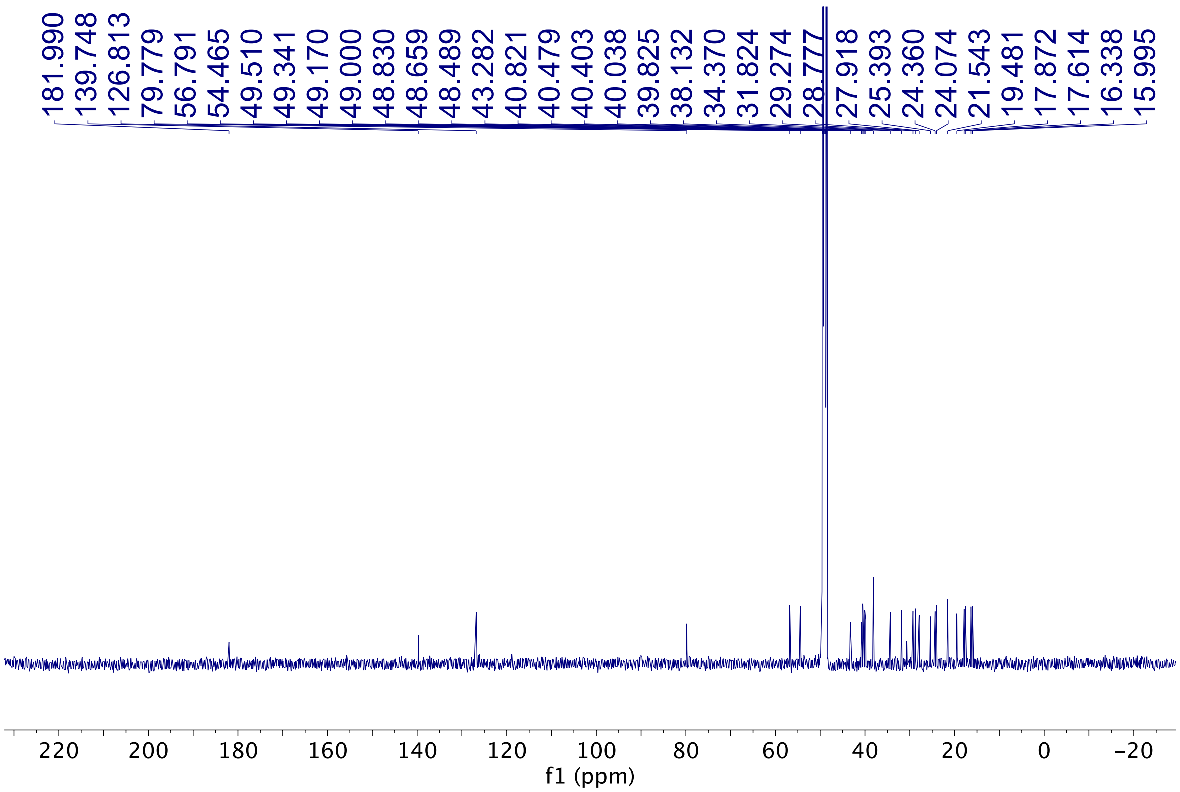

Figure S-14B. The ^{13}C NMR spectrum of **12** in $CDCl_3$.

Figure S-14C. The ^{13}C NMR spectrum of **12** in MeOD.

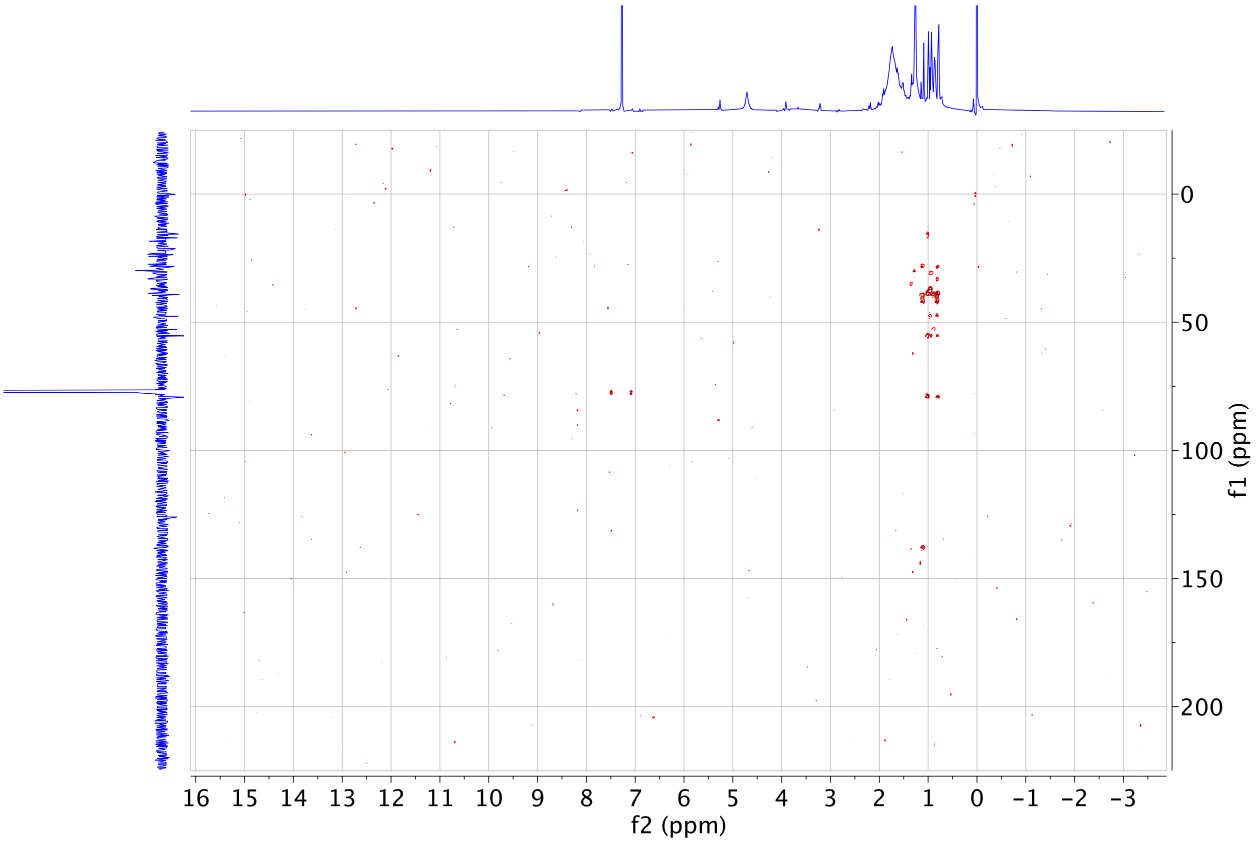

Figure S-14D. The HMBC spectrum of **12** in $CDCl_3$.

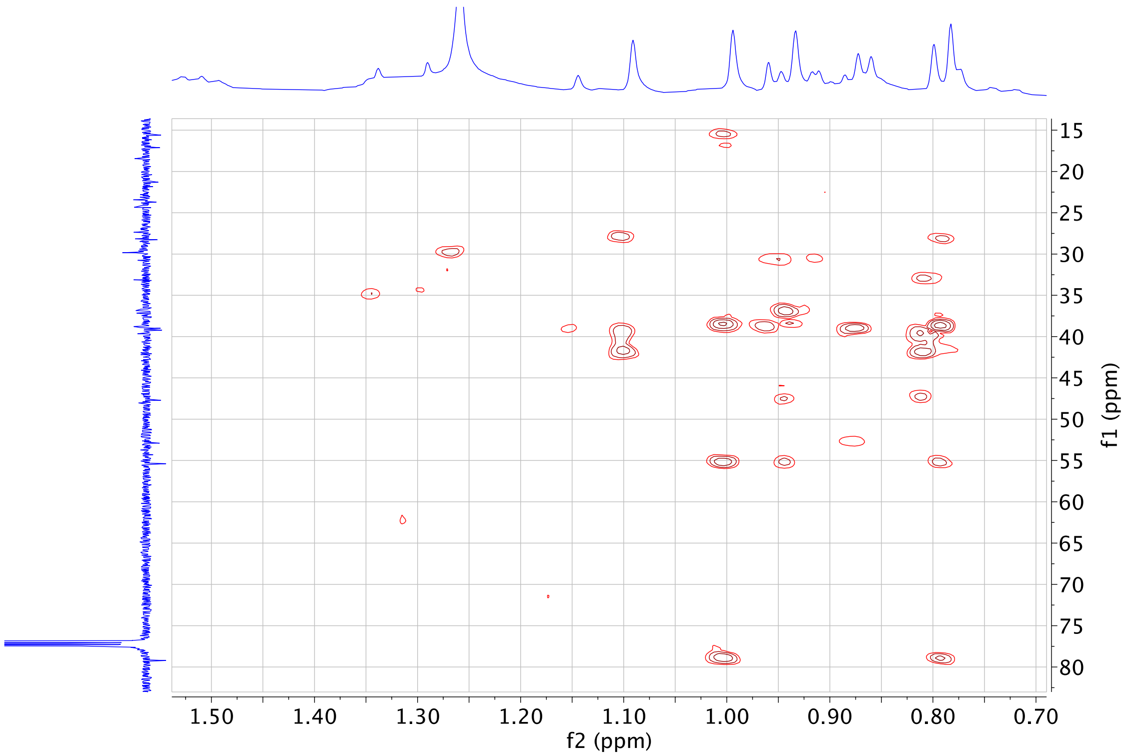

Figure S-14E. The HMBC spectrum of **12** in $CDCl_3$.

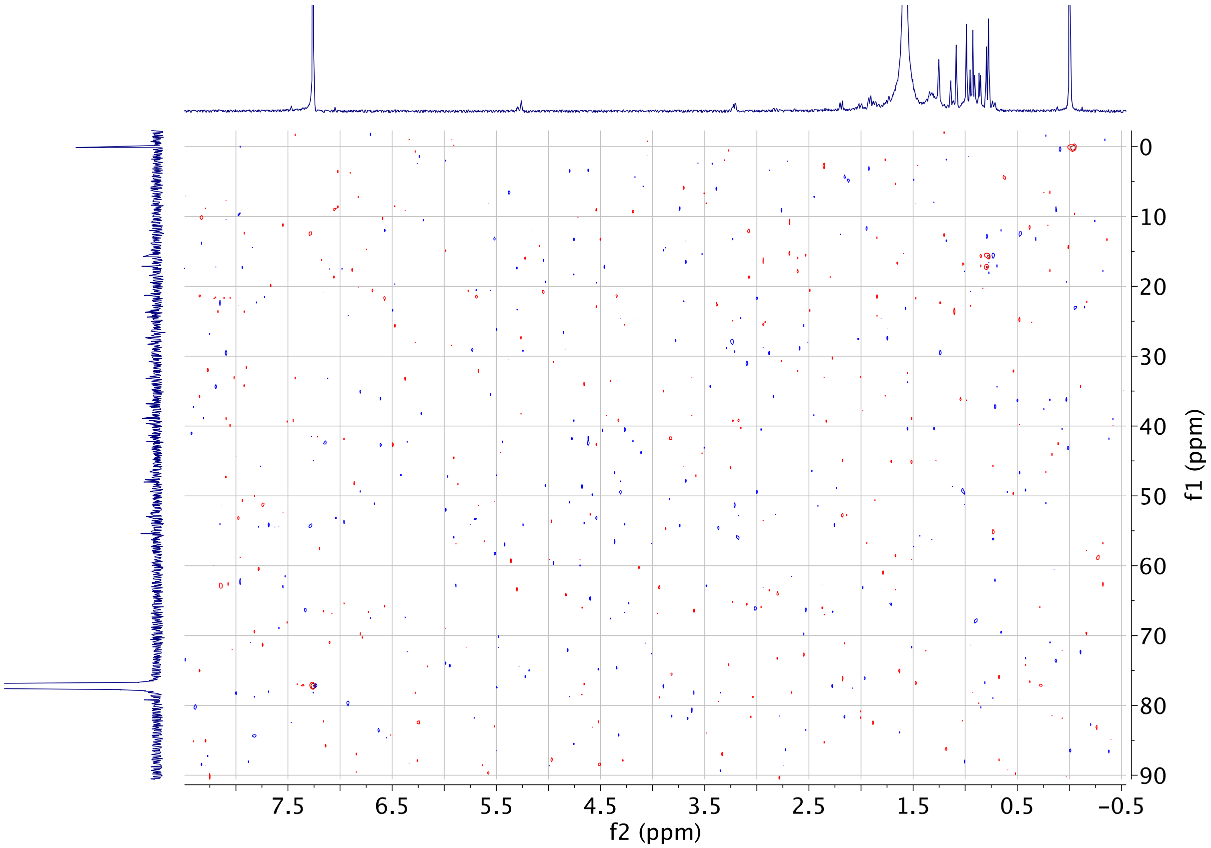

Figure S-14F. The HSQC spectrum of **12** in $CDCl_3$.

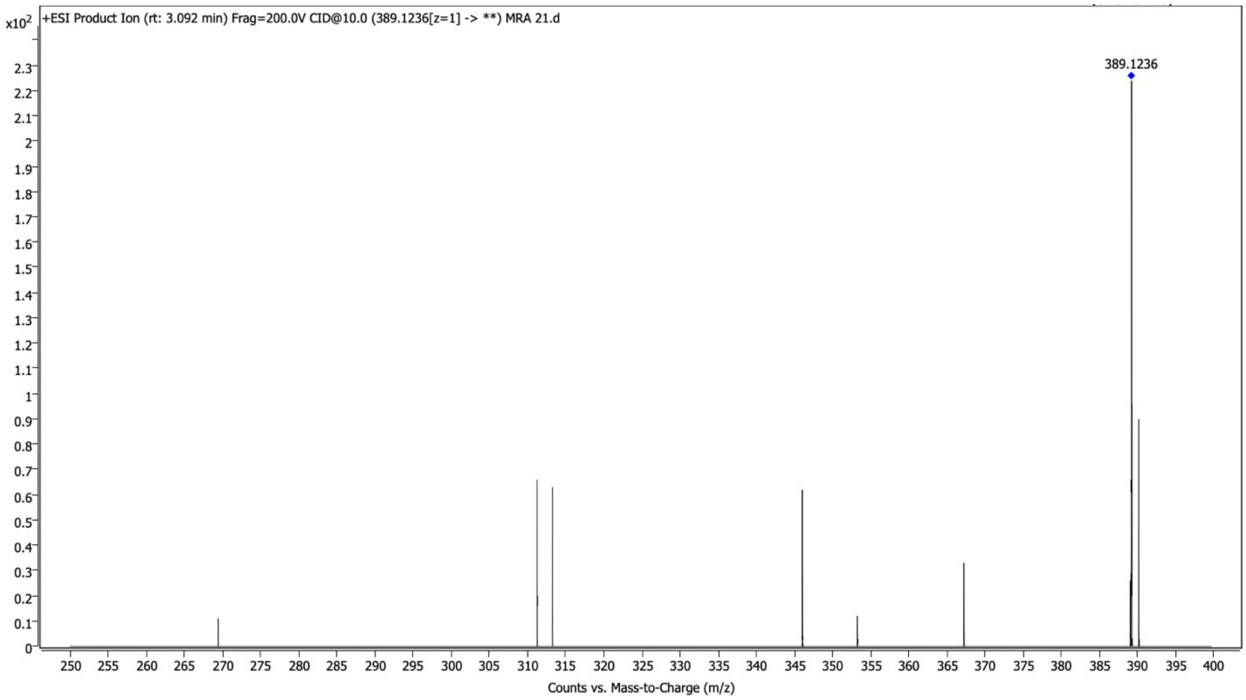

Figure S-15A. The HRESIMS spectrum of **13**.

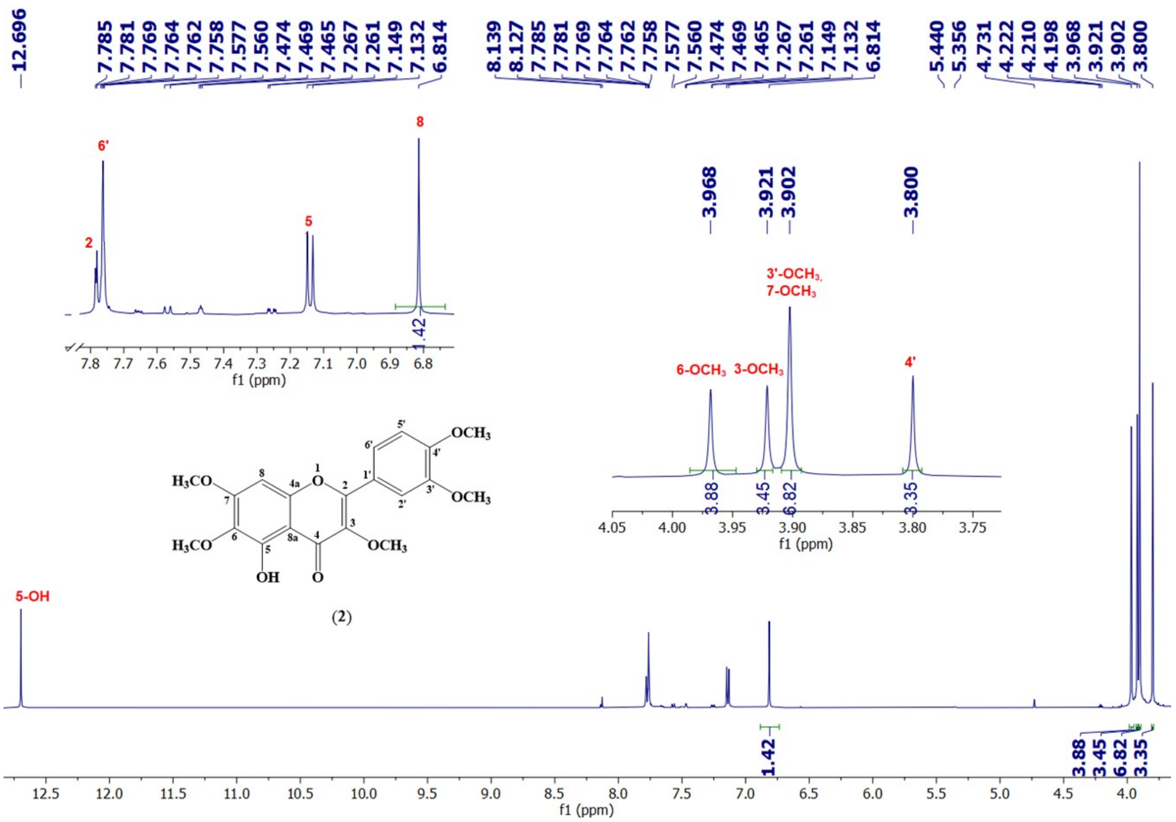

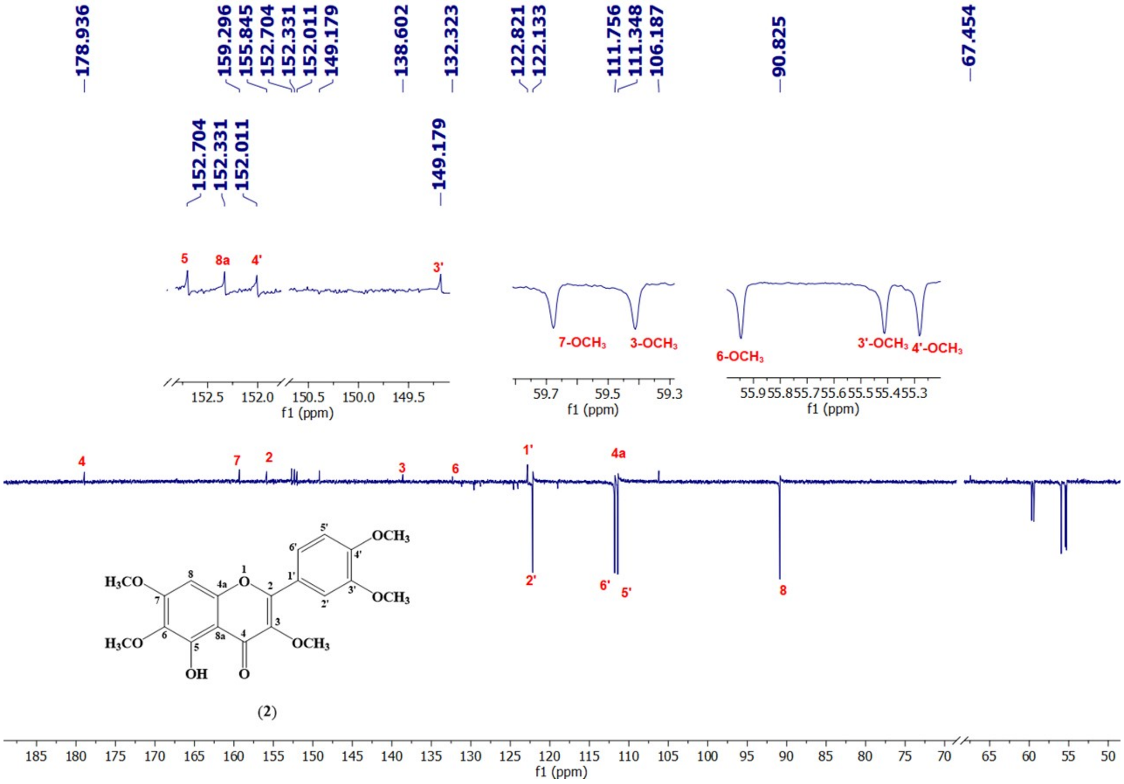

Figure S-15B. The ^{1}H NMR spectrum of **13** in acetone-d_6.

Figure S-15C. The ^{13}C NMR spectrum of **13** in acetone-d_6.

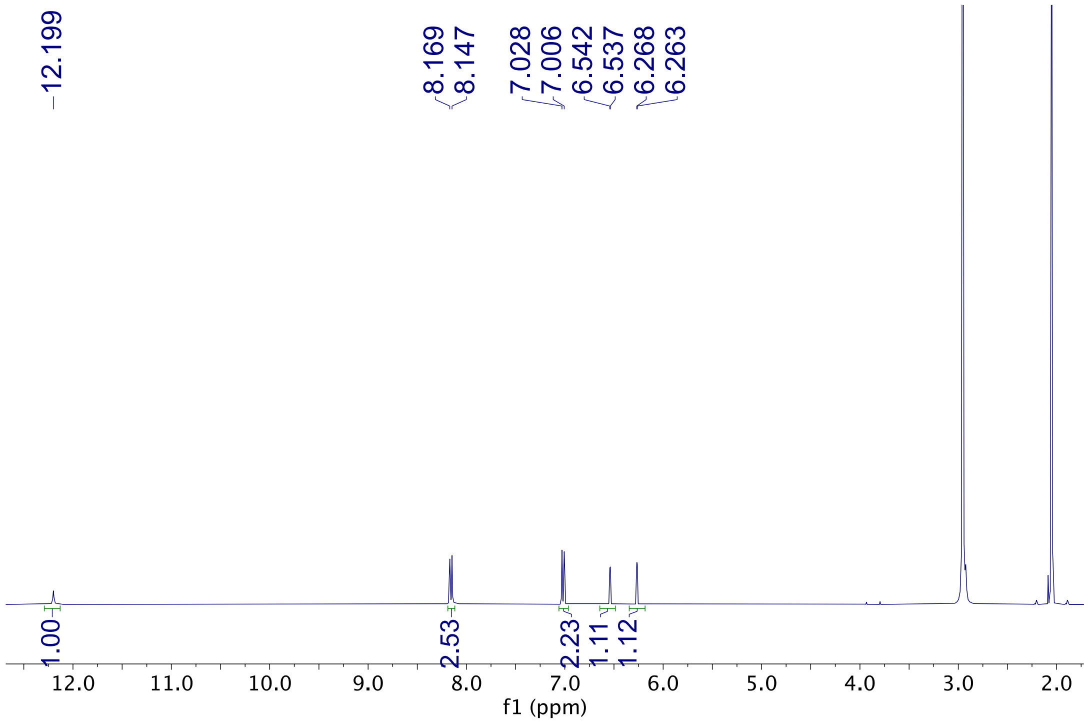

Figure S-16A. The ^{1}H NMR spectrum of **14** in acetone-d_6.

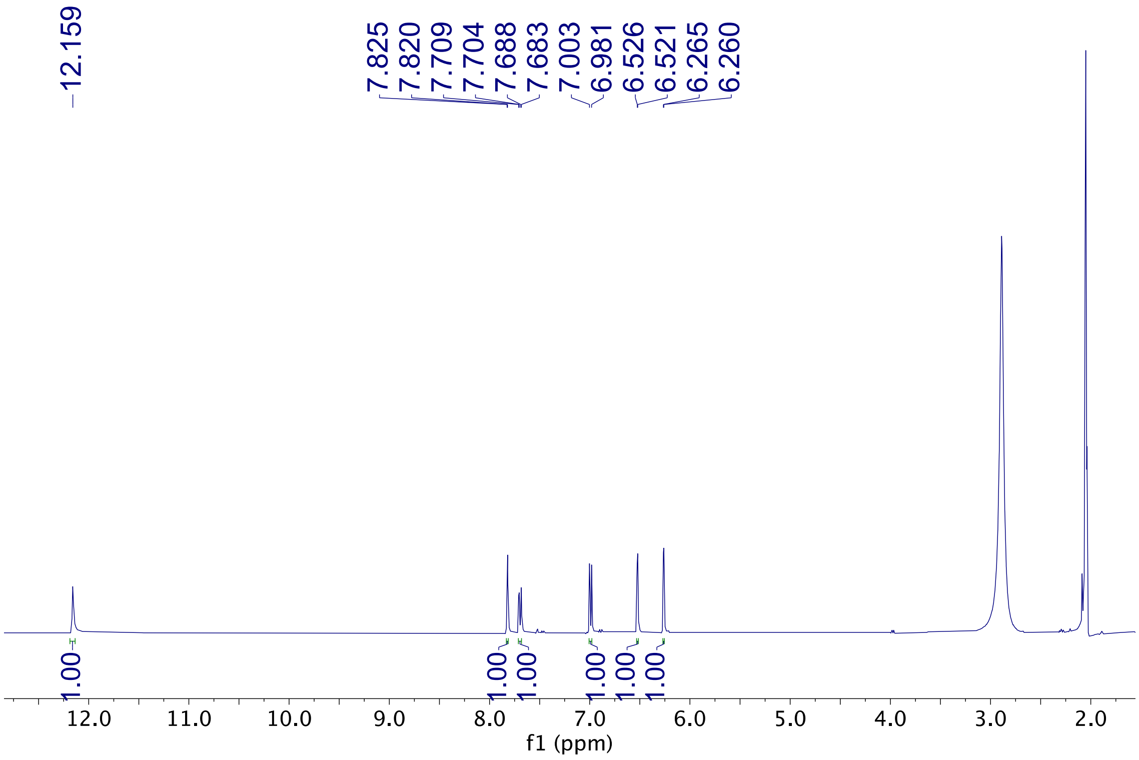

Figure S-17A. The ^{1}H NMR spectrum of **15** in acetone-d_6.

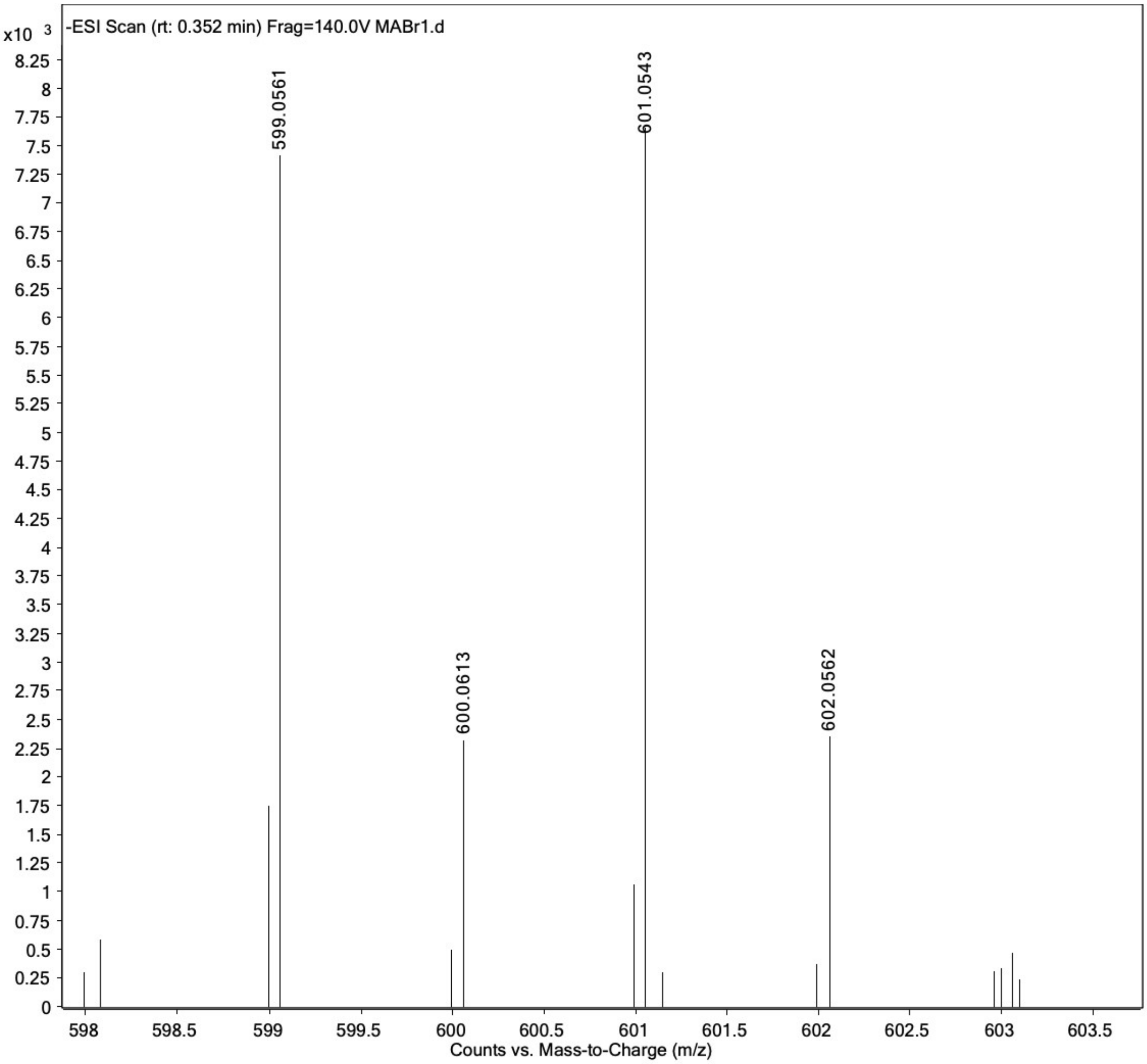

Figure S-18A. The HRESIMS spectrum of **1a.**

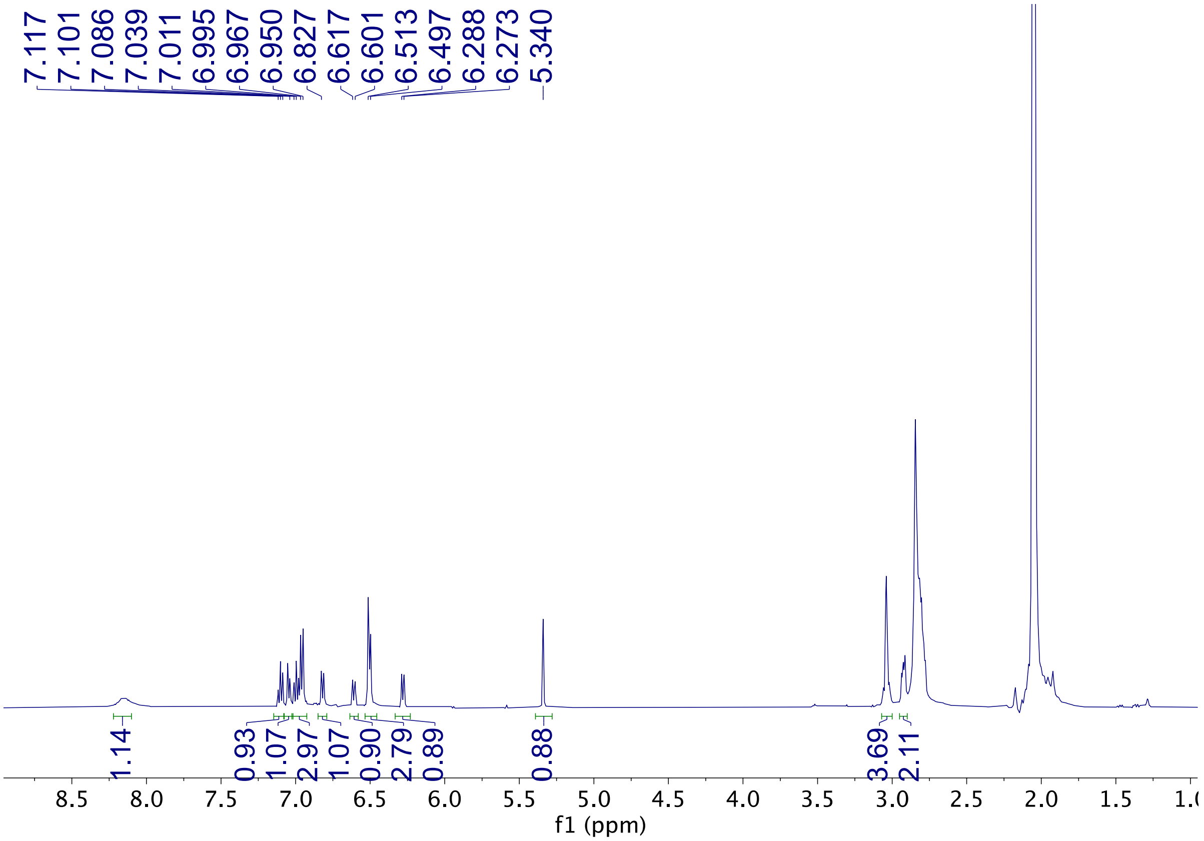

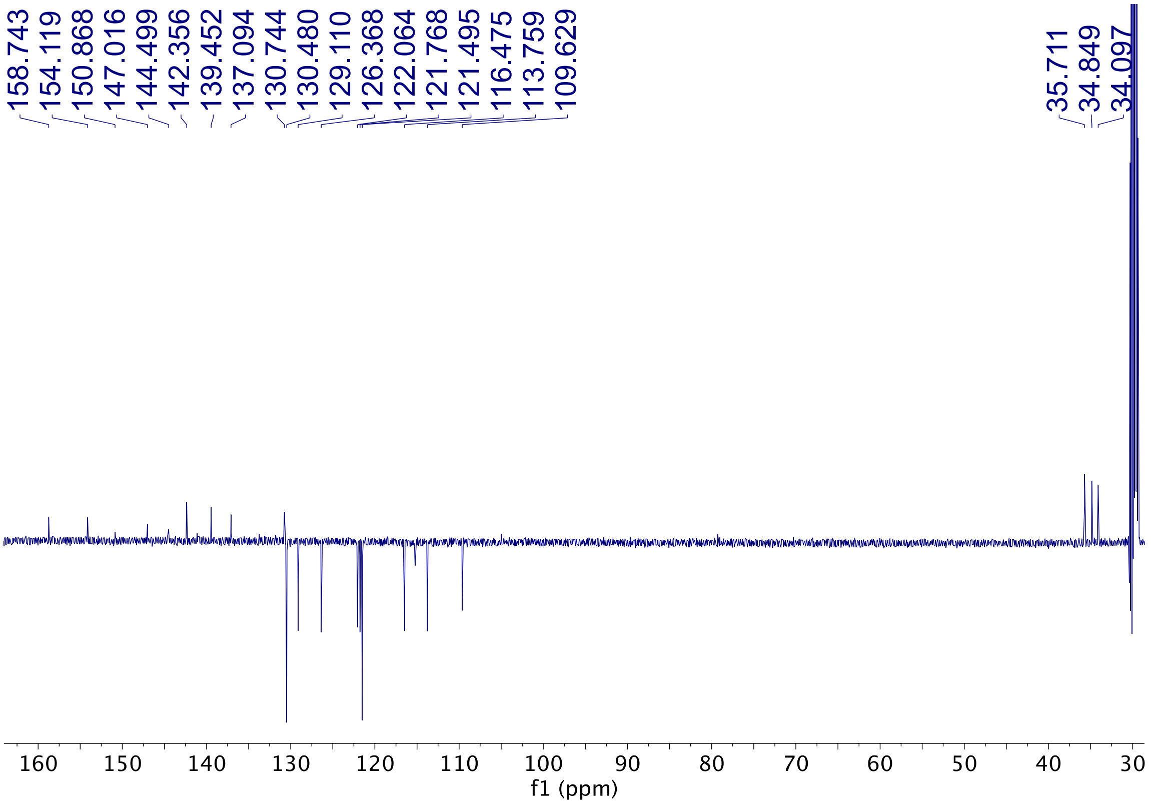

Figure S-18B. The ^{1}H NMR spectrum of **1a** in acetone-d_6.

Figure S-18C. The ^{13}C NMR spectrum of **1a** in acetone-d_6.

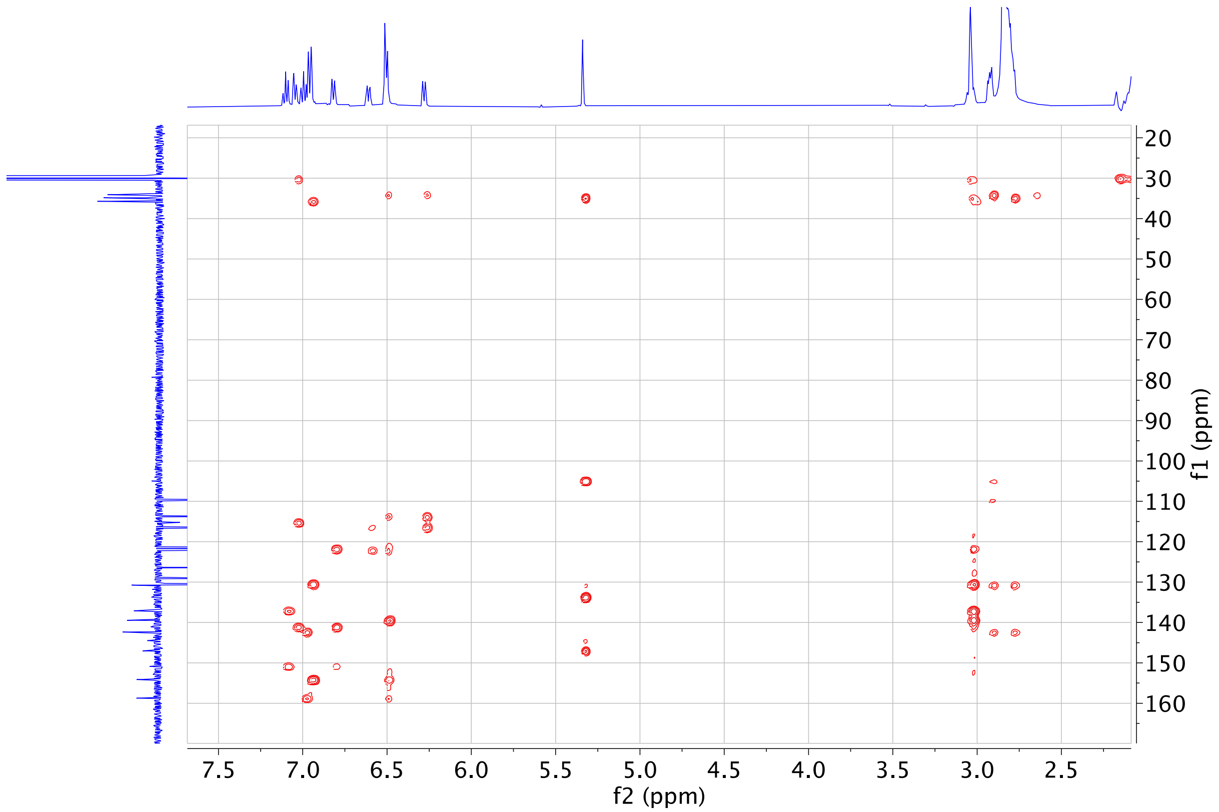

Figure S-18D. The HMBC spectrum of **1a** in acetone-d_6.

General procedure to synthesize compound 1a

In 1.0 mL of mixture of acetic acid, marchantin A (**1** 6.0 mg, 0.014 mmol) and sodium bromide (14.0 mg, 0.136 mmol) were dissolved at 80°C. 0.36 mL of 30% hydrogen peroxide (0.86 mmol) was added to the reaction mixture. The reaction was conducted for 2 hours. The resulting solution was neutralized with saturated sodium hydrogen carbonate, then extracted with ethyl acetate-water (1:1, v/v) to gain an organic layer. This layer was subsequently washed with brine three times, then dried and applied to silica gel CC, eluted with n-hexane-EtOAc (4:1, v/v) to obtain **1a** (4.45 mg, 43%).

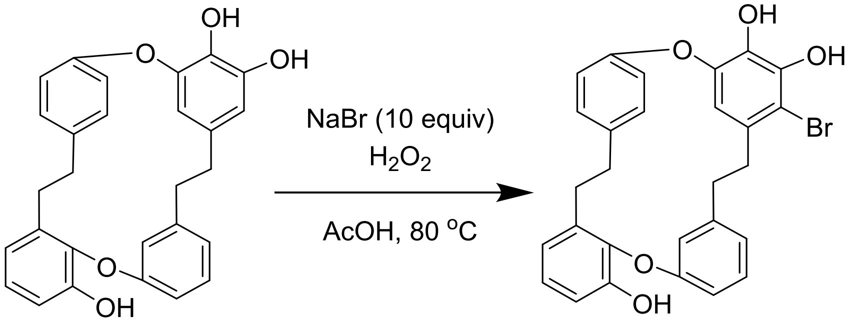

1 (marchantin A) **1a** (5'-bromomarchantin A)

Scheme S-1. General procedure to synthesize compound **1a**
